# MALDI-TOF peptidomic analysis of serum and post-prostatic massage urine specimens to identify prostate cancer biomarkers

**DOI:** 10.1186/s12014-018-9199-8

**Published:** 2018-07-25

**Authors:** Andrea Padoan, Daniela Basso, Carlo-Federico Zambon, Tommaso Prayer-Galetti, Giorgio Arrigoni, Dania Bozzato, Stefania Moz, Filiberto Zattoni, Rino Bellocco, Mario Plebani

**Affiliations:** 1 Department of Medicine-DIMED, University of Padova, Via Giustiniani 2, 35128 Padua, Italy; 2 Department of Biomedical Sciences, University of Padova, Padua, Italy; 3 Department of Surgical, Oncological and Gastroenterological Sciences, University of Padova, Padua, Italy; 4 Proteomic Center, University of Padova, Padua, Italy; 5 Department of Statistics and Quantitative Methods, University of Milano-Bicocca, Milan, Italy; 6 Department of Medical Epidemiology and Biostatistics (MEB), Karolinska Institute, Stockholm, Sweden

**Keywords:** Prostate cancer, Biomarkers, MALDI-TOF/MS, Data normalization, Measurement error, Regression calibration, SIMEX, Peptidomic profiling, Analytical variability, Serum, Urine

## Abstract

**Background:**

Lower urinary tract symptoms (LUTS) and prostate specific antigen-based parameters seem to have only a limited utility for the differential diagnosis of prostate cancer (PCa). MALDI-TOF/MS peptidomic profiling could be a useful diagnostic tool for biomarker discovery, although reproducibility issues have limited its applicability until now. The current study aimed to evaluate a new MALDI-TOF/MS candidate biomarker.

**Methods:**

Within- and between-subject variability of MALDI-TOF/MS-based peptidomic urine and serum analyses were evaluated in 20 and 15 healthy donors, respectively. Normalizations and approaches for accounting below limit of detection (LOD) values were utilized to enhance reproducibility, while Monte Carlo experiments were performed to verify whether measurement error can be dealt with LOD data. Post-prostatic massage urine and serum samples from 148 LUTS patients were analysed using MALDI-TOF/MS. Regression-calibration and simulation and extrapolation methods were used to derive the unbiased association between peptidomic features and PCa.

**Results:**

Although the median normalized peptidomic variability was 24.9%, the within- and between-subject variability showed that median normalization, LOD adjustment, and log_2_ data transformation were the best combination in terms of reliability; in measurement error conditions, intraclass correlation coefficient was a reliable estimate when the LOD/2 was substituted for below LOD values. In the patients studied, 43 peptides were shared by the urine and serum, and several features were found to be associated with PCa. Only few serum features, however, show statistical significance after the multiple testing procedures were completed. Two serum fragmentation patterns corresponded to the complement C4-A.

**Conclusions:**

MALDI-TOF/MS serum peptidome profiling was more efficacious with respect to post-prostatic massage urine analysis in discriminating PCa.

**Electronic supplementary material:**

The online version of this article (10.1186/s12014-018-9199-8) contains supplementary material, which is available to authorized users.

## Background

Mainly affecting adult men, lower urinary tract symptoms (LUTS) can be caused by prostate-related [e.g. benign prostatic hyperplasia (BPH)] and non prostate-related conditions [e.g. bladder dysfunction] [1]. It has been seen that the prevalence of LUTS is strongly age-dependent as it affects 13.4 and 31.5% of males between 35–39 and 70–80, respectively [2].

The association between LUTS and prostate cancer (PCa) has been extensively debated in light of the fact that LUTS has long been considered a potential early clinical manifestation of PCa. Currently no reliable biomarkers are capable of discriminating between PCa and benign conditions in patients with LUTS. In fact, prostatic specific antigen (PSA), the most commonly used screening tool for PCa, has a relatively poor specificity for PCa with respect to other benign prostatic diseases [3, 4]. Recently, prostate cancer antigen 3 (PCA3), which is based on the quantification of both PCA3 and PSA mRNA expression in urine samples, has been shown to outperform PSA in identifying patients at risk for PCa at the first biopsy and to be useful in predicting the outcome of re-biopsy after the first biopsy [5].

The discovery of new potential peptide/protein biomarkers might be efficiently performed by LC–MS, since it provides the structure information of peptides and is convenient for data analysis. Another proteomic approach, such as matrix-assisted laser desorption ionization time-of-flight mass spectrometry (MALDI-TOF/MS), may be used in laboratory practice because of its unique capability of generating peptide/protein profiles of biological fluids without extensive sample manipulation. The advantages of MALDI-TOF/MS profiling include the high throughput screening of ions and the possibility of identifying sets of unique low molecular weight (LMW) features as well as panels of multiple biomarkers for disease detection, cancer in particular [6]. Its major drawbacks, nevertheless, include: (1) sample preparation issues (pre-analytical), (2) analytical variability as calibration can vary over time as can crystallization conditions, etc. (analytical), (3) the complexity of the bioinformatics procedures for peak detection and data analysis (post-analytical), and (4) the lack of an immediate identification of the molecular nature (peptide/protein, lipid, metabolite, glycan, etc.) of the identified peaks and the presence of ion adducts (H^+^, Na^+^, K^+^, NH^3+^) with peptides [7–12].

Another important consideration concerning MALDI-TOF/MS profiling studies regards the necessity of quantitatively comparing features derived from a series of different spectra (from different subjects). The label-free approach used in MALDI-TOF/MS profiling implies that features’ signals, rather than peptide levels, are normally considered. Our group has previously demonstrated that MALDI-TOF/MS reliability is strongly affected by the peak detection method and that its reproducibility can be improved by handling features’ signals appropriately, by adjusting the instrumental limit of detection (LOD) signal, and by applying median normalization to the data [12]. Despite the promising results of these approaches, the amount of measurement error in MALDI-TOF/MS peptidomic profiling has remained high [almost 20% for the intra-assay coefficient of variation (CV)], and continues to complicate data analysis.

According to the measurement error theory, the classical error model postulates that true biomarker levels cannot be obtained directly [13]. Interestingly, when biomarker levels are related to disease by regression analyses, measurement error usually causes a “bias toward the null” effect on the estimated coefficients [14]. Regression calibration (RCAL) and the simulation and extrapolation (SIMEX) statistical methods have been demonstrated to efficiently adjust the biased regression coefficients [14]. As only a few studies have been carried out to evaluate the effect of LOD issues on RCAL and SIMEX, analytical models need to be validated for their applicability [15, 16].

In view of these considerations and in order to improve data reliability, we performed a series of experimental studies to examine different strategies evaluating the variability of MALDI-TOF/MS-based peptidomic analyses of urine and serum. The approaches that resulted feasible for MALDI-TOF/MS analyses were then used. Serum and urine samples of patients presenting LUTS, with or without histologically proven PCa, were evaluated using MALDI-TOF/MS. Their discriminatory peptidomic features were compared with those of free prostate specific antigen (fPSA), total PSA (tPSA), free to total PSA (f/tPSA) and PCA3. RCAL and SIMEX were used to estimate the unbiased relationship between the presence of PCa and the features MALDI-TOF/MS based urinary and serum samples.

## Methods

### Study design

**Fig. 1:**
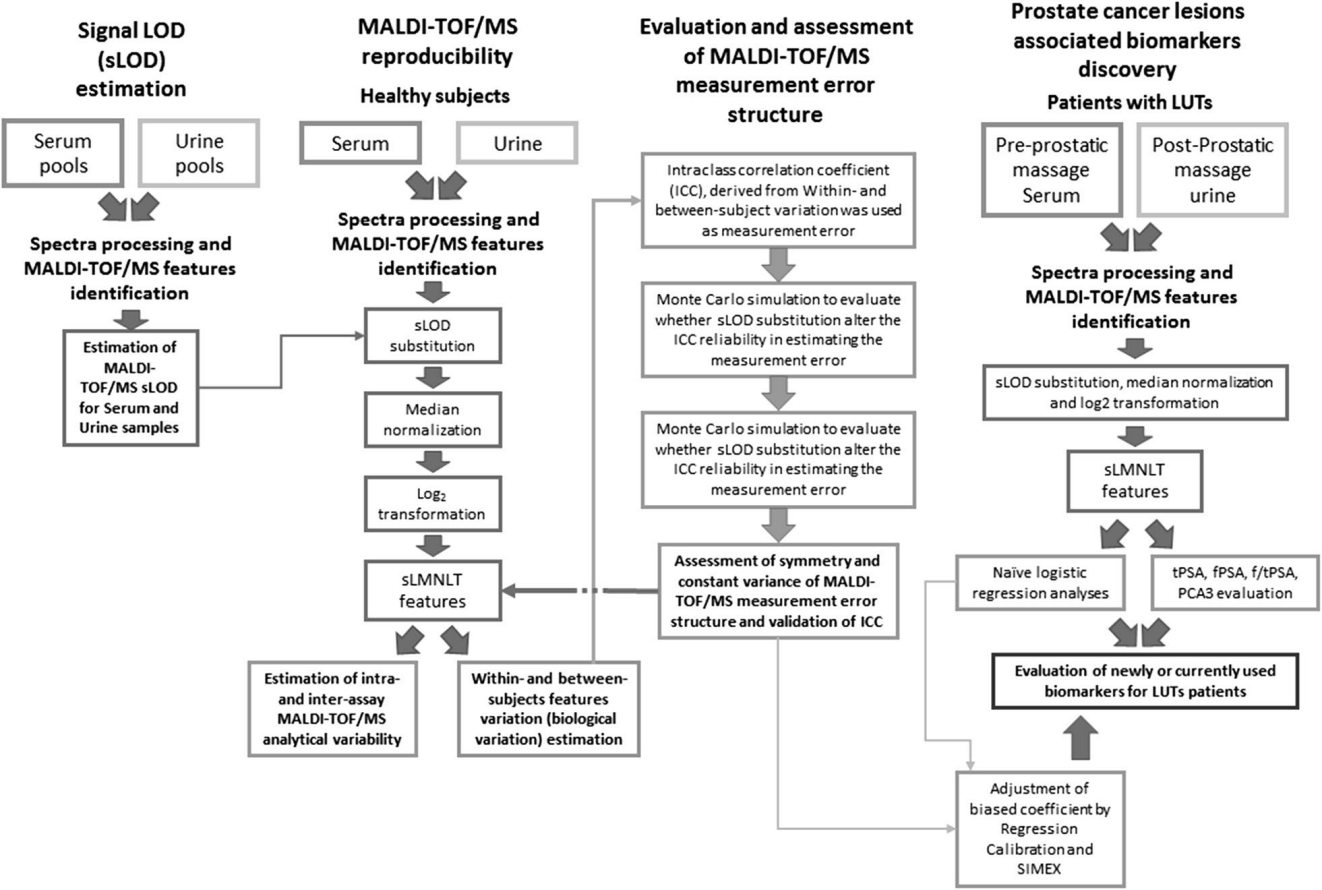
Workflow schematic of the study

The flow-chart of this study, illustrated in Fig. 1, included four main steps. The analyses of MALDI-TOF/MS reproducibility, signal LOD estimation, evaluation and assessment of measurement error were pre-requisites for the last phase of the study, i.e. the discovery of PCa associated MALDI-TOF/MS features.

### Within- and between-subjects variability of serum MALDI-TOF/MS-based peptidome and analytical variability of serum MALDI-TOF/MS-based features

Within- and between-subjects variability of MALDI-TOF/MS-based peptidome features were evaluated using fasting serum specimens collected from fifteen donors (8 males and 7 females; age range = 23–39 years). Two to three serum samples per subject were collected at different times over a time period ranging from 1 to 3 weeks. At least three aliquots of 500 μL of serum, prepared as described below, were immediately stored at − 80 °C for less than 3 months before the analysis. The specimens obtained from each subject were thawed, processed, and spotted in quintuplicate in a MALDI-TOF/MS plate in the same analytical session. A total of 2000 shots were then collected for each sample. After spectra processing, the ANOVA method was used to calculate within- and between-subject variability and the intraclass correlation coefficient (ICC).

To evaluate the analytical variability of MALDI-TOF/MS features, 500 μL of serum from three different subjects were pooled together, divided into smaller aliquots of 50 μL and immediately stored at − 80 °C for less than 3 months. Then a 5 × 5 experimental design was followed: every day for 5 consecutive days, a single aliquot of pooled serum was thawed, processed and spotted in quintuplicate for 5 times. Two thousand shots were collected from any five spots series, reaching for each aliquot a total of 2000 × 5 shots. The analytic variability was estimated for each feature using ANOVA [17].

### Within- and between-subjects variability of urinary MALDI-TOF/MS-based peptidome

Within- and between-subjects variability of MALDI-TOF/MS-based urinary peptidome features were evaluated using random urine samples collected from twenty healthy males (age range = 24–56 years). From 16 subjects two urine samples were collected over a 5 days period, while form the remaining 4 subjects three urine samples were collected over an 8 day-period. After centrifugation at 16,000*g* for 15 min to eliminate cell debris, each urine sample was split in three 2 mL aliquots, two of which were immediately frozen at − 80 °C, while one aliquot was dialyzed and then stored at − 80 °C for less than 3 months.

The dialyzed aliquots obtained from each subject were thawed, processed, and spotted in quintuplicate in a MALDI-TOF/MS plate in the same analytical session. A total of 2000 shots were collected for each sample. After spectra processing, the ANOVA method was used to calculate within- and between-subject variability and the intraclass correlation coefficient (ICC).

### Serum preparation for MALDI-TOF/MS analyses

One hundred μL of serum were mixed with 100 μL of acetonitrile (ACN). The mixture was incubated at room temperature for 30 min, during which it was vigorously vortexed three times, causing abundant proteins to precipitate and leaving LMW peptides free in the supernatant. The samples were then centrifuged for 10 min at 25,000*g*, the supernatant was collected, evaporated under a gentle flux of nitrogen and re-suspended in 50 μL of 0.1% trifluoroacetic acid (TFA). The samples were stored at − 80 °C for no more than 1 week before MALDI-TOF/MS analysis. The average amount of ACN precipitated proteins was 7 mg (average protein concentration of 70.2 g/L measured using two serum samples, Bio-Rad protein assay). TFA re-suspended samples had a mean protein concentration of 1.1 μg/μL.

### Urine preparation for MALDI-TOF/MS analyses

Urine dialysis was performed before MALDI-TOF/MS analysis, using the Spectra/Por 7 semi-permeable membrane and a molecular weight cut-off of 1 kDa, following the manufacturer’s protocol (Spectrum laboratories, CA, USA).

### Serum and urine MALDI-TOF/MS analysis

10 μL, equivalent to 11 μg proteins, of desalted serum (Merck Millipore ZipTip, Milano, Italy) and 10 μL of dialyzed urine were mixed with 10 μL of saturated HCCA (α-cyano-4-hydroxycinnamic acid) prepared in 0.1% trifluoroacetic acid (TFA) and acetonitrinile (ACN) (2:1 v/v). 1 μL of this mixture was spotted on a ground steel MALDI-TOF/MS target. Crystallization was always performed at constant humidity and temperature ranges during all the experimental sessions. MALDI-TOF/MS measurements were taken using an Ultraflex II instrument (Bruker Daltonics, Bremen, Germany) operating in conditions described elsewhere [18]. The mass to charge (m/z) ratio ranging from 1000 to 4000 was analysed. Spectra processing was performed as described in detail in the Additional file 1: Materials and methods section.

### MALDI-TOF/MS signal limit of detection (sLOD)

The sLOD for serum MALDI-TOF/MS was determined as previously described by us [12], and detailed in Additional file 1: Materials and methods section.

### Monte Carlo simulations estimating the reliability of the Intraclass correlation coefficient (ICC) in datasets that include measurement errors and LOD issues

In accordance with Carroll’s work, being the data from MALDI-TOF/MS profiling log-normal distributed, the multiplicative measurement error model was used as more appropriated [13]. Monte Carlo simulation methods were used to test the hypothesis that ICC is a reliable estimator of the measurement error even in the presence of LOD issues. The procedure is detailed in the Additional file 1: Materials and methods section.

### MALDI-TOF/MS features with sLOD adjustment, data median normalization and log_2_ transformation

The sLOD adjustment procedure, the data median normalization and log_2_ transformation methods are outlined in the Additional file 1: Materials and methods section.

### PCa study

Random urine samples were collected from 148 patients referring LUTs to specialists staffing the Urological Unit of the University-Hospital of Padova. All of the patients underwent a complete physical examination including a digital rectal examination (DRE) after urine collection. A fasting serum sample was also collected prior to any prostatic manipulations. All the patients also underwent a transrectal ultrasound biopsy of the prostate with a 10- to 16-core template. The patients’ histological diagnoses were retrieved from their medical records. The Gleason score was available for 64 of the patients. The urine and serum samples were frozen within 3 h of being collected and stored at − 80 °C for less than 3 months before undergoing MALDI-TOF/MS analyses. MALDI-TOS/MS analyses were performed by randomly thawing and processing a batch of 20 samples/run. The random selection of the samples collected for MALDI-TOF/MS analyses made it possible to minimize the time-related peptidomic differences that might occur due to different storage times.

### Biomarkers measurements

Serum total-PSA (tPSA) and free-PSA (fPSA) were measured using the Immulite^®^ 2000 system (Siemens Healthcare Diagnostics srl, Siemens, Milan, Italy). For PCA3 analyses, urine samples were collected after digital rectal examination (DRE) using a Progensa™ PCA3 (Gen-Probe, San Diego, CA, USA) assay following the manufacturer’s instructions. PCA3 mRNA and PSA mRNA values were determined. The PCA3 score was determined by calculating the PCA3 mRNA/PSA mRNA × 1000 ratio. Urinary creatinine was measured using a Cobas 6000 (c501) analyser (Roche Diagnostics S.p.a., Monza, Italy).

### MALDI-TOF/MS–MS peptide fragmentation

10 μL of serum samples was initially desalted by ZipTip and then 5 μL was mixed with 5 μL of saturated HCCA (α-cyano-4-hydroxycinnamic acid) prepared in 0.1% trifluoroacetic acid (TFA) and acetonitrinile (ACN) (1:1 v/v). One μL of this mixture was spotted two times on a ground steel MALDI-TOF/MS target. MALDI-TOF/MS–MS measurements were performed on a 4800 Plus MALDI TOF/TOF Analizer (AB Sciex). For MS spectra the instrument operated in reflector mode with an acceleration voltage of 20 kV, a grid voltage of 16 kV, and a delay extraction of 450 ns. 1500 shots were averaged using a laser energy of 3500 (arbitrary units). Ions selected for to MS/MS analysis were subjected to CID fragmentation loading air in the collision cell of the instrument. 3500 shots were averaged for each MS/MS using a laser energy of 4500 (arbitrary units) and setting 8 kV for source 1 and 15 kV for source 2.

### RCAL and SIMEX for logistic regression analyses

After the ICC was estimated for each MALDI-TOF/MS feature, the intensity values of the urine and serum samples were determined, and an unbiased βˆ* coefficient estimation was calculated by dividing the βˆ naïve coefficients (obtained from the naïve logistic regression) with the corresponding ICC (βˆ* = βˆ/ICC). βˆ* confidence intervals were calculated in the manner suggested by Rosner [19]. SIMEX estimates were calculated using the Cook and Stefanski’s method [20]. Further details are available in the Additional file 1: Materials and methods section.

### Statistical softwares

All analyses were performed using the R statistical software, including the SIMEX and “ICC” packages and Stata v13.1 (StataCorp, Lakeway Drive, TX, USA). A detailed description of the R packages used are reported in Additional file 2: Intermediate results.

## Results

### Analytical intra- and inter-assay variability of MALDI-TOF/MS serum peptidome features

As described in the “Methods” section, the intra- and inter-assay variability of serum MALDI-TOF/MS peptidome features were evaluated using pooled sera. After MALDI-TOF/MS spectra processing during which a m/z ranging from 1000 to 4000 Da was considered, 14 shared features were found in all the spectra. The intra- and inter-run analytical variability of MALDI-TOF/MS serum profiling was calculated for: (a) non-normalized data, (b) median normalized data and (c) median normalized sLOD adjusted data, and the results are outlined in Table 1. Median intra- and inter-assay variabilities of non-normalized and median normalized serum features were comparable to those we previously obtained from non-normalized signals in urine samples (reported elsewhere) [12].

**Table 1:** Intra- and inter-assay variability of serum MALDI-TOF/MS peptidomic patterns, expressed as the median coefficient of variation (CV)

Normalization stategies	Intra-assay CVs Median (IQR)	Inter-assay CVs Median (IQR)
Non-normalized features	38.16 (33.44–44.50)	64.12 (53.95–72.23)
Median normalized features	24.89 (21.84–29.29)	35.72 (30.93–47.27)
Median normalized and sLOD adjusted features	30.10 (27.37–33.11)	40.80 (34.64–50.17)

The results of different normalization strategies are shows

*sLOD* signal limit of detection, *IQR* interquartile range

### Urinary creatinine measurements and within- and between-subjects variability of MALDI-TOF/MS urinary peptidomic features

The ICC for urinary creatinine was 0.40 (95% CI 0.03–0.69) and the between and within subject variances were 13.45 and 9.01 mmol^2^/L^2^, respectively. As the ICC is one of the most commonly used reliability indices for repeated measurements, the low value obtained underlined the wide variation in urinary creatinine that can exist over a single day in the same subject (ICC may vary between 0 and 1).

Urine samples collected for within- and between-subject variability underwent MALDI-TOF/MS analysis, and a total of 171 shared features were discovered. ICC was calculated for each feature and then evaluated together. As the ICC distributions were highly skewed (Shapiro–Wilk test, *p* < 0.001), median and interquartile ranges (IQR) were used as descriptive statistics. With respect to the non-normalized features, median normalized and creatinine normalized features had lower but similar ICCs. The ICCs calculated for features after the sLOD adjustment, median normalization and log_2_ transformation (sLMNLT) were similar to (but slightly higher than) those of the median or creatinine normalized signals (Table 2). Notably, the ICCs produced by MALDI-TOF/MS features were comparable to those of urinary creatinine, underlining the fact that the observed elevated intra-individual variability mostly reflects day-to-day biological variations in individual patients. Moreover, the ICC features were not significantly correlated with the m/z features, neither in the median normalized nor in the sLMNLT features (Spearman’s ρ = − 0.01, *p* = 0.922; Spearman’s ρ = − 0.04, *p* = 0.626, respectively). As the ICC of the urine sLMNLT features was the highest obtainable, this normalization strategy was used for the following analyses.

**Table 2:** Intraclass correlation coefficient (ICC) and within- and between-subjects variability of urinary MALDI-TOF/MS peptidomic profiling, calculated using different normalization strategies

	ICC (median and IQR)	Within subjects variability (median and IQR)	Between-subjects variability (median and IQR)
Non-normalized features	0.36 (0.24–0.47)	1266 (606–411,000)	739 (198–420,600)
Median normalized features	0.45 (0.26–0.62)	0.140 (0.03–2.38)	0.158 (0.033–3.103)
Creatinine normalized features	0.45 (0.32–0.60)	26.9 (6.5–325.5)	34.6 (6.40–264.12)
sLOD adjusted, Median normalized, log_2_ transformed features (sLMNLT features)	0.48 (0.32–0.65)	0.184 (0.12–0.33)	0.225 (0.08–0.43)
sLOD adjusted, Creatinine normalized, log_2_ transformed features	0.35 (0.24–0.47)	0.479 (0.350–0.687)	0.422 (0.335–0.619)

Median values and interquartile ranges (IQR) are outlined

### Within- and between-subjects variability of serum MALDI-TOF/MS peptidomic profiling

After spectral alignment, a total of 20 shared peptidomic features were detected in the 42 serum samples analyzed. Within- and between-subjects variances were calculated for the non-normalized, median normalized and sLMNLT features signals and the results are outlined in Table 3. The median ICCs were higher than those calculated for the urinary samples; and normalization did not significantly modify MALDI-TOF/MS serum peptidomic ICCs. The ICC values were correlated with the m/z features of the non-normalized data (ρ = 0.49, *p* = 0.03) but were not correlated with the median normalized or the sLMNLT features (Spearman’s ρ = 0.36, *p* = 0.11 and Spearman’s ρ = 0.39, *p* = 0.88, respectively). As the ICCs of the serum sLMNLT features were the highest that were obtained, that normalization strategy was used for the following analyses.

**Table 3:** Intraclass correlation coefficient (ICC) and within- and between-subjects variability, of serum MALDI-TOF/MS peptidomic profiling calculated using different normalization strategies

	ICC (median and IQR)	Within subjects variability (median and IQR)	Between-subjects variability (median and IQR)
Non-normalized features	0.58 (0.40–0.73)	614.4 (192.2–19,560.0)	771.6 (218.9–14,300.0)
Median normalized features	0.62 (0.49–0.73)	0.04 (0.01–0.76)	0.05 (0.01–1.02)
sLMNLT features	0.64 (0.49–0.72)	0.76 (0.27–11.1)	0.86 (0.27–18.6)

Median values and interquartile ranges (IQR) are outlined

### Monte Carlo simulations confirmed that substituting below LOD values by LOD/2 does not affect ICC estimation reliability

A series of Monte Carlo simulations were performed with the intent of verifying if LOD issues affect the ICC calculation, in particular when the strategy of substituting LOD/2 for values below the LOD is used. Overall, the results obtained using simulations (Additional file 3: Table S1 and Additional file 4: Figure S1) showed that when the percentage of the values below LOD is less than 50%, substituting the corresponding LOD/2 calculated values for the values below LOD did not affect ICC estimation reliability. Further details regarding the simulations are outlined in the Additional file 5: Results.

### The measurement error of the MALDI-TOF/MS urinary and serum peptidomic features

The measurement error was estimated independently for the MALDI-TOF/MS urinary and serum peptidomic features as explained above. Satisfactory results were obtained when the two measurement error structures were verified for constant variance and symmetry, thus suggesting the usage of these data for MALDI-TOF/MS features results. Further details are available in the Additional file 5: Results, Additional file 6: Figure S2 and Additional file 7: Figure S3.

### PCa study

Prostate histology showed no alterations in 57 of the patients participating in the study; PCa was documented in 55 patients while and the remaining 36 had other prostate alterations (Table 4). The subjects were thus reclassified based on the presence or absence of histologically confirmed prostate cancer (Reference, n = 93 contains non PCa patients). The patients’ fPSA, tPSA, f/tPSA and PCA3 scores were not normally distributed (Shapiro–Wilk test: *p* < 0.001 for all) and evaluated by non-parametric statistics. Patients’ ages and tPSA were not associated with the presence or absence of PCa (*t* test: t = − 1.38, *p* = 0.168 and Kruskal–Wallis: Χ^2^ = 1.85, *p* = 0.179, respectively). Instead, the fPSA (Χ^2^ = 5.82, *p* = 0.015) and f/tPSA (Χ^2^ = 22.45, *p* < 0.001) were significantly associated with the presence of PCa. ROC curve analyses further supported that f/tPSA area under the curve (AUC 0.729; 95% CI 0.643–0.813) is more sensitive than tPSA (AUC 0.565; 95% CI 0.468–0.661) and fPSA (AUC 0.617; 95% CI 0.522–0.710) in discriminating between the presence or the absence of PCa in patients with LUTS (*p* < 0.001 for all the pairwise comparisons). In particular, when a cut-off of 10% for f/tPSA was used the sensitivity, specificity, positive and negative likelihood ratio were 67.7% (95% CI 54.5–78.7%), 71.7% (95% CI 60.1–80.7%), 2.40 (95% CI 1.64–3.50) and 0.45 (95% CI 0.31–0.65), respectively. ROC analyses of the PCA3 score showed an AUC of 0.664 (95% CI 0.564–0.764), and when a cut-off of 35 was used, the sensitivity, specificity, and positive and negative likelihood ratios were 69.1% (95% CI 55.2–80.9%), 48.4% (95% CI 37.9–59.0%), 1.34 (95% CI 1.03–1.74) and 0.64 (95% CI 0.41–0.99), respectively.

**Table 4:** Summary statistics of age, free prostate specific antigen (fPSA), total PSA (tPSA), free to total PSA (f/tPSA) and prostate cancer antigen 3 (PCA3) of the subjects included in the PCa study, grouped by the prostate biopsy histological results

	N (%)	Age mean ± SD	fPSA (μg/L) Median (IQR) (n = 142)	tPSA (μg/L) Median (IQR) (n = 142)	f/tPSA (μg/L) Median (IQR) (n = 142)	PCA3 score Median (IQR) (n = 142)
No alterations	57 (38.5%)	65.3 ± 6.7	0.74 (0.43–1.36)	5.05 (3.6–7.75)	15.7 (10.10–19.40)	23.50 (13.00–46.50)
BPH	7 (4.7%)	65.8 ± 8.3	1.50 (0.9–2.17)	7.01 (4.46–14.6)	17.00 (14.90–19.00)	49.00 (44.50–82.50)
Inflammation	12 (8.1%)	66.2 ± 4.3	0.47 (0.26––0.93)	4.00 (2.17–8.04)	11.00 (9.15–16.20)	22.00 (19.00–48.00)
AAH	2 (1.4%)	61.5 ± 0.7	0.47 (0.26–1.00)	7.13 (4.97–9.3)	6.45 (4.70–8.20)	21.50 (20.25–22.75)
ASAP	11 (7.4%)	64.7 ± 7.8	0.71 (0.48–1.10)	7.88 (3.85–9.16)	11.40 (9.60–15.70)	19.5 (14.5–32.00)
HGPIN	4 (2.7%)	69.0 ± 5.0	0.49 (0.25–1.35)	5.07 (4.11–5.83)	10.50 (6.30–22.40)	41.00 (26.00–70.00)
PCa	55 (37.2%)	66.8 ± 7.1	0.43 (0.27–0.83)	6.54 (4.08–9.23)	8.10 (5.30–11.13)	50.00 (24.00–88.00)

No alteration, no histological alteration; BPH, benign prostatic hyperplasia; Inflammation, chronic inflammation; AAH, atypical adenomatous hyperplasia; ASAP, atypical small acinar proliferation; HGPIN, high-grade prostatic intraepithelial neoplasia; PCa, prostatic neoplasia

**Fig. 2:**
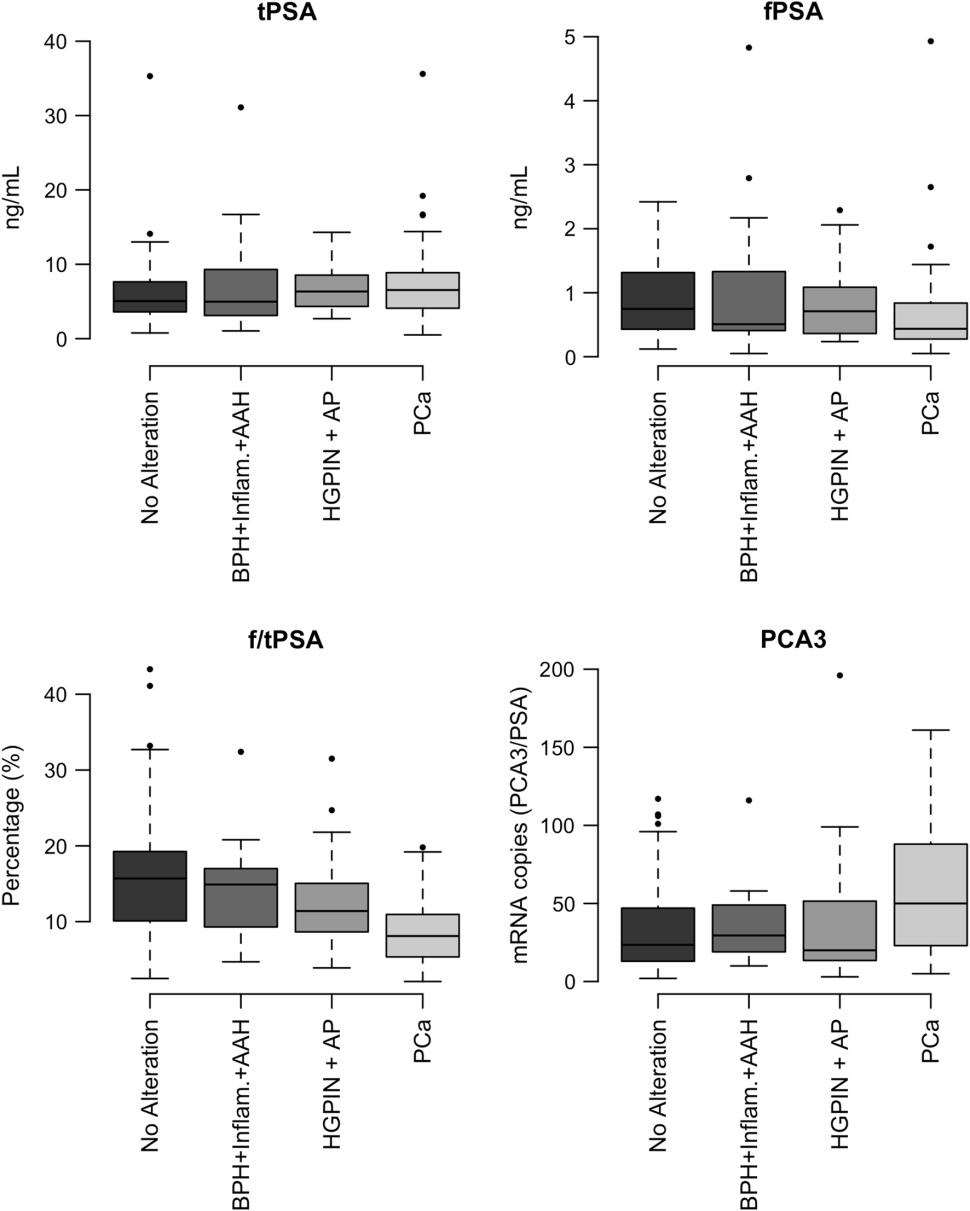
Tukey’s box plot of free prostate specific antigen (fPSA), total PSA (tPSA), free to total PSA (f/tPSA) and prostate cancer antigen 3 (PCA3) of the subjects included in the PCa study, grouped by the histological-based reclassification described in results (*BPH* benign prostatic hyperplasia, *Inflam.* chronic inflammation, *AAH* atypical adenomatous hyperplasia, *ASAP* atypical small acinar proliferation, *HGPIN* high-grade prostatic intraepithelial neoplasia, *PCa* prostatic neoplasia)

The patients were further re-classified into four groups on the basis of their histological results: Group (A) No Alteration (n = 57); Group (B) benign prostatic hyperplasia (BPH) and inflammation (n = 19); Group (C) high-grade prostatic intraepithelial neoplasia (HGPIN) and atypical small acinar proliferation (ASAP) (n = 15); and Group (D) PCa (n = 55). The distributions of the tPSA, fPSA f/tPSA and PCA3 values were then evaluated and the results are outlined in Additional file 8: Table S2 and Fig. 2. No statistically significant results were found for tPSA and fPSA. PCA3 levels were higher in group D with respect to group A (Tukey’s adjusted *p* value for multiple testing: *p* = 0.023), and f/tPSA was lower in group D with respect to both group A (*p* = 0.023) and B (*p* = 0.028); other significant differences were also found for f/tPSA between groups C and D (*p* = 0.018).

After MALDI-TOF/MS spectra evaluation, considering a m/z ranging from 1000 to 4000 Da, a total of 482 features were identified in urine and 186 in sera. The MALDI-TOF/MS peptidomic content of serum and urine was then compared. Considering a window of m/z of 0.3 Da, forty-three features (8.9 and 29.1% of the total urinary and serum features) were commonly identified in both matrixes and the results are outlined in Additional file 9: Table S3.

Naïve logistic regression analysis was chosen to identify PCa-associated features. The Reference group, made of patients with no alterations, BPH and inflammation, was compared with the cancer lesions group, which included patients with PCa, HGPIN and ASAP. A total of 11 urinary features that were significantly associated with cancer were identified. The RCAL and SIMEX were consequently utilized for estimating, in addition to naïve logistic regression (biased), the unbiased regression coefficients. Urinary peptidomic features at 1404.6 and 1755.7 m/z had an estimated within-subject variability (error structure) that could be used by RCAL to estimate β* (ICC were 0.459 and 0.455, respectively). For the 9 remaining features, for which the error structures were not calculable, the median ICC or, alternatively, the median within-subject variance was utilized for RCAL or SIMEX calculation, respectively (ICC = 0.458 and σ_w_^2^ =  0.184). The results of naïve β logistic regression coefficient, RCAL and SIMEX β* logistic regression coefficients (including the Odds ratio) and the Wald test p-value are outlined in Table 5. Overall, those results demonstrate that 8 out of 11 urinary features presented diminished signals, while only 2 had increased signals in the cancer lesions patients with respect to the reference group. After multiple-testing correction using the Benjamini-Hochberg (BH) method, no significant *p* values were obtained. Moreover, at multivariate logistic regression analysis, which included all 11 significant features, no feature remained statistically significant.

**Table 5:** Naïve and RCAL logistic regression results for urinary MALDI-TOF/MS features

m/z	Naïve logistic regression			RCAL			SIMEX		
	β	OR	Wald test *p* value	β*	β* 95% CI	OR	β*	β* 95% CI	OR
1399.8	0.324	1.38	0.032	0.707	0.06–1.35	2.03	0.342	0.03–0.651	1.41
1404.6	0.435	1.54	0.012	0.947	0.21–1.69	2.57	0.459	0.11–0.81	1.58
1553.8	− 0.517	0.60	0.038	− 1.129	− 2.20 to − 0.06	0.32	− 0.600	− 1.13 to − 0.04	0.55
1556.0	− 0.757	0.47	0.018	− 1.652	− 3.03 to − 0.27	0.19	− 0.868	− 1.58 to − 0.12	0.42
1688.0	− 0.710	0.49	0.014	− 1.549	− 2.79 to − 0.30	0.21	− 0.833	− 1.48 to − 0.16	0.43
1707.1	− 0.928	0.40	0.008	− 2.025	− 3.53 to − 0.51	0.13	− 1.051	− 1.85 to − 0.25	0.35
1755.7	− 0.269	0.76	0.018	− 0.591	− 1.08 to − 0.10	0.55	− 0.270	− 0.50 to − 0.05	0.76
1782.1	− 0.767	0.46	0.040	− 1.674	− 3.28 to − 0.06	0.19	− 0.977	− 1.79 to − 0.01	0.38
2416.3	− 0.552	0.58	0.045	− 1.205	− 2.38 to − 0.02	0.30	− 0.565	− 1.20 to 0.01	0.57
2594.3	− 0.543	0.58	0.029	− 1.184	− 2.25 to − 0.12	0.31	− 0.601	− 1.14 to − 0.08	0.55
2797.8	− 0.516	0.60	0.042	− 1.126	− 2.21 to − 0.04	0.32	− 0.550	− 1.06 to − 0.01	0.58

The results of RCAL were calculated using the median ICC; the SIMEX results were calculated using the within-subject σ^2^. Beta coefficients represent the logistic regression coefficient results, while the β* represent the RCAL and SIMEX results. These two models were applied in order to obtain the unbiased regression coefficients β*

OR odds ratio, *RCAL* regression calibration analysis, *SIMEX* simulation and extrapolation analysis

Wald test *p* value = univariate statistics obtained from the logistic regression, without adjusting *p* values for multiple comparison

*RCAL or SIMEX adjusted logistic regression coefficient

Thirty-four features of serum peptidome were significantly associated with cancer lesions after Benjamini–Hochberg (BH) multiple-testing, as 10 out of 34 features were increased in the patients with cancer lesions. For the m/z features at 1020.5 and 1418.6, there were estimable error structures (the ICCs were 0.617, 0.507, while σ_w_^2^ s were 16.74, 0.776). For the 31 remaining features, the error structures were not estimable and the median ICC or the median within-subject variances were utilized for RCAL or SIMEX calculation, respectively (ICC = 0.616 and σ_w_^2^ =  0.766); the results are outlined in Table 6. According to multivariate analysis, when all 34 statistically significant features were included, only the m/z feature at 1405.75 remained statistically significant (*p* = 0.020). The raw data, and intermediate files are available as Additional file 10: Data file, Additional file 11: Raw data 2, Additional file 12: Raw data 3, and Additional file 2: Intermediate results.

**Table 6:** Naïve logistic regression, RCAL and SIMEX of serum MALDI-TOF/MS features found to be statistically significant (*p* < 0.05) following the Benjamini–Hochberg (BH) procedure

Features’ m/z	Naïve logistic regression			RCAL			SIMEX		
	β	OR	BH adjusted p-value	β*	β* 95% CI	OR	β*	β* 95% CI	OR
1020.5	0.32	1.37	0.032	0.52	0.16–0.87	1.68	0.44	0.17–0.71	1.55
1192.4	− 0.79	0.45	0.035	− 1.29	− 2.20 to − 0.38	0.28	− 1.56	− 2.45 to − 0.67	0.21
1218.6	− 0.95	0.39	0.017	− 1.54	− 2.46 to − 0.62	0.21	− 1.90	− 2.80 to − 0.99	0.15
1367.8	− 0.67	0.51	0.017	− 1.08	− 1.69 to − 0.47	0.34	− 1.01	− 1.56 to − 0.47	0.36
1405.7	0.69	2.00	0.023	1.13	0.39–1.86	3.08	1.24	0.54–1.94	3.46
1418.6	0.32	1.38	0.017	0.52	0.20–0.84	1.68	0.38	0.14–0.62	1.46
1418.6	0.32	1.38	0.017	0.63	0.24–1.01	1.87	0.38	0.13–0.61	1.46
1440.6	1.03	2.80	0.028	1.67	0.54–2.80	5.30	2.24	1.11–3.38	9.44
1460.7	0.62	1.85	0.043	1.00	0.26–1.74	2.72	1.10	0.38–1.81	2.99
1504.9	− 0.41	0.66	0.023	− 0.67	− 1.10 to − 0.23	0.51	− 0.55	− 0.92 to − 0.18	0.58
1591.1	− 0.65	0.52	0.017	− 1.06	− 1.68 to − 0.44	0.35	− 1.10	− 1.66 to − 0.54	0.33
1605.9	− 0.20	0.82	0.043	− 0.32	− 0.55 to − 0.08	0.73	− 0.21	− 0.37 to − 0.05	0.81
1719.0	− 0.49	0.61	0.021	− 0.79	− 1.30 − 0.28	0.45	− 0.69	− 1.13 to − 0.25	0.50
1739.9	0.26	1.30	0.017	0.42	0.17–0.67	1.52	0.28	0.11–0.46	1.33
1818.9	− 1.21	0.30	0.014	− 1.96	− 3.02 to − 0.89	0.14	− 2.53	− 3.58 to − 1.48	0.08
1826.7	− 0.82	0.44	0.014	− 1.34	− 2.05 to − 0.63	0.26	− 1.37	− 2.04 to − 0.70	0.25
1832.1	− 1.66	0.19	0.003	− 2.69	− 3.9 to − 1.47	0.07	− 3.52	− 4.79 to − 2.25	0.03
1835.0	− 0.72	0.49	0.017	− 1.17	− 1.86 to − 0.46	0.31	− 1.25	− 1.92 to − 0.58	0.29
1838.9	− 0.50	0.61	0.036	− 0.80	− 1.37 to − 0.23	0.45	− 0.74	− 1.25 to − 0.23	0.48
1847.0	− 0.37	0.69	0.017	− 0.60	− 0.96 to − 0.24	0.55	− 0.44	− 0.71 to − 0.17	0.64
1886.0	− 0.79	0.45	0.024	− 1.29	− 2.14 to − 0.44	0.28	− 1.43	− 2.27 to − 0.59	0.24
1895.9	0.30	1.35	0.017	0.48	0.20–0.76	1.62	0.34	0.14–0.54	1.40
1902.9	− 0.98	0.38	0.028	− 1.59	− 2.66 to − 0.51	0.20	− 2.12	− 3.21 to − 1.04	0.12
1919.2	− 0.73	0.48	0.023	− 1.18	− 1.95 to − 0.41	0.31	− 1.24	− 1.97 to − 0.51	0.29
1934.1	− 0.32	0.72	0.017	− 0.53	− 0.83 to − 0.22	0.59	− 0.37	− 0.59 to − 0.15	0.69
1968.9	− 1.03	0.36	0.017	− 1.67	− 2.71 to − 0.62	0.19	− 2.08	− 3.15 to − 1.02	0.12
1977.1	− 0.96	0.38	0.017	− 1.55	− 2.51 to − 0.59	0.21	− 1.90	− 2.87 to − 0.93	0.15
1980.2	− 1.01	0.36	0.017	− 1.65	− 2.67 to − 0.62	0.19	− 2.14	− 3.16 to − 1.11	0.12
1984.5	− 0.64	0.53	0.017	− 1.04	− 1.63 to − 0.44	0.35	− 0.96	− 1.51 to − 0.41	0.38
1994.9	− 1.53	0.22	0.012	− 2.48	− 3.75 to − 1.20	0.08	− 3.29	− 4.61 to − 1.97	0.04
2006.3	− 0.26	0.77	0.017	− 0.43	− 0.70 to − 0.16	0.65	− 0.30	− 0.49 to − 0.10	0.74
2037.1	− 0.82	0.44	0.032	− 1.33	− 2.25 to − 0.40	0.27	− 1.60	− 2.49 to − 0.71	0.20
3156.6	0.33	1.39	0.043	0.54	0.14–0.93	1.71	0.45	0.13–0.76	1.56
3272.6	0.52	1.69	0.029	0.85	0.27–1.43	2.34	0.82	0.29–1.34	2.27
3681.0	0.59	1.80	0.043	0.95	0.25–1.65	2.59	1.06	0.39–1.73	2.89

The results of RCAL were calculated using the median ICC; the SIMEX results were calculated using the within-subject σ^2^. RCAL and SIMEX models were applied in order to obtain the unbiased regression coefficients β*

*OR* odds ratio, *RCAL* regression calibration analysis, *SIMEX* simulation and extrapolation analysis, *BH* Benjamini–Hochberg

*RCAL or SIMEX adjusted logistic regression coefficients

**Fig. 3:**
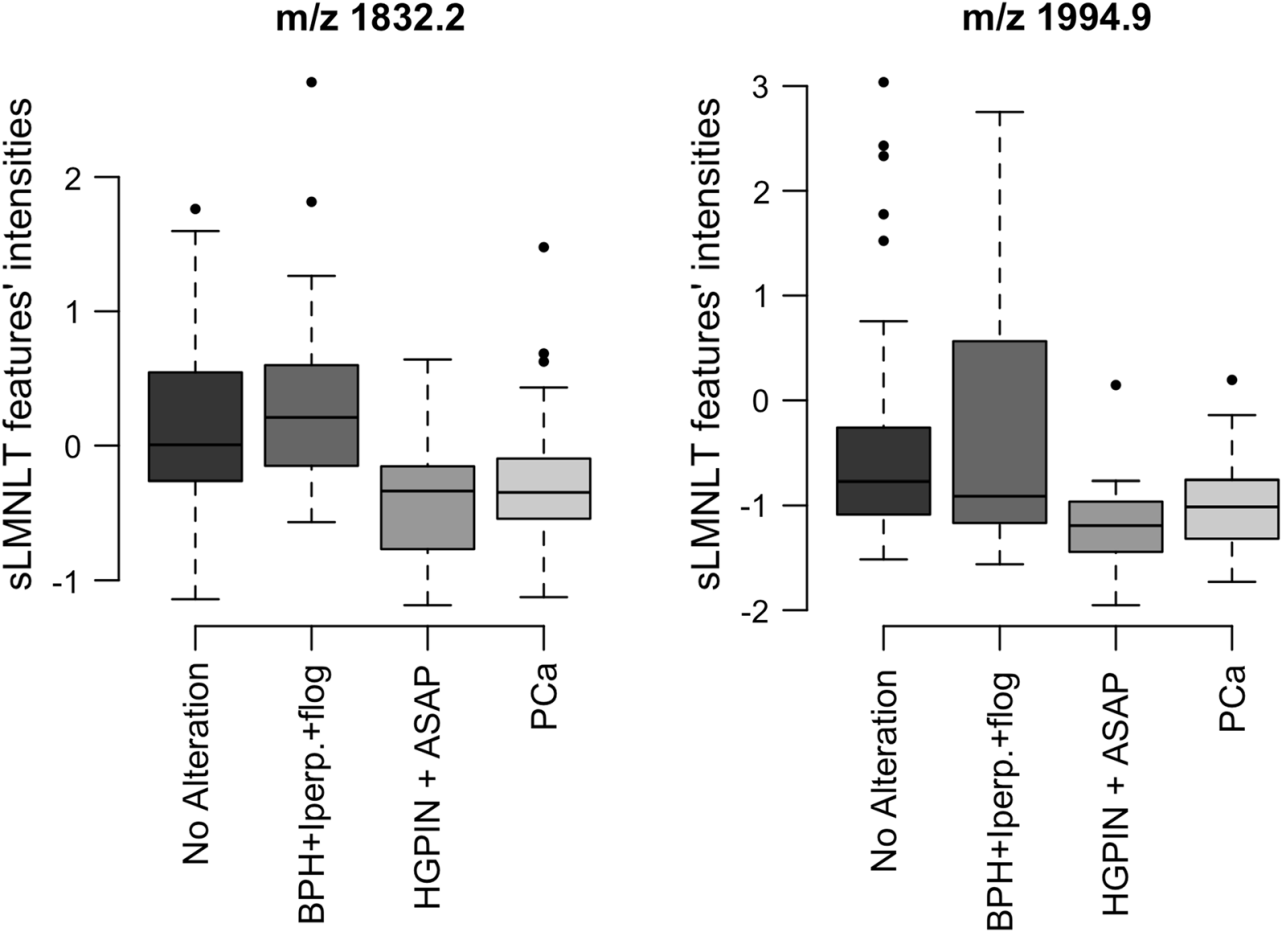
Tukey’s box plot of sLOD adjusted, median normaled and log_2_ transformed (sLMNLT) intensities of MALDI-TOF/MS features at m/z 1832.2 and 1994.9, grouped by the histological-based reclassification described in “Results” section (*BPH* benign prostatic hyperplasia, *Inflam.* chronic inflammation, *AAH* atypical adenomatous hyperplasia, *ASAP* atypical small acinar proliferation, *HGPIN* high-grade prostatic intraepithelial neoplasia, *PCa* prostatic neoplasia)

Analyses were performed depending on the histological-based reclassification (groups A, B, C and D). None of the 482 urinary peptidomic features were statistically significantly associated with any of the four groups. Instead, among the 186 serum features, those at m/z 1832.8 and 1994.9 resulted statistically significantly associated with cancer lesions after BH multiple-testing adjustment (*p* = 0.002 and *p* = 0.019, respectively) (Fig. 3). In particular, the feature at m/z 1832.2 was able to discriminate PCa (group D) or HGPIN and ASAP (group C) versus patients with no alteration (group A) and BPH (group B) (*p* < 0.001), while the one at m/z 1994.9 was unable to distinguish BPH patients from PCa ones (*p* = 0.394), but it was nevertheless able to distinguish group A from groups C and D (*p* < 0.001).

At this point, the Gleason score, which was available for only some of the PCa patients, was analysed. When scores of <= 7 (n = 45) or > 7 (n = 9) were used to stratify the patients, neither MALDI-TOF/MS features (serum or urine) nor tPSA, fPSA and f/tPSA or PCA3 values were found to be statistically significant predictors of PCa grading at logistic regression.

All the MALDI-TOF/MS sera peptidomic features that were statistically significantly associated with PCa at univariate and multivariate analyses were further characterized by MALDI-TOF/MS–MS, and the results were evaluated using the MS-Tag bioinformatics tool of ProteinProspector v 5.19.1 (available at: http://prospector.ucsf.edu/prospector/mshome.htm) in the attempt to identify the candidate protein (full molecule) derived from peptide fragmentation. As outlined in Additional file 13: Table S4, there was a significant fragmentation pattern for 12 features allowing us to identify the corresponding proteins. The raw data of MALDI-TOF/MS–MS fragmentation spectra of the significant 12 features are provided as Additional file 14: MS-Tag results.

## Discussion

Early stage PCa is often painless and indolent, but, as the tumor advances, symptoms may become less vague and more similar to those caused by LUTs. The European Association of Urology (EAU) guidelines for the management of patients with LUTs recommend both DRE and PSA testing, in particular when a diagnosis of PCa would change the patient’s management [1]. In view of these considerations, we tested the sensitivity of some well-established serum biomarkers for PCa in 148 patients who were referred to urologists for LUTS. Overall, the study showed that both tPSA and fPSA have poor sensitivity in discriminating between PCa and non-PCa in patients presenting LUTs. The f/tPSA ratio showed an AUC of 0.729, but the positive and negative predictive values were low, limiting the clinical applicability of this PSA-based tumour marker. Prostate DRE stimulates the release of gland fluids, tumour cells, tumour enriched nucleic acid and peptides/proteins into the urine, and PCA3, calculated as the ratio of two mRNAs (PCA3/PSA) released into the urine after DRE, seems to be useful in predicting the diagnosis of PCa at the first or second biopsy [5, 21]. Our results showed, however, that this marker shared with f/tPSA a good sensitivity (67.7% for f/tPSA and 69.1% for PCA3), but with respect to f/tPSA it is less specific for LUTS patients (71.7% for f/tPSA and 48.4% for PCA3). Given these considerations, new PCa biomarkers would be a timely discovery.

To discover single or panels of biomarkers proteomic techniques appear the most reliable, and these include LC–MS/MS and MALDI-TOF/MS [22]. Although the former has significant advantages for proteomic profiling with respect to the latter technique, we choose MALDI-TOF/MS because it is fast and it has less requirements for sample preparation, these characteristics fitting well with the high number of samples used in this study. However, when using MALDI-TOF/MS, the reproducibility issues may hinder its applicability. We found in a previous study that the analytical variability of urine peptidomic profiling was high, but we also demonstrated that combining bioinformatics strategies (to handle data normalization) and new approaches to the problem of sLOD can reduce features’ variability, improving the reproducibility of results [12]. The current study, which evaluated the reliability of MALDI-TOF/MS serum profiling, found that median normalization of data can effectively reduce its intra- and inter-assay variability. Our attempt to substitute sLOD/2 for values below sLOD did not result as efficient in serum as it did in urine in enhancing MALDI-TOF/MS reproducibility. In fact, when sLOD adjustment was used together with median normalization, the median coefficients of variation were not reduced. The differences in the serum vs urine results can be explained by spectra noise patterns, which are usually higher in urine than in serum (data not shown).

Despite the features’ data normalizations, MALDI-TOF/MS peptidomic variability of both urine and serum was higher than 20%, thus analytical variability was clearly an important source of measurement error that had to be considered during analyses. Modelling data with measurement error during statistical analysis often leads to an underestimation of the association between biomarker levels and the disease. Although the error structures need to be known in advance, RCAL and SIMEX make it possible to account for measurement error by statistics. In accordance with measurement error theory, the ICCs were estimated for MALDI-TOF/MS peptidomic features by the within- and between-subjects variability [13]. These values were obtained using two separate datasets, one for the urine and the other for the serum samples. The samples repeatedly collected from the same subject over a short time period are used to estimate the within-subject variability; the between-subjects variability being defined as the differences among subjects. The ICCs, used as calibration coefficient by the RCAL, are normally estimated for data without measurement error or LOD issues. Therefore, the usefulness of ICC for MALDI-TOF/MS data in dealing with measurement error issues first needed to be experimentally validated. A series of Monte Carlo simulations were performed, varying simultaneously the amount of measurement error and the percentage of values below LOD. The simulation results were then compared with those obtained using other methods for handling data missing due to LOD issues, namely the Richardson and Ciampi’s and Schisterman’s methods [15, 16]. The results showed that, as expected, ICC decreased from 1 to approximately 0.35 when the measurement error variance ranged from 0 to 0.64. Interestingly, in the same conditions, Richardson and Ciampi’s strategy outperformed all the other methods tested; however, substituting sLOD/2 for values below the LOD produced similar results if the percentage of values below LOD was less than 25%. Overall, our results indicated that ICCs were not over- or underestimating measurement error in those cases in which the LOD issue was correctly handled.

The ICC median values of sLMNLT MALDI-TOF/MS peptidomic features were 0.48 for urine and 0.64 for serum. These results showed that within-subject urine peptidomic variations were greater than those found in the serum. This result was expected as within-subject urine variation may depend not only on an individual’s physiological status but also on several other factors, such as hydration and diet. In agreement, urinary creatinine showed high variability in serial samples of urine collected from the same subject. Furthermore, our results are in agreement with those published by Nagaraj et al. [23] who evaluated the normal urinary proteome using LC–MS/MS techniques and found that intra- and inter-person variability contributed up to 45.5 and 47.1% of the total variations of proteome. The results of the analysis of serum MALDI-TOF/MS peptidome patterns presented here are timely data as no other study until now has examined serum peptidomic variations.

By logistic regression analyses, as expected, study results confirmed that the “bias” in naïve estimators of odds ratio was towards the null. Therefore, RCAL and SIMEX were applied. RCAL and SIMEX presented comparable odds ratio, with some expected discrepancies in the results. Considering the urinary peptidome, in which measurement error is more pronounced, RCAL tended to correct for a larger amount of bias effect with respect to SIMEX for MALDI-TOF/MS features; the same although less pronounced effect was found in sera peptidome. Our results are comparable to those reported by Beydoun and colleagues, who demonstrated that RCAL tends to correct for a larger amount of bias effect with respect to SIMEX [24].

When MALDI-TOF/MS urinary and serum peptidomic profiles were compared, we found that only 43 features were overlapping in the m/z 1000–4000, which is the wider range obtainable in the positive reflectron mode configuration. In that m/z range, peptides should be free to pass the glomerular filtration. These results may be explained in view of the fact that: (1) some small peptides may be reabsorbed by the kidneys; (2) peptides released in the urine may be diluted several times resulting in non-detectable concentrations.

Several studies have examined the MALDI-TOF/MS based peptidomic/proteomic profiling of urine or serum for PCa biomarkers discovery. Flatley and colleagues, for example, who used MALDI-MS profiling to analyse post-DRE urine samples in a m/z ranging between 2000 and 12,000, found a peak at m/z 10,760, corresponding to β-microseminoprotein that was statistically lower in the urine from PCa patients. The same peak was not present in pre-DRE urine [22]. When Nakayama et al. [25] performed MALDI-TOF/MS profiling of post-DRE urine, they found a candidate m/z 2331 peptide fragment, corresponding to the C-terminal PSA fragment. M’Koma et al. [26], who used MALDI profiling of urine to detect pre-neoplastic and neoplastic prostate disease, found a series of peptides at m/z 1373.1, 1433.5, 2236.3 and 2484.6 that were able to distinguish between PCa and BPH patients. Fania and colleagues, instead, found different MALDI-TOF/MS features in serum associated with PCa in patients with low (≤ 4 ng/mL) or high PSA levels (> 4 ng/mL), a result that could improve the diagnostic accuracy of this tumour marker, in particular by overcoming the false negative rate of PSA [27]. When Karbassi et al. [28] evaluated the serum proteins of patients with BPH and PCa using MALDI-TOF/MS, they found that the serum levels of the peptides at m/z 1216 and 1353 identified as fragments of ApoA-IV, were increased in the patients with BPH, while the kirinogen-1 peptide at m/z 1031 was decreased. Overall, these results uncovered high heterogeneity, even when evaluating the same matrix (urine or serum) in the same population of subjects. This effect might be explained by PCa heterogeneity, caused by the dynamics of a tumour cell population, characterized by a continuous accumulation of new, different mutations [29]. The results of our MALDI-TOF/MS-based peptidomic analysis of urine and serum appear to be consistent with the PCa heterogeneity hypothesis; the overall low diagnostic sensitivity of MALDI-TOF/MS-based peptidomics should not be attributed to low instrumental reproducibility as several strategies for reducing analytical variability and measurement errors have been carefully evaluated and successfully applied. To the best of our knowledge, the current one is first attempt to examine a combination of urinary and serum peptidome profiling using MALDI-TOF/MS analysis. Our results have confirmed that MALDI-TOF/MS serum peptidome is more sensitive than urinary peptidome in discriminating PCa in patients presenting LUTs. In particular, the serum feature at m/z 1832 was able to distinguish between BPH and PCa. The observation that urinary is less efficient than serum peptidome in identifying PCa deserves some comments. In fact, one might expect that the urine sample represents a more enriched and reliable source of biomarkers than serum in case of genitourinary track diseases. MALDI-TOF/MS results in the mass range evaluated in this study are most likely represented by peptides and they might be representative of protein cleavage caused by tumor-associated proteases. Even in the presence of equal amounts of tumor derived proteases released in sera and in urine, their activity might differ in these two biological matrices thus yielding to differences in peptide profiling, and this might be consequent to more constant pH and ions concentrations in serum than in urine. Accordingly, Caseiro et al. described in urine a higher number of proteases than in serum which contributed to biofluids’ proteome [30], and Kilkarni et al. [31] have shown that serum and urine harbor different physiological changes in response to radiation exposure when they evaluated the exosome proteome.

To characterize the significant serum features identified by using MALDI-TOF/MS instrument (Bruker Ultraflex II), a different MALDI-TOF/MS instrument (AB Sciex 4800 plus) was used, allowing the successful characterization of 12 features. Two of these features, at m/z 1739.9 and m/z 1896.0, matched with Complement C4-A. This finding is in line with previous data by Rosenzweig et al. [32] who reported that a truncated form of Complement C4-A was associated with PCa recurrence by examining serum proteome of 104 PCa patients.

## Conclusions

In conclusion, although the reproducibility of MALDI-TOF/MS has been largely criticized, we have demonstrated that measurement error and LOD issues can be handled by biostatistical approaches improving, at least in part, instrumental data reliability. We have also demonstrated that RCAL and SIMEX strategies, which have already being used in other fields of science, are applicable to peptidomic data to adjust the estimates obtained using statistical regression analysis. The results outlined here support the reliability of MALDI-TOF/MS profiling methods applied to clinical practice.

## Additional files

**Additional file 1: Materials and methods.** Urine and serum samples preparation before MALDI-TOF/MS analyses; within and between subject variability of serum MALDI-TOF/MS peptidomic features and variability of MALDI-TOF/MS serum peptidomic features; Within- and between-subjects variability of urinary MALDI-TOF/MS peptidomic analysis; Spectra processing; sLOD estimation of MALDI-TOF/MS peptidomic features; Simulation analyses to examine the reliability of ICC for datasets with measurement error and LOD issues; LOD adjustment, data normalization and log2 transformation of MALDI-TOF/MS features; RCAL and SIMEX for logistic regression analyses.

**Additional file 2: Intermediate data results.** Intermediate results generated step-by-step, following the manuscript details.

**Additional file 3: Table S1.** Monte Carlo simulation results. The ICC estimates were obtained by increasing the measurement error (σε) from 0.01 to 0.64 and considering three different limit of detection (LOD) conditions (12.5%, 25% and 50% of values set below LOD) using four different adjustment methods (Richardson and Ciampi’s method, Schisterman’s method, substitution of W < LOD by zeros and substitution of W < LOD by LOD/2). The mean ICCs and Monte Carlo standard errors are shown.

**Additional file 4: Figure S1.** The results of ICC estimation obtained by (a) varying the measurement error amount (x-axis of each graph); (b) by considering different strategies for handling limit of detection (LOD) issues; c) by considering three different LOD scenarios (12.5 %, 25% and 50% of values below LOD). The different strategies for handling LOD issues evaluated were: (1) sub W < LOD by E(W|W < LOD) = Richardson and Ciampi’s method; (2) sub W < LOD by E(W|W > LOD) = Schisterman’s method; (3) sub W < LOD by Zero and 4) sub W < LOD by LOD/2 (see Supplementary materials and methods for further details).

**Additional file 5: Results.** Monte Carlo simulations results confirmed that substituting the limit of detection (LOD) with LOD/2 does not affect the reliability of ICC estimation; The measurement error structure of peptidomi MALDI-TOF/MS-based analysis of the urinary and serum features

**Additional file 6: Figure S2.** Scatterplots of the within-subject replicates vs mean values and a QQ plot of the differences of between-subjects replicates of Urine.

**Additional file 7: Figure S3.** Scatterplots of the within-subject replicates vs mean values and a QQ plot of the differences of between-subjects replicates, Serum.

**Additional file 8: Table S2.** Free prostate specific antigen (fPSA), total PSA (tPSA), free to total PSA (f/tPSA) and prostate cancer antigen3 (PCA3) median and IQR values for the four classifications utilized in the PCa study.

**Additional file 9: Table S3.** A comparison of MALDI-TOF/MS serum and urinary features. Mean and standard error are reported in arbitrary units. Blank spaces are missing features.

**Additional file 10: Raw data 1.** Raw data of serum MALDI-TOF/MS features, patients’ diagnosis and age, Gleason grade, PSA and PCA3 values.

**Additional file 11: Raw data 2.** Raw data for estimating signal sLOD.

**Additional file 12: Raw data 3.** Data for estimating ICC and biological variation.

**Additional file 13: Table S4.** The significant MS-MS fragmentation patterns of the serum features analyzed using MALDI-TOF/MS set at CID conditions.

**Additional file 14: MS-Tag search results.** MS-MS spectra, peptide lists and MS-Tag search results (including all the configuration parameter) for the fragmentation patters of the 12 MALDI-TOF/MS serum features.

## References

[1] Gratzke C, Bachmann A, Descazeaud A, Drake MJ, Madersbacher S, Mamoulakis C (2015). EAU guidelines on the assessment of non-neurogenic male lower urinary tract symptoms including benign prostatic obstruction. Eur Urol.

[2] Martin SA, Haren MT, Marshall VR, Lange K, Wittert GA (2011). Members of the florey adelaide male ageing study. Prevalence and factors associated with uncomplicated storage and voiding lower urinary tract symptoms in community-dwelling Australian men. World J Urol.

[3] Zambon C-F, Basso D, Prayer-Galetti T, Navaglia F, Fasolo M, Fogar P (2006). Quantitative PSA mRNA determination in blood: a biochemical tool for scoring localized prostate cancer. Clin Biochem.

[4] Guess HA (2001). Benign prostatic hyperplasia and prostate cancer. Epidemiol Rev.

[5] Chevli KK, Duff M, Walter P, Yu C, Capuder B, Elshafei A (2014). Urinary PCA3 as a predictor of prostate cancer in a cohort of 3,073 men undergoing initial prostate biopsy. J Urol.

[6] Tanase CP, Codrici E, Popescu ID, Mihai S, Enciu AM, Necula LG (2017). Prostate cancer proteomics: current trends and future perspectives for biomarker discovery. Oncotarget.

[7] Ransohoff DF (2005). Bias as a threat to the validity of cancer molecular-marker research. Nat Rev Cancer.

[8] Banks RE, Clarke P, Selby PJ (2005). Influences of blood sample processing on low-molecular-weight proteome identified by surface-enhanced laser desorption/ionization mass spectrometry. Clin Chem.

[9] Fiedler GM, Baumann S, Leichtle A, Oltmann A, Kase J, Thiery J (2007). Standardized peptidome profiling of human urine by magnetic bead separation and matrix-assisted laser desorption/ionization time-of-flight mass spectrometry. Clin Chem.

[10] Calvano CD, Aresta A, Zambonin CG (2009). Optimization of analytical and pre-analytical conditions for MALDI-TOF-MS human urine protein profiles. J Pharm Biomed Anal.

[11] Markey MK (2011). Recent advances in computational analysis of mass spectrometry for proteomic profiling. J Mass Spectrom.

[12] Padoan A, Basso D, La Malfa M, Zambon C-F, Aiyetan P, Zhang H (2015). Reproducibility in urine peptidome profiling using maldi-tof. Proteomics.

[13] Carroll RJ (2006). Measurement error in nonlinear models—a model perspective.

[14] Carroll RJ (2005). Measurement error in epidemiologic studies. Encyclopedia of biostatistics.

[15] Richardson DB, Ciampi A (2003). Effects of exposure measurement error when an exposure variable is constrained by a lower limit. Am J Epidemiol.

[16] Schisterman EF, Vexler A, Whitcomb BW, Liu A (2006). The limitations due to exposure detection limits for regression models. Am J Epidemiol.

[17] Clinical Laboratory Standards Institute (CLSI) (2012). User verification of precision and estimation of bias.

[18] Padoan A, Seraglia R, Basso D, Fogar P, Sperti C, Moz S (2013). Usefulness of MALDI-TOF/MS identification of low-MW fragments in sera for the differential diagnosis of pancreatic cancer. Pancreas.

[19] Rosner B (2012). Fundamentals of biostatistics.

[20] Cook JR, Stefanski LA (1994). Simulation-extrapolation estimation in parametric measurement error models. J Am Stat Assoc.

[21] Capoluongo E, Zambon CF, Basso D, Boccia S, Rocchetti S, Leoncini E (2014). PCA3 score of 20 could improve prostate cancer detection: results obtained on 734 Italian individuals. Clin Chim Acta.

[22] Flatley B, Malone P, Cramer R (2014). MALDI mass spectrometry in prostate cancer biomarker discovery. Biochim Biophys Acta.

[23] Nagaraj N, Mann M (2011). Quantitative analysis of the intra- and inter-individual variability of the normal urinary proteome. J Proteome Res.

[24] Beydoun MA, Kaufman JS, Ibrahim J, Satia JA, Heiss G (2007). Measurement error adjustment in essential fatty acid intake from a food frequency questionnaire: alternative approaches and methods. BMC Med Res Methodol.

[25] Nakayama K, Inoue T, Sekiya S, Terada N, Miyazaki Y, Goto T (2014). The C-terminal fragment of prostate-specific antigen, a 2331 Da peptide, as a new urinary pathognomonic biomarker candidate for diagnosing prostate cancer. PLoS ONE.

[26] M’Koma AE, Blum DL, Norris JL, Koyama T, Billheimer D, Motley S (2007). Detection of pre-neoplastic and neoplastic prostate disease by MALDI profiling of urine. Biochem Biophys Res Commun.

[27] Fania C, Sogno I, Vasso M, Torretta E, Leone R, Bruno A (2015). A PSA-guided approach for a better diagnosis of prostatic adenocarcinoma based on MALDI profiling and peptide identification. Clin Chim Acta.

[28] Karbassi ID, Nyalwidhe JO, Wilkins CE, Cazares LH, Lance RS, Semmes OJ (2009). Proteomic expression profiling and identification of serum proteins using immobilized trypsin beads with MALDI-TOF/TOF. J Proteome Res.

[29] Shoag J, Barbieri CE (2016). Clinical variability and molecular heterogeneity in prostate cancer. Asian J Androl.

[30] Caseiro A, Ferreira R, Quintaneiro C, Pereira A, Marinheiro R, Vitorino R (2012). Protease profiling of different biofluids in type 1 diabetes mellitus. Clin Biochem.

[31] Kulkarni S, Koller A, Mani KM, Wen R, Alfieri A, Saha S (2016). Identifying urinary and serum exosome biomarkers for radiation exposure using a data dependent acquisition and SWATH-MS combined workflow. Int J Radiat Oncol Biol Phys.

[32] Rosenzweig CN, Zhang Z, Sun X, Sokoll LJ, Osborne K, Partin AW (2009). Predicting prostate cancer biochemical recurrence using a panel of serum proteomic biomarkers. J Urol.

