# Supplementary material for: MALDI-TOF peptidomic analysis of serum and post-prostatic massage urine specimens to identify prostate cancer biomarkers
Source: Clin Proteomics. 2018 Jul 25;15:23. doi: 10.1186/s12014-018-9199-8 (PMC6060548; doi:10.1186/s12014-018-9199-8)
Supplement: Supplementary file 1 — Additional file 1: Materials and methods. Urine and serum samples preparation before MALDI-TOF/MS analyses; within and between subject variability of serum MALDI-TOF/MS peptidomic features and variability of MALDI-TOF/MS serum peptidomic features; Within- and between-subjects variability of urinary MALDI-TOF/MS peptidomic analysis; Spectra processing; sLOD estimation of MALDI-TOF/MS peptidomic features; Simulation analyses to examine the reliability of ICC for datasets with measurement error and LOD issues; LOD adjustment, data normalization and log2 transformation of MALDI-TOF/MS features; RCAL and SIMEX for logistic regression analyses. [file 12014_2018_9199_MOESM1_ESM.doc]

**Supplementary Materials and Methods**

**Within- and between-subject variability of serum MALDI-TOF/MS-based peptidome**

After the sLOD thresholds were calculated as described below, two normalization approaches were used: median normalization, and sLOD adjustment followed by median normalization and by log_2_ transformation (sLMNLT features). Intraclass correlation coefficient (ICC), within- and between-subject variations were estimated by ANOVA.

**Within- and between-subject variability of urinary MALDI-TOF/MS-based peptidome**

After the sLOD thresholds were calculated as described below, four normalization approaches were used: a) median normalization, b) creatinine normalization (obtained by dividing samples MALDI-TOF/MS features’ signals with the corresponding creatinine level), c) sLOD adjustment followed by median normalization and by log_2_ transformation (sLMNLT features) and d) sLOD adjustment followed by creatinine normalization and by log_2_ transformation. Intraclass correlation coefficient (ICC), within- and between-subject variations were estimated by ANOVA.

**Spectra processing**

After the spectra in the MALDI-TOF/MS analysis were collected, peptidomic peak detection was first carried out using the version 3.3 Flex Analysis, (Bruker Daltonics, Bremen, Germany), using the SNAP algorithm and a S/N set to 2, after baseline subtraction and smoothing. Thereafter, peak alignment for all the spectra was performed using the 2009a MatLab version (The MathWorks, Inc., Natick, MA, USA) and a list of the m/z aligned peaks was retrieved. The spectra were then exported as raw data that were loaded into memory using a Matlab in-house routine; the baseline was subtracted and smoothed for high frequency using the Savitzky and Golay filter. Finally, the peak intensities were retrieved for each spectrum by evaluating the local maximum at the m/z specified position from the m/z peaks-list, in a sliding window of ±0.4 Da.

**The sLOD estimation of MALDI-TOF/MS peptidomic features**

The sLODs of the features of MALDI-TOF/MS peptidomic analysis of serum samples were determined as previously described elsewhere by us with regard to urinary sLOD estimation [12]. Briefly, three serum pools were independently prepared by ANC precipitation, diluted by ultrapure water up to 1/512 and subsequently analysed. The sLODs were calculated for both the urine and serum samples following commonly used methodology [12]. Polynomial regression from 2^nd^ to 5^th^ orders was used to calculate the best equation fitting data.

**Simulation analyses to examine the reliability of ICC for datasets with measurement error and LOD issues**

The classical additive error model states that X = W + U, where W is the surrogate measurement of X and U is the error associated with measurements with a mean of zero. However, as suggested by Carroll, a multiplicative error model is more appropriate for a log- normal distributed variable as the formula is W= X*U [14]. Therefore, the classical model holds after log-transformation. In RCAL, ICC are used to obtain the measurement error adjusted logistic regression coefficient β* by dividing the naïve β coefficient by ICC [14]. But ICC should be calculated for variables measured without errors which is not the case for MALDI-TOF/MS as the data is affected by both measurement error and LOD issues. By using the Monte Carlo simulation methods, we therefore tested a series of hypothetical conditions in which the measurement error variances σ_ε_ were increased from 0 to 0.64 (models with pure error) and a series of conditions in which the percentage of values below LOD varied between 12.5 % and 50% (model with a pure threshold). A series of conditions combining a mixture of models with pure measurement error and those with pure thresholds were generated. To perform these simulations, two variables, W_1_ and W_2_, were used and generated in accordance with Richardson and Ciampi’s article {Richardson:2003cu}. Starting from a “true” log normal distributed variable W, two variables, W_1_ and W_2_ were generated duplicating W, and both W_1_ and W_2_ were contaminated by a independently generated error component, namely ε_1_ and ε_2_ with lognormal (0, σ_ε_), in order to simulate two variables measured with error. The dataset where W_1_ and W_2_ are generated with σ_ε_ = 0 is considered the “full dataset”. To verify whether ICC is affected by measurement error and LOD issues, we derived Monte Carlo estimates of ICC (mean and SE), considering the following conditions:

1. substituting W < LOD by zeros
2. substituting W < LOD by LOD/2
3. substituting W < LOD by E(W|W < LOD){Richardson:2003cu}
4. substituting W < LOD by E(W|W > LOD){Schisterman:2006hv}

where E(W|W < LOD) and E(W|W > LOD) were the expected values of W when W is below or above the threshold level, respectively. Appropriate LOD thresholds were chosen to generate 12.5%, 25% and 50% of W values below the LOD threshold for the Monte Carlo simulations.

**LOD adjustment, data normalization and log_2_ transformation of MALDI-TOF/MS features**

Once the sLODs were estimated, the features from the lowest to the highest m/z for all the acquired MS spectra for the urine or serum spectra were sorted. Starting from the lowest m/z, each feature intensity was evaluated and the values below sLOD were substituted with the corresponding estimated feature’s sLOD divided by 2 (sLOD/2), calculated using the formula derived from the signal detection limit equations.

A dedicated R in-house function was developed to execute the sLOD adjustment.

**RCAL and SIMEX for logistic regression analyses**

Before applying the RCAL, the ICCs were estimated using the measurement error datasets of the features of MALDI-TOF/MS analysis. Each MALDI-TOF/MS feature signals obtained from the urine and serum samples were sLOD adjusted, median normalized, and log_2_ transformed. The R ("ICC" Package) was used to estimate the ICC and the corresponding within- and between-subjects variance. For each feature, the unbiased βˆ∗ coefficient estimation was obtained by dividing the βˆ naïve coefficients (obtained from the naïve logistic regression), with the corresponding ICC (βˆ∗ = βˆ/ICC). βˆ∗ confidence intervals were calculated as suggested by Rosner [19]. Cook and Stefanski proposed a simulation method (SIMEX) to estimate and reduce bias due to measurement error. SIMEX estimates are obtained extrapolating the relationship between the β coefficient and the magnitude of the measurement error in order to extrapolate back to the point when β is not affected by measurement error [20]. The measurement error variances used in the SIMEX models were calculated using the results from Nested ANOVA.
