# Supplementary material for: MALDI-TOF peptidomic analysis of serum and post-prostatic massage urine specimens to identify prostate cancer biomarkers
Source: Clin Proteomics. 2018 Jul 25;15:23. doi: 10.1186/s12014-018-9199-8 (PMC6060548; doi:10.1186/s12014-018-9199-8)
Supplement: Supplementary file 2 — Additional file 2: Intermediate data results. Intermediate results generated step-by-step, following the manuscript details. [file 12014_2018_9199_MOESM2_ESM.docx]

**Intermediate data Results**

1. **Estimating serum signal limit of detection (sLOD)**

*Data file used: PooledSerumsLOD.csv*

sLOD were calculated by the lowest values of MALDI-TOF/MS features. For each feature, the lowest features’ Abundances values were (output from R):

X_1020.52 X_1050.12 X_1060.07 X_1066.09 X_1072.11 X_1077.53 X_1206.57 X_1260.48 X_1263.59 X_1277.10 X_1350.62

13 23.876 29.078 20.569 21.262 36.508 24.343 24.778 9.997 10.527 35.357 9.951

14 11.084 41.485 21.768 47.016 33.434 15.467 17.231 16.667 11.460 20.039 11.141

15 8.716 8.676 10.230 16.334 5.826 12.930 7.507 4.890 8.498 9.023 4.748

X_1418.54 X_1465.65 X_1536.67 X_2858.49 X_3262.43

13 23.707 23.818 8.161 2.039 4.254

14 15.876 10.913 7.018 2.840 4.065

15 9.149 9.151 7.841 4.690 3.239

Estimating sLOD (by using the formula means+3sd), output from R

X_1020.52 X_1050.12 X_1060.07 X_1066.09 X_1072.11 X_1077.53 X_1206.57 X_1260.48 X_1263.59 X_1277.10 X_1350.62

19.512510 38.055588 18.276772 38.817421 37.973386 17.779083 21.240173 14.117642 7.352461 30.574654 9.406584

X_1418.54 X_1465.65 X_1536.67 X_2858.49 X_3262.43

19.050960 19.325607 4.721153 3.634310 2.736014

A robust 5^th^ order polynomial regression was fitted by the *rlm* command of the MASS library. The output from R for the fitted model was the following:

Call:

rlm(formula = LODs ~ poly(mz, 5, raw = TRUE))

Converged in 1 iterations

Coefficients:

(Intercept) poly(mz, 5, raw = TRUE)1 poly(mz, 5, raw = TRUE)2 poly(mz, 5, raw = TRUE)3

-6.713362e+02 2.268847e+00 -2.744564e-03 1.542773e-06

poly(mz, 5, raw = TRUE)4 poly(mz, 5, raw = TRUE)5

-4.071722e-10 4.069227e-14

Degrees of freedom: 16 total; 10 residual

Scale estimate: 12.4

1. **Reading the datafile MALDIPooledSera.csv, that contains the MALDI-TOF/MS features for PCa patients.**

*Data file used: Supplementary raw data.csv*

X_1011.04 X_1015.71 X_1020.54 X_1032.56 X_1035.61 X_1039.65 X_1055.50 X_1075.63 X_1091.61

1 253.341 61.509 182.330 117.049 40.009 18.892 93.294 278.613 63.613

2 217.200 126.052 44.697 135.787 25.993 56.358 245.348 849.048 30.636

3 24.883 639.055 50.007 70.326 24.046 204.014 192.722 65.216 59.530

4 37.587 143.970 194.560 76.550 39.631 40.491 107.341 1498.188 62.977

5 31.408 41.172 375.420 32.425 33.730 22.556 192.236 164.649 19.515

6 29.046 213.229 105.206 36.976 26.588 92.351 33.235 60.515 30.723

7 41.561 115.270 168.186 69.960 43.386 56.388 121.108 83.987 46.100

8 221.544 173.939 76.922 187.241 33.349 74.473 113.065 127.989 107.829

9 50.657 74.489 30.424 59.234 38.719 34.661 62.504 292.378 20.226

10 319.868 309.334 368.632 292.687 63.672 122.868 64.711 64.927 364.137

X_1098.60

1 526.792

2 162.168

3 40.424

4 52.816

5 1200.813

6 89.196

7 2014.956

8 605.567

9 5218.614

10 1631.372

1. **From *MALDIPooledSera* features values below sLOD were substituted by sLOD / 2.**

Features’ values below sLOD was substituted by sLOD/2. output from R, header of the first 10 features of the obtained datafile

X_1011.04 X_1015.71 X_1020.54 X_1032.56 X_1035.61 X_1039.65 X_1055.50 X_1075.63 X_1091.61 X_1098.60

1 253.34100 61.509 182.330 117.049 40.00900 13.56743 93.294 278.613 63.613 526.792

2 217.20000 126.052 44.697 135.787 13.66173 56.35800 245.348 849.048 30.636 162.168

3 14.41314 639.055 50.007 70.326 13.66173 204.01400 192.722 65.216 59.530 40.424

4 37.58700 143.970 194.560 76.550 39.63100 40.49100 107.341 1498.188 62.977 52.816

5 31.40800 41.172 375.420 32.425 33.73000 13.56743 192.236 164.649 19.515 1200.813

6 29.04600 213.229 105.206 36.976 13.66173 92.35100 33.235 60.515 30.723 89.196

7 41.56100 115.270 168.186 69.960 43.38600 56.38800 121.108 83.987 46.100 2014.956

8 221.54400 173.939 76.922 187.241 33.34900 74.47300 113.065 127.989 107.829 605.567

9 50.65700 74.489 30.424 59.234 38.71900 34.66100 62.504 292.378 20.226 5218.614

10 319.86800 309.334 368.632 292.687 63.67200 122.86800 64.711 64.927 364.137 1631.372

1. **Median normalization of MALDI-TOF/MS features**

sLOD Features values were normalized by median values and after log2 transformed, output from R, header of the first 10 features

X_1011.04 X_1015.71 X_1020.54 X_1032.56 X_1035.61 X_1039.65 X_1055.50 X_1075.63 X_1091.61 X_1098.60

1 2.4365310 0.3943198 1.96200235 1.3225630 -0.22615313 -1.7863304 0.99530660 2.57371297 0.4428439 3.4926838

2 2.6064432 1.8214381 0.32566903 1.9287645 -1.38436866 0.6601114 2.78224863 4.57326524 -0.2192810 2.1849083

3 -0.9880000 4.4824837 0.80674560 1.2986737 -1.06524415 2.8352118 2.75306490 1.18984150 1.0582324 0.4998276

4 -0.1528722 1.7845903 2.21903725 0.8732964 -0.07647660 -0.0455047 1.36102335 5.16396889 0.5917191 0.3378691

5 0.5614306 0.9519620 4.14073208 0.6074051 0.66433079 -0.6495542 3.17510511 2.95162032 -0.1251181 5.8181662

6 0.3814899 3.2574821 2.23829547 0.7297396 -0.70670930 2.0502780 0.57585374 1.44044318 0.4624695 2.0001294

7 0.4875389 1.9592537 2.50429425 1.2388388 0.54953812 0.9276967 2.03053080 1.50247457 0.6370753 6.0869131

8 2.1607528 1.8117410 0.63462768 1.9180559 -0.57112509 0.5879489 1.19031191 1.36917933 1.1219047 3.6114461

9 0.7775547 1.3338204 0.04200289 1.0032186 0.38983474 0.2301063 1.08074157 3.30655590 -0.5469959 7.4643159

10 2.3769195 2.3286082 2.58162411 2.2488015 0.04817379 0.9965520 0.07152568 0.07633325 2.5639241 4.7274567

1. **Recoding Diagnosis as binary (References = classes 0,1,3,4,6 while PCalesion = classes 2,8,5), output from R**

Diagnosibin

0 1

76 70

1. **Logistic regression using Diagnosibin as outcome by the glm(Diagnosibin~Features[,i], family = "binomial") command, output from R for the beta coefficients**

[1] -0.4057952375 0.2027988825 0.3183826987 -0.4142601749 -0.1426189036 0.1233217419 0.2041317108

[8] -0.0440884863 -0.0355233478 -0.1576488897 0.3676358147 0.0472739677 0.3848348038 0.1067695210

[15] -0.7937455059 0.3164569463 0.1361469635 0.3491564488 0.1145272573 0.0606640313 -0.9505501646

[22] 0.3389688227 -0.2675147081 -0.2697092587 0.1855215748 0.0167259017 0.4760011012 0.1907889493

[29] 0.0144230642 0.2572621062 0.1043074597 -0.5627283287 -0.7000361654 -0.1410494131 -0.3689605539

[36] -0.1441287408 0.1365059303 -0.0163300899 0.0676860848 0.0827851159 -0.6654464173 -0.3844399903

[43] 0.2973275956 -0.3587600706 -0.0877086683 0.5109671963 0.6939933941 0.2407274598 0.3188835160

[50] -0.0272126451 0.0096278186 0.3366768662 1.0279528374 0.0939363959 0.6168952577 -0.1983617054

[57] 0.2634737997 -0.0980259043 0.5972271300 0.0563966333 -0.1473815626 0.1071518443 -0.4107679333

[64] 0.5439884219 -0.3811237760 0.3522628781 -0.1726266618 0.0566965145 0.2195747857 0.3728014464

[71] -0.1746978815 0.0582790607 0.3001390554 0.2439292447 -0.1903216483 -0.6531583129 -0.1954160320

[78] 0.3396498529 0.2438874730 0.1806312339 0.4189049863 -0.0527216087 0.1279758607 0.2122101832

[85] 0.0555382421 0.2378632525 -0.0209483878 0.0873830099 0.0064401328 0.2365575814 -0.1580003572

[92] 0.2544835328 -0.4888588159 0.1529409552 -0.4101213545 0.2597780888 -0.0178580574 -0.2981934910

[99] -0.5031838382 -0.0180610045 -0.0006500767 0.0314304753 -0.1663788630 -0.6357351676 -1.2073068990

[106] -0.3616272188 -0.8233254611 -1.6551647439 -0.7182981459 -0.4951010036 -0.3720759995 -0.1884336660

[113] -0.1903828703 -0.0238396864 0.2150589344 -0.7934486919 0.2980889006 -0.9782396043 -0.0885410348

[120] -0.7253378473 -0.0310514080 -0.3242246023 0.1361006277 -0.0513253739 -1.0302692101 -0.9565428582

[127] -1.0145775303 -0.6402673125 -1.5290895130 -0.2644457696 0.0637274959 -0.1395489585 -0.0778261949

[134] -0.8167998698 -0.3534560729 0.3643787904 0.2487639045 0.2950692416 0.3664014484 0.3624023344

[141] 0.0558335356 -0.0230522846 -0.1381616665 0.1596442117 -0.0727886500 -0.0138515065 0.3206509174

[148] 0.3426710384 0.1569089512 0.1484948057 -0.0854728127 0.2137264286 0.0983646110 0.0054950665

[155] 0.1193617463 0.1438421337 -0.1033308256 0.0201223242 0.5224282774 -0.1182032454 0.1137075070

[162] 0.2628200108 0.1548126940 0.0937110831 0.2906658142 0.1299387090 0.2536095713 -0.0266534651

[169] 0.1743138942 0.0843733190 0.2976682198 0.2773604991 0.1842568411 0.0186322599 0.0300498090

[176] 0.2418202560 0.3144074588 0.1864698817 0.1124034853 0.3412771001 0.0405725407 0.2945273826

[183] 0.3681143676 0.3309835351 0.5236487919 0.5864586284

Output for the unadjusted p-value

[1] 4.288356e-02 1.623770e-01 4.721991e-03 1.498049e-01 5.034109e-01 4.351981e-01 1.685444e-01 6.804258e-01

[9] 8.224749e-01 5.671432e-02 6.042148e-02 7.176165e-01 7.613207e-02 6.478378e-01 5.391365e-03 8.274898e-02

[17] 6.285562e-01 1.691755e-01 1.628707e-01 3.028560e-01 9.963736e-04 4.274096e-02 2.492401e-01 3.559250e-01

[25] 6.301675e-02 8.962845e-01 8.095711e-02 1.122522e-01 9.394933e-01 1.472427e-01 7.600647e-01 1.179207e-01

[33] 2.933561e-02 6.271850e-01 2.668446e-02 5.621733e-01 5.781810e-01 8.844604e-01 7.156908e-01 1.662167e-01

[41] 4.660567e-04 6.023239e-02 3.283844e-01 5.429727e-02 6.216672e-01 6.504004e-02 2.634824e-03 1.341929e-01

[49] 1.514364e-03 9.069022e-01 9.726631e-01 1.579752e-02 3.673859e-03 9.602340e-02 7.595714e-03 1.284157e-01

[57] 6.080119e-02 6.884301e-01 1.975234e-02 8.748522e-01 6.110429e-01 5.588259e-01 2.653469e-03 8.094552e-02

[65] 3.212343e-02 2.666945e-02 3.626271e-02 6.322216e-01 3.122696e-01 2.649932e-02 4.145106e-02 6.972062e-01

[73] 3.233223e-01 2.432522e-01 4.160789e-01 8.150688e-04 7.841408e-03 3.850306e-02 9.371981e-02 2.879614e-02

[81] 7.918497e-02 7.242619e-01 5.175837e-01 2.635702e-01 7.528986e-01 1.677077e-01 8.861292e-01 4.450905e-01

[89] 9.087588e-01 6.736175e-02 4.260479e-01 3.449146e-01 2.106034e-03 2.134858e-01 1.685697e-01 1.224163e-03

[97] 8.796037e-01 2.429668e-01 1.082674e-02 8.716514e-01 9.903720e-01 7.462885e-01 4.138840e-01 1.842563e-02

[105] 2.996625e-04 1.590735e-02 2.582102e-04 1.382799e-05 1.100057e-03 5.776691e-03 9.795042e-04 3.537341e-02

[113] 4.066230e-01 6.585922e-01 1.160868e-01 2.938258e-03 7.256129e-04 3.759632e-03 5.435850e-01 2.686310e-03

[121] 8.620230e-01 6.834404e-04 1.214811e-01 8.024532e-01 1.624835e-03 1.501872e-03 1.554117e-03 6.467334e-04

[129] 1.316452e-04 1.600217e-03 5.256850e-01 1.221615e-02 6.081087e-01 4.773731e-03 1.346886e-01 1.573928e-01

[137] 8.547342e-02 1.303702e-02 1.214110e-01 1.482741e-02 6.597417e-01 9.093844e-01 5.715103e-01 5.075187e-01

[145] 6.703311e-01 8.861620e-01 1.911810e-01 2.552098e-02 4.128729e-01 4.502422e-01 6.882704e-01 2.921776e-01

[153] 6.876948e-01 9.516751e-01 2.266140e-01 4.791913e-01 6.912954e-01 9.020313e-01 1.786081e-02 4.281653e-01

[161] 3.534371e-01 1.334807e-01 1.055456e-01 5.025111e-01 1.248471e-01 3.079313e-01 1.734760e-02 8.704226e-01

[169] 3.016063e-01 4.834283e-01 4.593447e-02 7.185548e-02 2.019453e-01 9.115517e-01 8.628175e-01 4.955666e-02

[177] 7.263557e-02 8.376221e-02 5.709607e-01 1.021847e-01 8.750193e-01 1.908284e-02 1.045359e-01 7.332144e-03

[185] 4.116187e-03 7.316429e-03

Features with p-value < 0.05

[1] 1020.5 1192.4 1218.6 1367.8 1405.7 1418.6 1440.6 1460.7 1504.9 1591.1 1605.9 1719.0 1739.9 1818.9 1826.7 1832.1

[17] 1835.0 1838.9 1847.0 1886.0 1895.9 1902.9 1919.2 1934.1 1968.9 1977.1 1980.2 1984.5 1994.9 2006.3 2037.1 3156.6

[33] 3272.6 3681.0

Adjust p-value by the Benjaminis hockberg procedure (p.adjust function).

Overall results of the MALDI-TOF/MS serum features with BH p-value < 0.05:

| featID | m/z | beta | OR | BH adjusted P | naive p-value |
| --- | --- | --- | --- | --- | --- |
| 3 | 1020.5 | 0.3183827 | 1.374902334 | 0.004721991 | 0.031711214 |
| 15 | 1192.4 | -0.79374551 | 0.452148096 | 0.005391365 | 0.034579099 |
| 21 | 1218.6 | -0.95055016 | 0.386528311 | 0.000996374 | 0.016789963 |
| 41 | 1367.8 | -0.66544642 | 0.514043998 | 0.000466057 | 0.016789963 |
| 47 | 1405.7 | 0.69399339 | 2.001693143 | 0.002634824 | 0.022711531 |
| 49 | 1418.6 | 0.31888352 | 1.375591081 | 0.001514364 | 0.016789963 |
| 53 | 1440.6 | 1.02795284 | 2.795337463 | 0.003673859 | 0.027971666 |
| 55 | 1460.7 | 0.61689526 | 1.8531655 | 0.007595714 | 0.042812207 |
| 63 | 1504.9 | -0.41076793 | 0.663140807 | 0.002653469 | 0.022711531 |
| 76 | 1591.1 | -0.65315831 | 0.520399594 | 0.000815069 | 0.016789963 |
| 77 | 1605.9 | -0.19541603 | 0.822492404 | 0.007841408 | 0.042897113 |
| 93 | 1719 | -0.48885882 | 0.613325913 | 0.002106034 | 0.020616967 |
| 96 | 1739.9 | 0.25977809 | 1.296642315 | 0.001224163 | 0.016789963 |
| 105 | 1818.9 | -1.2073069 | 0.299001437 | 0.000299663 | 0.013934308 |
| 107 | 1826.7 | -0.82332546 | 0.438969449 | 0.00025821 | 0.013934308 |
| 108 | 1832.1 | -1.65516474 | 0.191060577 | 1.38E-05 | 0.002572007 |
| 109 | 1835 | -0.71829815 | 0.487581343 | 0.001100057 | 0.016789963 |
| 110 | 1838.9 | -0.495101 | 0.609509342 | 0.005776691 | 0.035815485 |
| 111 | 1847 | -0.372076 | 0.689301854 | 0.000979504 | 0.016789963 |
| 116 | 1886 | -0.79344869 | 0.45228232 | 0.002938258 | 0.023761568 |
| 117 | 1895.9 | 0.2980889 | 1.347281557 | 0.000725613 | 0.016789963 |
| 118 | 1902.9 | -0.9782396 | 0.375972377 | 0.003759632 | 0.027971666 |
| 120 | 1919.2 | -0.72533785 | 0.484160969 | 0.00268631 | 0.022711531 |
| 122 | 1934.1 | -0.3242246 | 0.723087817 | 0.00068344 | 0.016789963 |
| 125 | 1968.9 | -1.03026921 | 0.356910864 | 0.001624835 | 0.016789963 |
| 126 | 1977.1 | -0.95654286 | 0.384218892 | 0.001501872 | 0.016789963 |
| 127 | 1980.2 | -1.01457753 | 0.362555566 | 0.001554117 | 0.016789963 |
| 128 | 1984.5 | -0.64026731 | 0.527151491 | 0.000646733 | 0.016789963 |
| 129 | 1994.9 | -1.52908951 | 0.21673291 | 0.000131645 | 0.012243003 |
| 130 | 2006.3 | -0.26444577 | 0.767631277 | 0.001600217 | 0.016789963 |
| 134 | 2037.1 | -0.81679987 | 0.441843351 | 0.004773731 | 0.031711214 |
| 184 | 3156.6 | 0.33098354 | 1.392336867 | 0.007332144 | 0.042618086 |
| 185 | 3272.6 | 0.52364879 | 1.688176229 | 0.004116187 | 0.029446568 |
| 186 | 3681 | 0.58645863 | 1.797611121 | 0.007316429 | 0.042618086 |

1. **Reading the file for estimating the ICC and biological variation data (PBVEFLM.xls), output R of the header of the first 10 features**

X_1011_14 X_1020_54 X_1060_33 X_1066_27 X_1077_57 X_1206_62 X_1260_51 X_1263_64

1 27.606 205.334 30.594 45.468 102.416 590.182 750.244 59.086

2 33.754 127.694 11.678 16.142 56.278 277.546 345.884 29.124

3 16.244 146.268 8.762 19.940 57.158 410.350 165.934 37.098

4 21.874 119.492 71.894 281.246 36.582 201.588 143.026 21.052

5 11.076 75.922 12.926 11.790 33.688 181.660 36.268 17.820

6 14.506 138.630 15.022 15.618 52.292 397.658 88.206 26.484

7 19.020 310.942 13.248 11.866 70.720 428.972 121.746 16.628

8 10.960 284.102 19.414 10.104 63.168 579.806 79.718 28.082

9 16.372 197.972 11.832 14.372 43.112 793.152 190.260 28.380

10 17.754 247.022 17.494 13.472 40.044 936.932 108.462 39.130

X_1282_79 X_1350_68

1 75.866 147.860

2 43.206 87.512

3 22.608 148.332

4 17.118 43.284

5 12.376 51.862

6 10.604 106.024

7 11.694 69.612

8 13.696 87.616

9 28.456 102.288

10 13.094 91.008

1. **Results obtained after sLOD substitution to the PBVEFLM.xls datafile, output from R of the header of the first 10 features of the dataset**

X_1011_14 X_1020_54 X_1060_33 X_1066_27 X_1077_57 X_1206_62 X_1260_51 X_1263_64

1 14.41314 205.334 30.59400 45.46800 102.416 590.182 750.244 59.086000

2 33.75400 127.694 13.86341 13.76513 56.278 277.546 345.884 29.124000

3 14.41314 146.268 13.86341 13.76513 57.158 410.350 165.934 37.098000

4 14.41314 119.492 71.89400 281.24600 36.582 201.588 143.026 21.052000

5 14.41314 75.922 13.86341 13.76513 33.688 181.660 36.268 9.606462

6 14.41314 138.630 13.86341 13.76513 52.292 397.658 88.206 26.484000

7 14.41314 310.942 13.86341 13.76513 70.720 428.972 121.746 9.606462

8 14.41314 284.102 13.86341 13.76513 63.168 579.806 79.718 28.082000

9 14.41314 197.972 13.86341 13.76513 43.112 793.152 190.260 28.380000

10 14.41314 247.022 13.86341 13.76513 40.044 936.932 108.462 39.130000

X_1282_79 X_1350_68

1 75.866000 147.860

2 43.206000 87.512

3 22.608000 148.332

4 9.537306 43.284

5 9.537306 51.862

6 9.537306 106.024

7 9.537306 69.612

8 9.537306 87.616

9 28.456000 102.288

10 9.537306 91.008

1. **Results obtained for the PBVEFLM.xls datafile, after MALDI-TOF/MS features median normalization and log2 transformation, header of the first 10 features**

X_1011_14 X_1020_54 X_1060_33 X_1066_27 X_1077_57 X_1206_62 X_1260_51 X_1263_64

1 0.2617620 3.729142 0.5556282 0.8257601 1.860012 10.718499 13.625440 1.0730813

2 1.0009193 3.786555 0.4110967 0.4081821 1.668831 8.230169 10.256620 0.8636242

3 0.4967650 5.041290 0.4778180 0.4744305 1.970014 14.143172 5.719101 1.2786241

4 0.7532737 6.245009 3.7573952 14.6987561 1.911885 10.535591 7.474966 1.1002404

5 1.0194410 5.369962 0.9805590 0.9736071 2.382752 12.848809 2.565235 0.6794649

6 0.6977701 6.711367 0.6711567 0.6663984 2.531565 19.251452 4.270236 1.2821456

7 0.8884621 19.167248 0.8545757 0.8485170 4.359359 26.442914 7.504730 0.5921665

8 0.5072370 9.998311 0.4878907 0.4844317 2.223051 20.404927 2.805490 0.9882808

9 0.5933530 8.150014 0.5707222 0.5666759 1.774814 32.652093 7.832531 1.1683340

10 0.6688986 11.464031 0.6433864 0.6388250 1.858400 43.482028 5.033607 1.8159821

X_1282_79 X_1350_68

1 1.3778286 2.685337

2 1.2812027 2.595024

3 0.7792100 5.112428

4 0.4984481 2.262151

5 0.6745735 3.668198

6 0.4617208 5.132843

7 0.5879035 4.291059

8 0.3356434 3.083442

9 1.1714627 4.210942

10 0.4426163 4.223585

1. **Evaluation of ICC on the features obtained after sLOD adjustment, median normalization and log2 trasformation of features of the PBVEFLM.xls datafile.**

R output for the ICC results:

[1] 0.6467184 0.6167416 0.1634100 0.0000000 0.2900981 0.7288607 0.4586241 0.6991649

[9] 0.4424331 0.7443007 0.5078589 0.5970261 0.8021317 0.6915648 0.5270973 0.7501927

[17] 0.5585268 0.7792556 0.7320010 0.6154797

R output for the within subject variation results:

[1] 0.50753701 16.74643159 0.28118623 5.18631945 0.75579484 166.82368571

[7] 9.14848223 0.27109827 0.08699847 5.42766877 0.77688465 0.06320096

[13] 337.53925863 0.06811512 0.42242386 0.12025615 160.14720888 418.77507382

[19] 0.51625124 8.40862169

R output for the between subject variation results:

[1] 0.92909904 26.94845591 0.05492371 -0.01572378 0.30885209 448.44556357

[7] 7.75009421 0.63005400 0.06903388 15.79909761 0.80169658 0.09363542

[13] 1368.33901966 0.15272585 0.47083365 0.36113947 202.60916048 1478.32891499

[19] 1.41006630 13.45919811

Summary of results:

Min. 1st Qu. Median Mean 3rd Qu. Max.

0.4956 0.6161 0.5676 0.7296 0.8021

Min. 1st Qu. Median Mean 3rd Qu. Max.

0.0632 0.2787 0.7663 56.6036 11.0480 418.7751

Min. 1st Qu. Median Mean 3rd Qu. Max.

-0.0157 0.2698 0.8654 178.3473 18.5864 1478.3289

1. **Using SIMEX to calculate the unbiased logistic regression results, by the following command simex(logitresults, SIMEXvariable = c("features"), measurement.error = sigmau, B = 100). The Benjamini-Hockberg procedures was then used to estimate the adjusted p-value. Results were reported below:**

| featuresname | SIMEX coeff | SIMEX OR | SIMEX.pvalue | Coeff lower CI | Coeff upper CI | OR lower CI | OR upper CI | SIMEX.BH.Adj.Pvalue |
| --- | --- | --- | --- | --- | --- | --- | --- | --- |
| X_1020.54 | 0.40507 | 1.499407 | 0.005064 | 0.126171 | 0.683969 | 1.134476 | 1.981727 | 0.026909 |
| X_1192.47 | -1.49766 | 0.223652 | 0.001425 | -2.40014 | -0.59519 | 0.090705 | 0.551461 | 0.01152 |
| X_1218.62 | -1.92009 | 0.146593 | 6.71E-05 | -2.83657 | -1.00362 | 0.058627 | 0.36655 | 0.002496 |
| X_1367.83 | -1.03618 | 0.354807 | 0.000356 | -1.59144 | -0.48092 | 0.203632 | 0.618214 | 0.005522 |
| X_1405.75 | 1.198909 | 3.316496 | 0.000765 | 0.515512 | 1.882306 | 1.674495 | 6.568635 | 0.007973 |
| X_1418.67 | 0.370973 | 1.449143 | 0.003781 | 0.123979 | 0.617967 | 1.131992 | 1.855152 | 0.02344 |
| X_1440.65 | 2.20604 | 9.079685 | 0.000251 | 1.054588 | 3.357491 | 2.870792 | 28.71705 | 0.004668 |
| X_1460.71 | 1.108578 | 3.030048 | 0.002339 | 0.407333 | 1.809824 | 1.502804 | 6.109373 | 0.01554 |
| X_1504.92 | -0.54714 | 0.578603 | 0.003472 | -0.90801 | -0.18627 | 0.403326 | 0.830052 | 0.02227 |
| X_1591.10 | -1.03409 | 0.355548 | 0.000543 | -1.60697 | -0.46121 | 0.200494 | 0.630517 | 0.007528 |
| X_1605.93 | -0.22379 | 0.799482 | 0.008411 | -0.38795 | -0.05963 | 0.678443 | 0.942115 | 0.039112 |
| X_1719.00 | -0.68087 | 0.506177 | 0.002301 | -1.11084 | -0.2509 | 0.329284 | 0.7781 | 0.01554 |
| X_1739.98 | 0.301786 | 1.352271 | 0.00093 | 0.126835 | 0.476736 | 1.135229 | 1.610809 | 0.009105 |
| X_1818.96 | -2.61591 | 0.073101 | 7.08E-06 | -3.71575 | -1.51607 | 0.024337 | 0.219573 | 0.000439 |
| X_1826.72 | -1.42695 | 0.24004 | 5.09E-05 | -2.09651 | -0.75739 | 0.122884 | 0.46889 | 0.002369 |
| X_1832.15 | -3.52942 | 0.029322 | 8.80E-08 | -4.75643 | -2.3024 | 0.008596 | 0.100018 | 1.64E-05 |
| X_1835.09 | -1.19492 | 0.302728 | 0.000641 | -1.86598 | -0.52386 | 0.154744 | 0.59223 | 0.007528 |
| X_1838.90 | -0.7634 | 0.46608 | 0.004204 | -1.27779 | -0.24901 | 0.278653 | 0.779574 | 0.025224 |
| X_1847.08 | -0.47118 | 0.624264 | 0.000608 | -0.73463 | -0.20773 | 0.479683 | 0.812423 | 0.007528 |
| X_1886.02 | -1.39644 | 0.247477 | 0.00129 | -2.23018 | -0.56269 | 0.107509 | 0.569672 | 0.010906 |
| X_1895.98 | 0.332514 | 1.39447 | 0.001286 | 0.134039 | 0.53099 | 1.143437 | 1.700615 | 0.010906 |
| X_1902.99 | -2.09704 | 0.122819 | 0.000201 | -3.17415 | -1.01993 | 0.04183 | 0.360619 | 0.004668 |
| X_1919.28 | -1.29501 | 0.273894 | 0.001172 | -2.06144 | -0.52859 | 0.12727 | 0.589438 | 0.010901 |
| X_1934.12 | -0.39655 | 0.672635 | 0.000648 | -0.61944 | -0.17367 | 0.538247 | 0.840577 | 0.007528 |
| X_1968.98 | -2.05182 | 0.1285 | 0.000189 | -3.1011 | -1.00255 | 0.045 | 0.366942 | 0.004668 |
| X_1977.14 | -1.84702 | 0.157706 | 0.000251 | -2.81114 | -0.88291 | 0.060136 | 0.413579 | 0.004668 |
| X_1980.24 | -2.103 | 0.12209 | 0.000109 | -3.13894 | -1.06706 | 0.043329 | 0.344018 | 0.003389 |
| X_1984.50 | -0.96732 | 0.380102 | 0.000772 | -1.51909 | -0.41554 | 0.21891 | 0.659985 | 0.007973 |
| X_1994.92 | -3.24067 | 0.039138 | 3.24E-06 | -4.5518 | -1.92955 | 0.010548 | 0.145214 | 0.000302 |
| X_2006.36 | -0.29676 | 0.743225 | 0.002174 | -0.48309 | -0.11043 | 0.616877 | 0.895453 | 0.01554 |
| X_2037.19 | -1.65259 | 0.191553 | 0.000355 | -2.53799 | -0.76719 | 0.079025 | 0.464317 | 0.005522 |
| X_3156.62 | 0.444375 | 1.559516 | 0.006403 | 0.12959 | 0.759161 | 1.138361 | 2.136482 | 0.032186 |
| X_3272.66 | 0.823091 | 2.277529 | 0.002082 | 0.308534 | 1.337648 | 1.361428 | 3.810071 | 0.015489 |
| X_3681.04 | 1.053414 | 2.867425 | 0.001579 | 0.41241 | 1.694418 | 1.510454 | 5.443479 | 0.012239 |

Please note that seed was not set before the analyses, thus results are not exactly the same for repeated SIMEX analyses.

1. **Using regression calibration (RCAL) to calculate the unbiased regression coefficient. Regression calibration was implemented as suggested by Rosner B et al. (Bernard Rosner. (2012). *Fundamentals of biostatistics*. BROOKS/COLE, ed 2010 ) in chapter 12, pag 586 at “The Intraclass correlation coefficient”. Benjamini hockberg procedure was used to adjust p-value for multiple testing. The obtained results are outlined below:**

| featname | betastar | OR | Betastar U95CI | Betastar L95CI | pvalue | BH ajusted pvalue |
| --- | --- | --- | --- | --- | --- | --- |
| X_1020.54 | 0.51676 | 1.67659 | 0.15816 | 0.87536 | 0.00474 | 0.03233 |
| X_1192.47 | -1.28832 | 0.27573 | -2.19770 | -0.37894 | 0.00549 | 0.03522 |
| X_1218.62 | -1.54282 | 0.21378 | -2.46354 | -0.62211 | 0.00102 | 0.01728 |
| X_1367.83 | -1.08008 | 0.33957 | -1.68557 | -0.47458 | 0.00047 | 0.01691 |
| X_1405.75 | 1.12641 | 3.08456 | 0.39127 | 1.86155 | 0.00267 | 0.02306 |
| X_1418.67 | 0.62789 | 1.87366 | 0.23969 | 1.01609 | 0.00152 | 0.01730 |
| X_1440.65 | 1.66845 | 5.30397 | 0.53900 | 2.79791 | 0.00379 | 0.02875 |
| X_1460.71 | 1.00127 | 2.72175 | 0.26509 | 1.73745 | 0.00768 | 0.04295 |
| X_1504.92 | -0.66671 | 0.51339 | -1.10175 | -0.23168 | 0.00267 | 0.02306 |
| X_1591.10 | -1.06013 | 0.34641 | -1.68145 | -0.43881 | 0.00082 | 0.01705 |
| X_1605.93 | -0.31718 | 0.72820 | -0.55102 | -0.08333 | 0.00785 | 0.04295 |
| X_1719.00 | -0.79346 | 0.45228 | -1.29957 | -0.28735 | 0.00212 | 0.02076 |
| X_1739.98 | 0.42164 | 1.52446 | 0.16599 | 0.67729 | 0.00123 | 0.01730 |
| X_1818.96 | -1.95956 | 0.14092 | -3.02495 | -0.89418 | 0.00031 | 0.01451 |
| X_1826.72 | -1.33633 | 0.26281 | -2.05409 | -0.61856 | 0.00026 | 0.01451 |
| X_1832.15 | -2.68647 | 0.06812 | -3.90250 | -1.47045 | 0.00001 | 0.00277 |
| X_1835.09 | -1.16586 | 0.31165 | -1.86692 | -0.46479 | 0.00112 | 0.01730 |
| X_1838.90 | -0.80359 | 0.44772 | -1.37470 | -0.23248 | 0.00582 | 0.03607 |
| X_1847.08 | -0.60391 | 0.54667 | -0.96312 | -0.24471 | 0.00098 | 0.01728 |
| X_1886.02 | -1.28783 | 0.27587 | -2.13813 | -0.43754 | 0.00299 | 0.02420 |
| X_1895.98 | 0.48382 | 1.62227 | 0.20318 | 0.76447 | 0.00073 | 0.01691 |
| X_1902.99 | -1.58777 | 0.20438 | -2.66496 | -0.51057 | 0.00386 | 0.02875 |
| X_1919.28 | -1.17729 | 0.30811 | -1.94724 | -0.40734 | 0.00273 | 0.02306 |
| X_1934.12 | -0.52624 | 0.59082 | -0.83003 | -0.22246 | 0.00069 | 0.01691 |
| X_1968.98 | -1.67221 | 0.18783 | -2.71516 | -0.62927 | 0.00167 | 0.01730 |
| X_1977.14 | -1.55255 | 0.21171 | -2.51347 | -0.59163 | 0.00154 | 0.01730 |
| X_1980.24 | -1.64675 | 0.19268 | -2.66949 | -0.62400 | 0.00160 | 0.01730 |
| X_1984.50 | -1.03921 | 0.35373 | -1.63686 | -0.44155 | 0.00065 | 0.01691 |
| X_1994.92 | -2.48184 | 0.08359 | -3.75948 | -1.20421 | 0.00014 | 0.01306 |
| X_2006.36 | -0.42922 | 0.65102 | -0.69584 | -0.16260 | 0.00160 | 0.01730 |
| X_2037.19 | -1.32574 | 0.26561 | -2.24857 | -0.40290 | 0.00487 | 0.03233 |
| X_3156.62 | 0.53721 | 1.71123 | 0.14437 | 0.93006 | 0.00736 | 0.04295 |
| X_3272.66 | 0.84993 | 2.33948 | 0.26880 | 1.43105 | 0.00415 | 0.02968 |
| X_3681.04 | 0.95187 | 2.59056 | 0.25539 | 1.64835 | 0.00739 | 0.04295 |
