# Supplementary material for: MALDI-TOF peptidomic analysis of serum and post-prostatic massage urine specimens to identify prostate cancer biomarkers
Source: Clin Proteomics. 2018 Jul 25;15:23. doi: 10.1186/s12014-018-9199-8 (PMC6060548; doi:10.1186/s12014-018-9199-8)
Supplement: Supplementary file 3 — Additional file 3: Table S1. Monte Carlo simulation results. The ICC estimates were obtained by increasing the measurement error (σε) from 0.01 to 0.64 and considering three different limit of detection (LOD) conditions (12.5%, 25% and 50% of values set below LOD) using four different adjustment methods (Richardson and Ciampi’s method, Schisterman’s method, substitution of W < LOD by zeros and substitution of W < LOD by LOD/2). The mean ICCs and Monte Carlo standard errors are shown. [file 12014_2018_9199_MOESM3_ESM.doc]

**Supplementary Table 1:** Monte Carlo simulation results. The ICC estimates were obtained by increasing the measurement error (σε) from 0.01 to 0.64 and considering three different limit of detection (LOD) conditions (12.5%, 25% and 50% of values set below LOD) using four different adjustment methods (Richardson and Ciampi’s method, Schisterman’s method, substitution of W < LOD by zeros and substitution of W < LOD by LOD/2). The mean ICCs and Monte Carlo standard errors are shown.

| Parameter | Full Dataset | Substitution of  W < LOD by  E(W|W < LOD)^1^ | Substitution of  W < LOD by  E(W|W > LOD)^2^ | Substitution of  W < LOD by  zeros | Substitution of  W < LOD by  LOD/2 |
| --- | --- | --- | --- | --- | --- |
| **12.5% of values are set below LOD** | | | | | |
| σε = 0.01 | 0.961±0.006 | 0.955±0.008 | 0.887±0.022 | 0.924±0.013 | 0.954±0.007 |
| σε = 0.04 | 0.797±0.027 | 0.788±0.029 | 0.657±0.042 | 0.720±0.038 | 0.778±0.032 |
| σε = 0.16 | 0.609±0.048 | 0.596±0.048 | 0.455±0.063 | 0.516±0.060 | 0.584±0.052 |
| σε = 0.36 | 0.407±0.062 | 0.398±0.065 | 0.280±0.073 | 0.327±0.070 | 0.383±0.064 |
| σε = 0.64 | 0.281±0.069 | 0.279±0.068 | 0.179±0.076 | 0.215±0.075 | 0.259±0.076 |
| **25 % of values are set below LOD** | | | | | |
| σε = 0.01 | 0.961±0.006 | 0.947±0.009 | 0.857±0.025 | 0.936±0.012 | 0.952±0.008 |
| σε = 0.04 | 0.798±0.027 | 0.788±0.029 | 0.657±0.042 | 0.720±0.038 | 0.778±0.032 |
| σε = 0.16 | 0.604±0.050 | 0.584±0.053 | 0.388±0.069 | 0.525±0.063 | 0.568±0.058 |
| σε = 0.36 | 0.407±0.062 | 0.391±0.066 | 0.221±0.081 | 0.330±0.076 | 0.365±0.071 |
| σε = 0.64 | 0.280±0.068 | 0.264±0.069 | 0.140±0.083 | 0.213±0.077 | 0.249±0.072 |
| **50 % of values are set below LOD** | | | | | |
| σε = 0.01 | 0.961±0.006 | 0.928±0.015 | 0.808±0.035 | 0.945±0.012 | 0.945±0.011 |
| σε = 0.04 | 0.798±0.027 | 0.735±0.042 | 0.501±0.079 | 0.748±0.043 | 0.747±0.045 |
| σε = 0.16 | 0.608±0.048 | 0.543±0.058 | 0.285±0.095 | 0.540±0.066 | 0.535±0.067 |
| σε = 0.36 | 0.409±0.063 | 0.351±0.071 | 0.137±0.100 | 0.346±0.079 | 0.332±0.081 |
| σε = 0.64 | 0.283±0.068 | 0.235±0.076 | 0.071±0.090 | 0.229±0.082 | 0.224±0.081 |

^1^: Richardson and Ciampi’s method [15].

^2^: Schisterman’s method [16].
