# Supplementary figures and images for: MALDI-TOF peptidomic analysis of serum and post-prostatic massage urine specimens to identify prostate cancer biomarkers
Source: Clin Proteomics. 2018 Jul 25;15:23. doi: 10.1186/s12014-018-9199-8 (PMC6060548; doi:10.1186/s12014-018-9199-8)

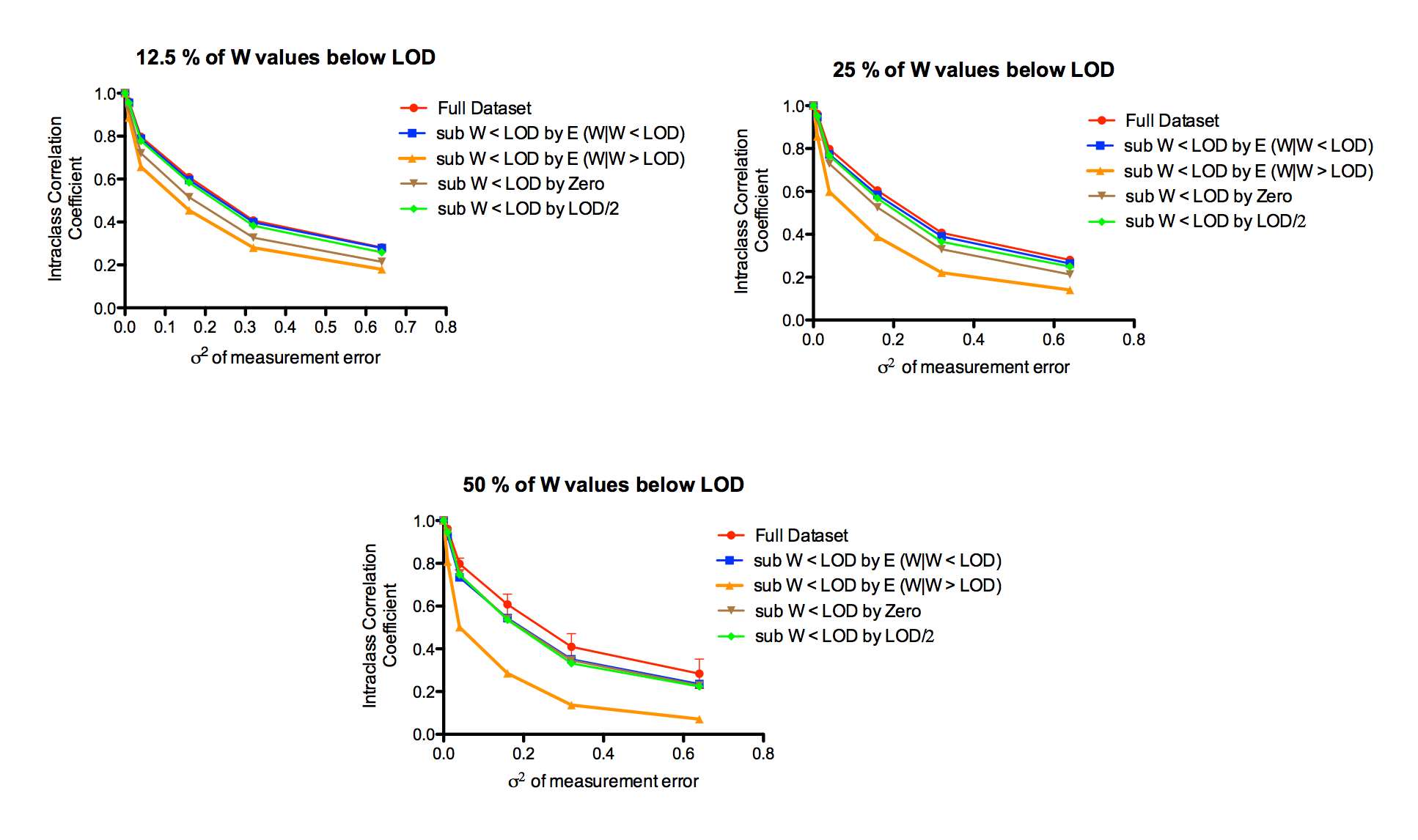

Supplement: Supplementary file 4 — Additional file 4: Figure S1. The results of ICC estimation obtained by (a) varying the measurement error amount (x-axis of each graph); (b) by considering different strategies for handling limit of detection (LOD) issues; c) by considering three different LOD scenarios (12.5 %, 25% and 50% of values below LOD). The different strategies for handling LOD issues evaluated were: (1) sub W < LOD by E(W|W < LOD) = Richardson and Ciampi’s method; (2) sub W < LOD by E(W|W > LOD) = Schisterman’s method; (3) sub W < LOD by Zero and 4) sub W < LOD by LOD/2 (see Supplementary materials and methods for further details). [file 12014_2018_9199_MOESM4_ESM.tif]

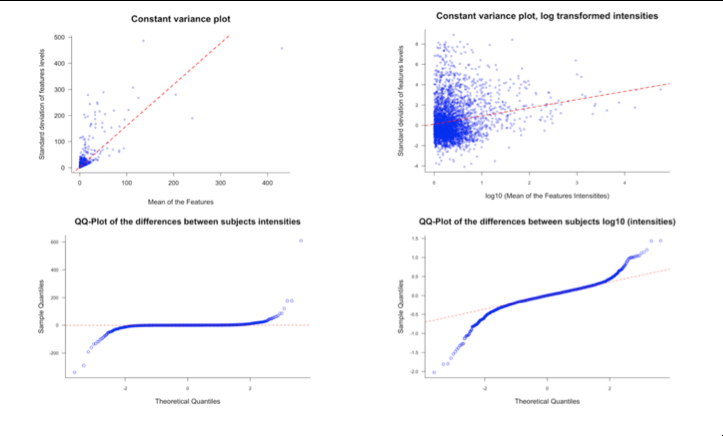

Supplement: Supplementary file 6 — Additional file 6: Figure S2. Scatterplots of the within-subject replicates vs mean values and a QQ plot of the differences of between-subjects replicates of Urine. [file 12014_2018_9199_MOESM6_ESM.tif]

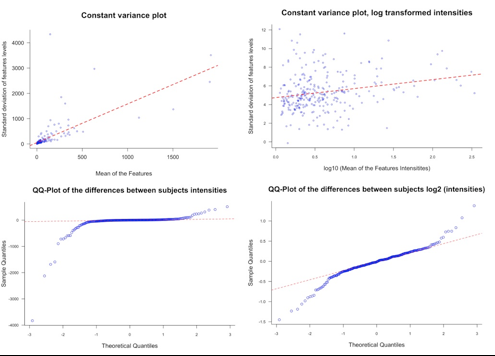

Supplement: Supplementary file 7 — Additional file 7: Figure S3. Scatterplots of the within-subject replicates vs mean values and a QQ plot of the differences of between-subjects replicates, Serum. [file 12014_2018_9199_MOESM7_ESM.tif]

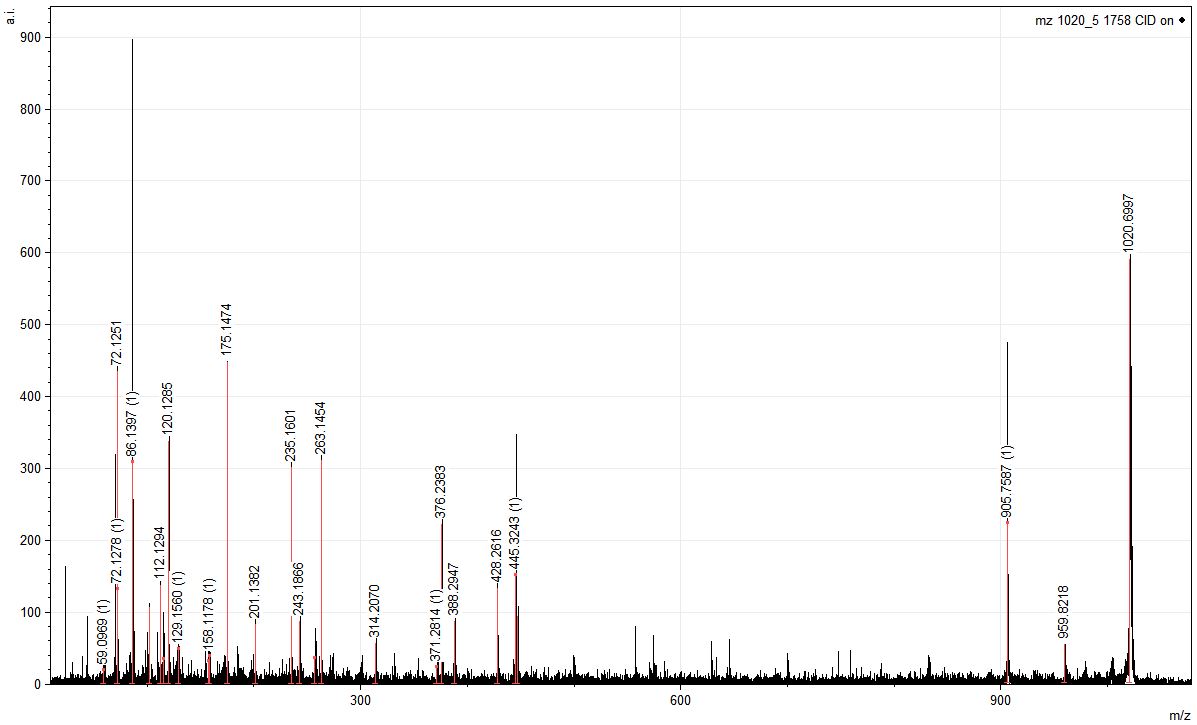

Supplement: Supplementary file 14 — Additional file 14: MS-Tag search results. MS-MS spectra, peptide lists and MS-Tag search results (including all the configuration parameter) for the fragmentation patters of the 12 MALDI-TOF/MS serum features. [file 12014_2018_9199_MOESM14_ESM.zip › New folder/1020_5 spectrum.JPG]

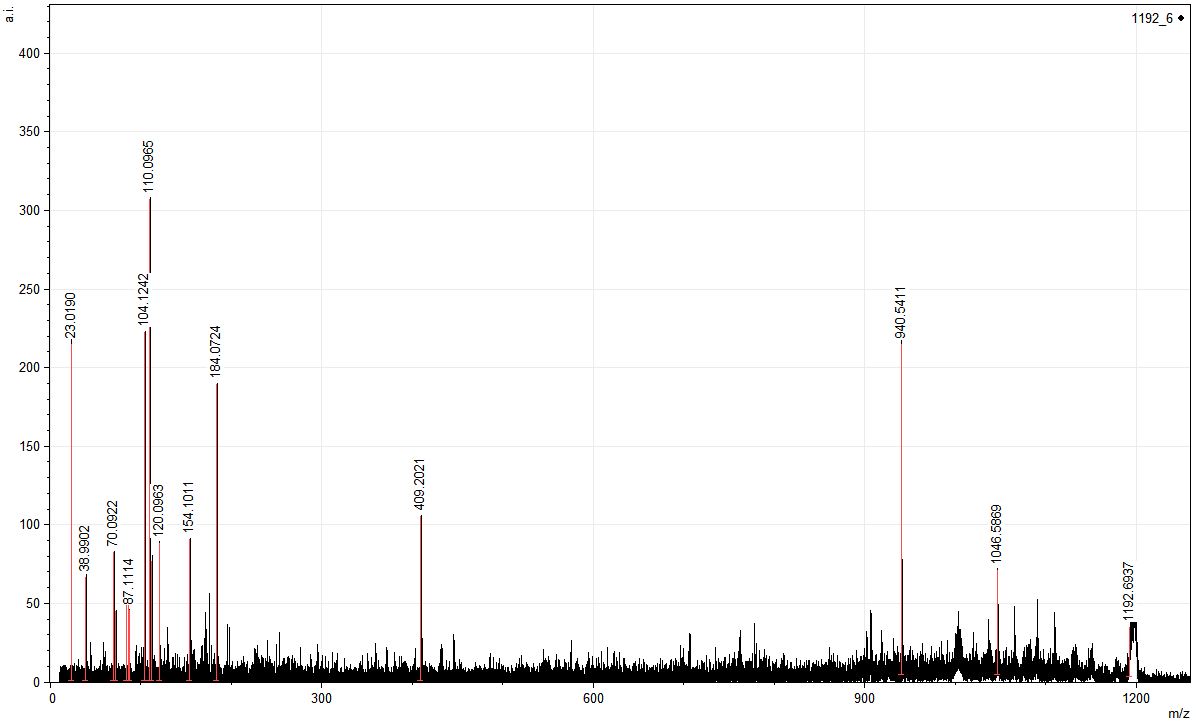

Supplement: Supplementary file 14 — Additional file 14: MS-Tag search results. MS-MS spectra, peptide lists and MS-Tag search results (including all the configuration parameter) for the fragmentation patters of the 12 MALDI-TOF/MS serum features. [file 12014_2018_9199_MOESM14_ESM.zip › New folder/1192_6 spectrum.JPG]

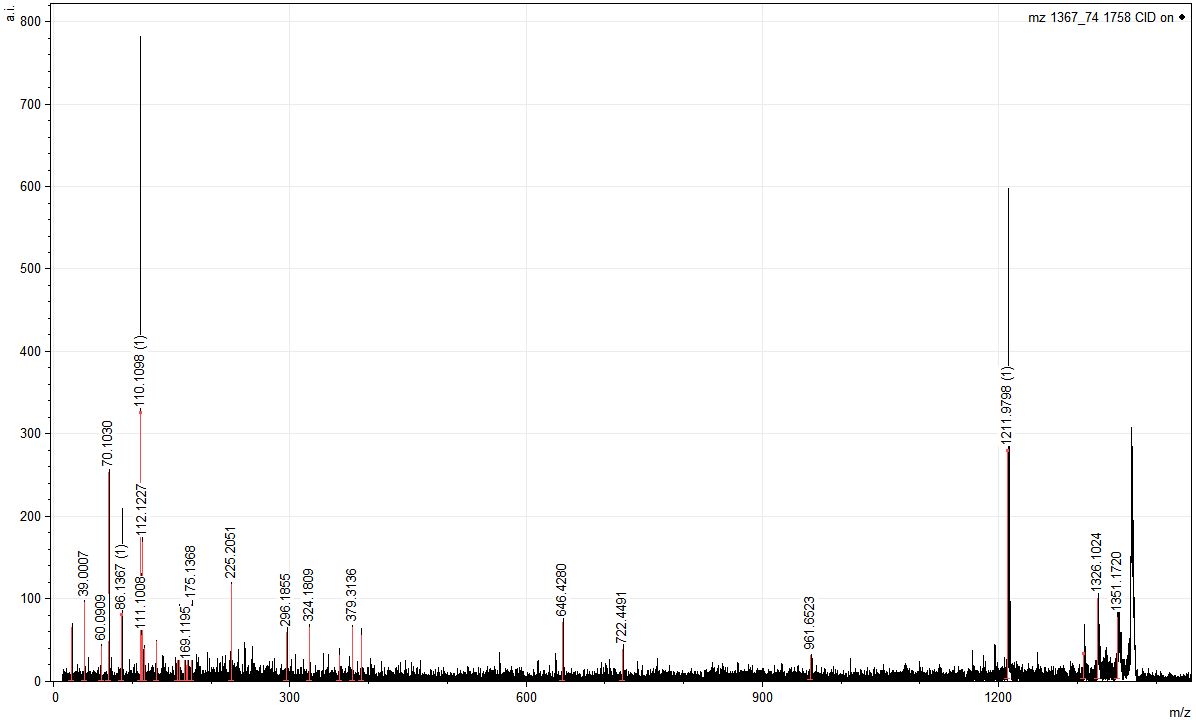

Supplement: Supplementary file 14 — Additional file 14: MS-Tag search results. MS-MS spectra, peptide lists and MS-Tag search results (including all the configuration parameter) for the fragmentation patters of the 12 MALDI-TOF/MS serum features. [file 12014_2018_9199_MOESM14_ESM.zip › New folder/1367_7 spectrum.JPG]

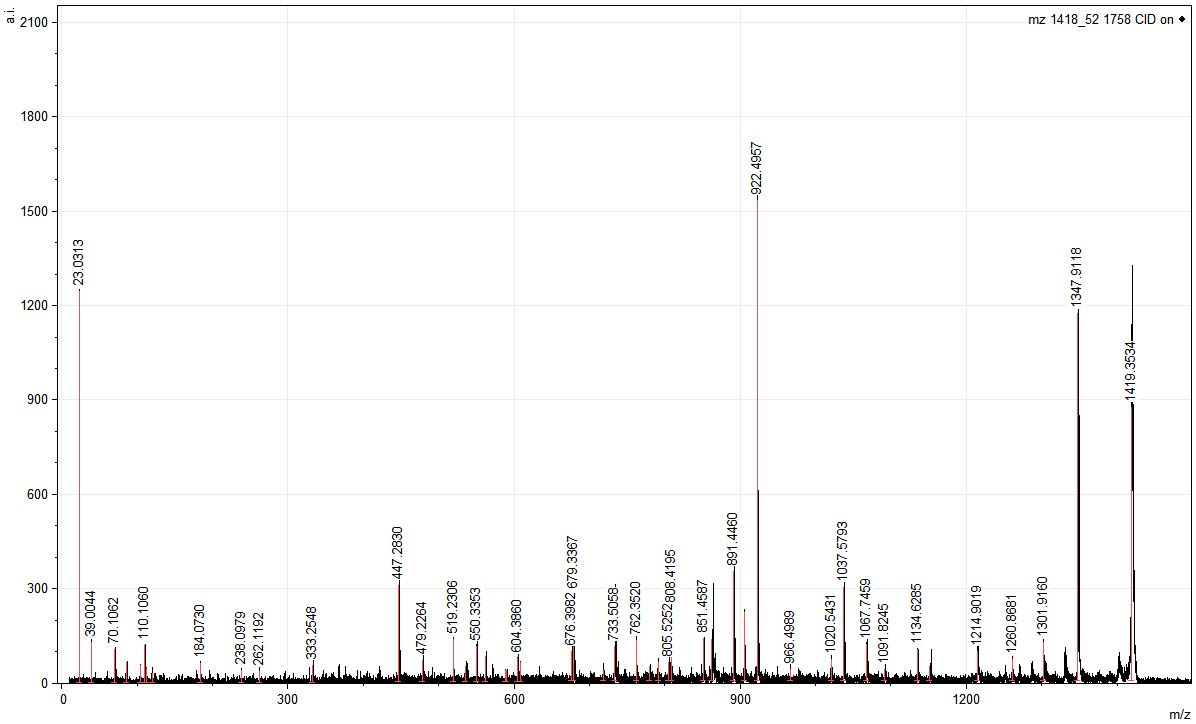

Supplement: Supplementary file 14 — Additional file 14: MS-Tag search results. MS-MS spectra, peptide lists and MS-Tag search results (including all the configuration parameter) for the fragmentation patters of the 12 MALDI-TOF/MS serum features. [file 12014_2018_9199_MOESM14_ESM.zip › New folder/1418_5 spectrum.JPG]

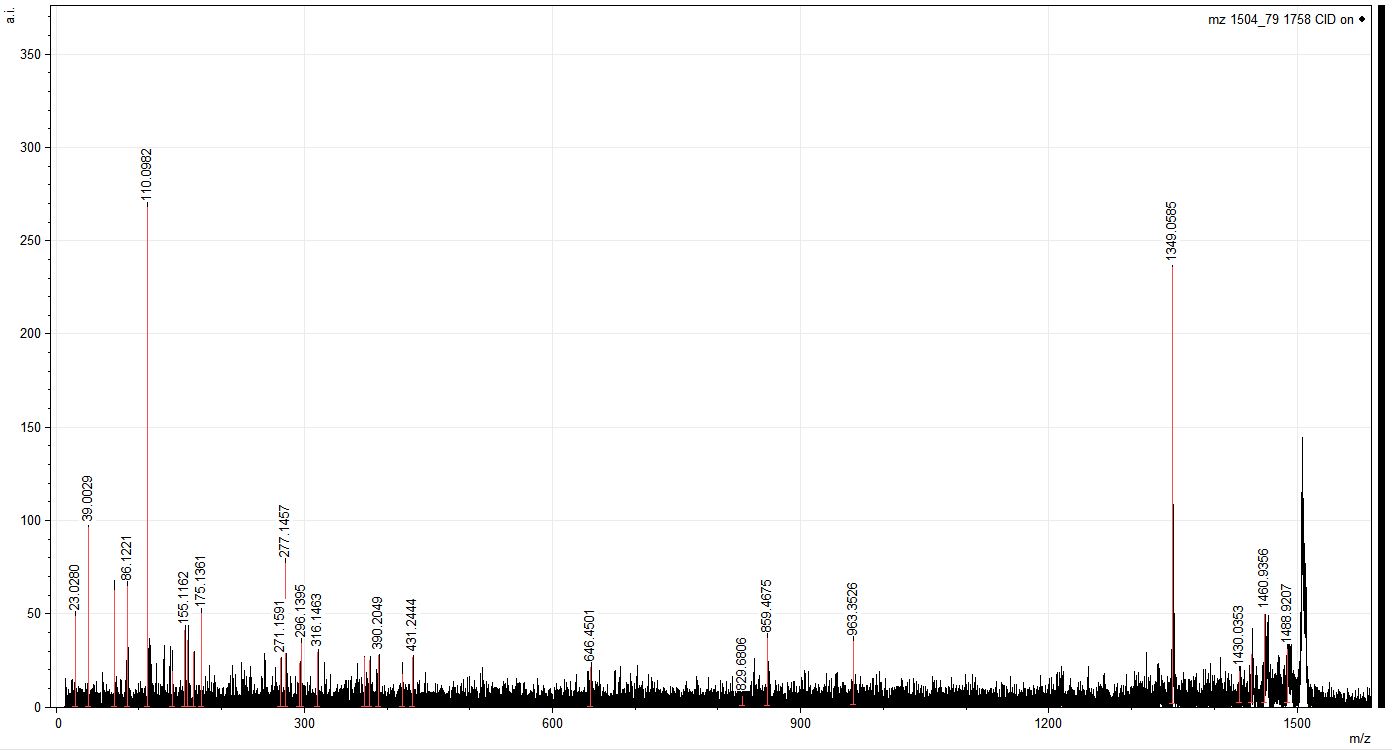

Supplement: Supplementary file 14 — Additional file 14: MS-Tag search results. MS-MS spectra, peptide lists and MS-Tag search results (including all the configuration parameter) for the fragmentation patters of the 12 MALDI-TOF/MS serum features. [file 12014_2018_9199_MOESM14_ESM.zip › New folder/1504_7 spectrum.JPG]

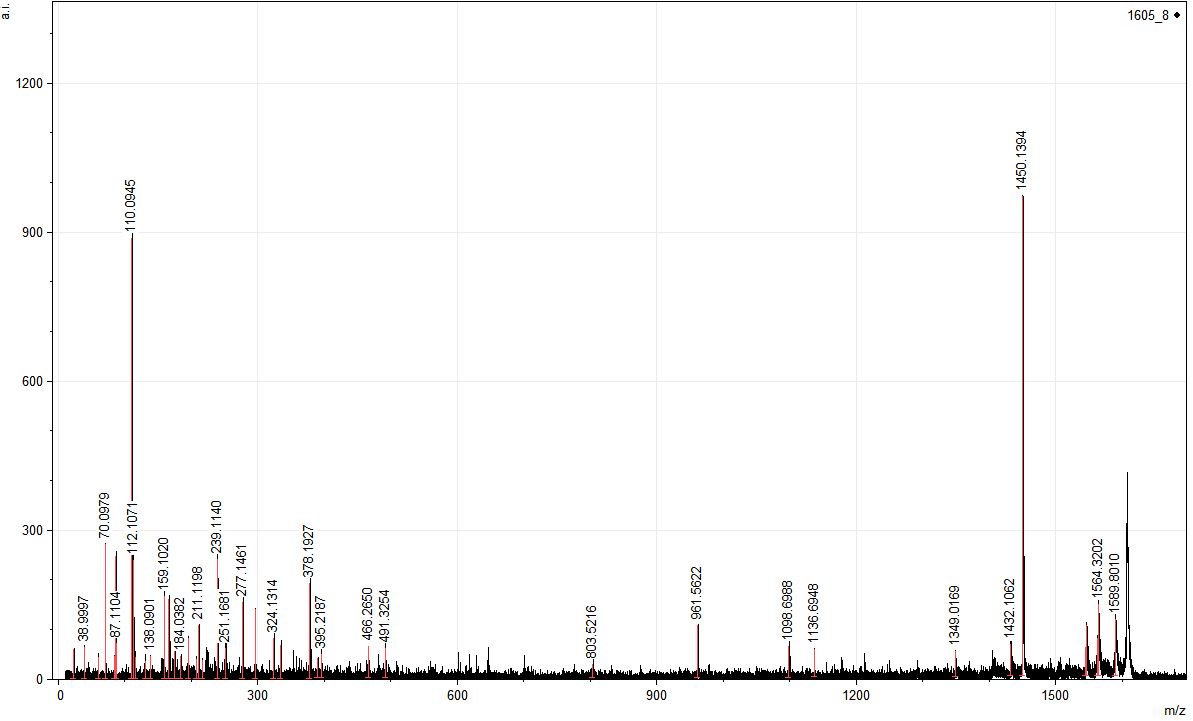

Supplement: Supplementary file 14 — Additional file 14: MS-Tag search results. MS-MS spectra, peptide lists and MS-Tag search results (including all the configuration parameter) for the fragmentation patters of the 12 MALDI-TOF/MS serum features. [file 12014_2018_9199_MOESM14_ESM.zip › New folder/1605_8 spectrum.JPG]

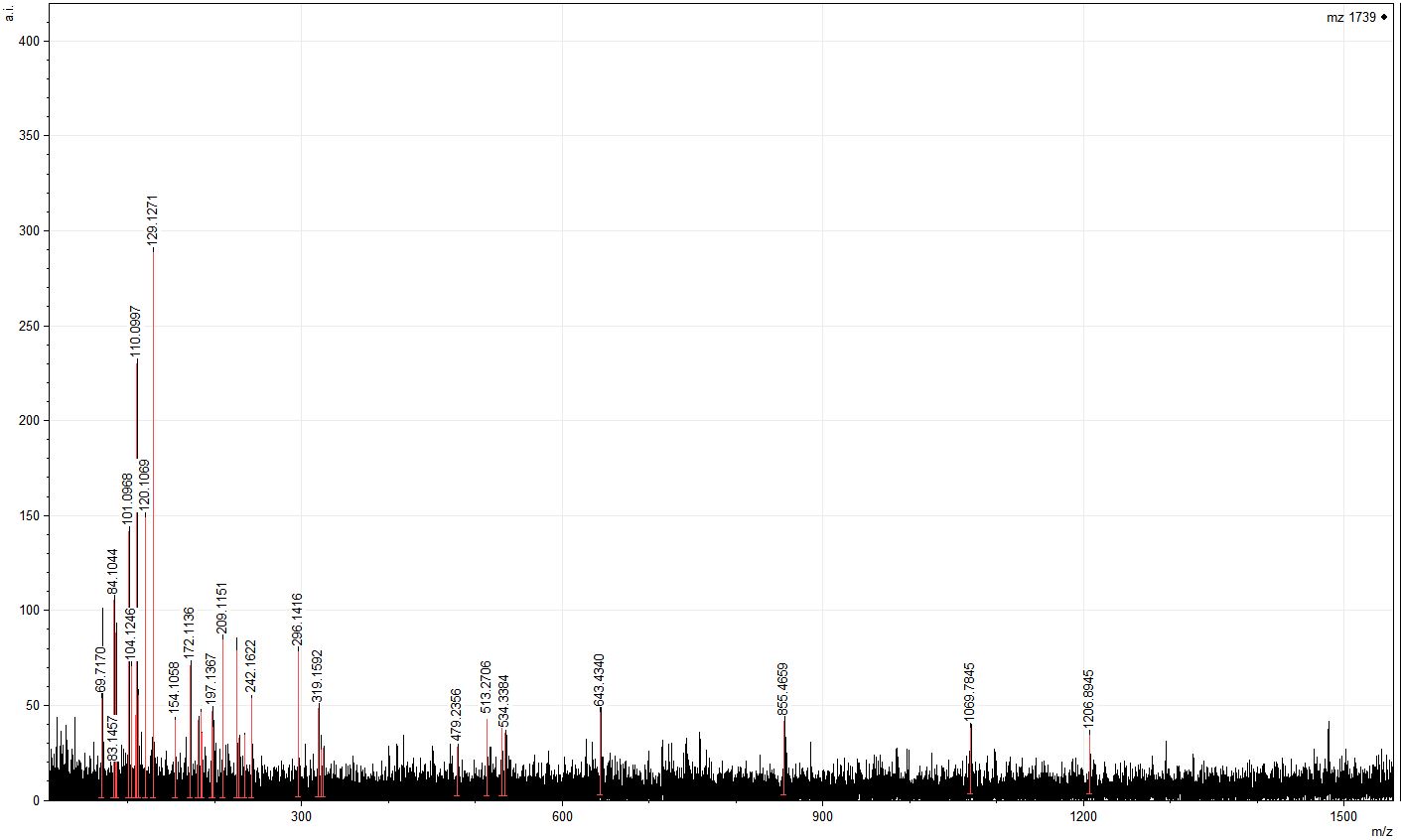

Supplement: Supplementary file 14 — Additional file 14: MS-Tag search results. MS-MS spectra, peptide lists and MS-Tag search results (including all the configuration parameter) for the fragmentation patters of the 12 MALDI-TOF/MS serum features. [file 12014_2018_9199_MOESM14_ESM.zip › New folder/1739_9 spectrum.JPG]

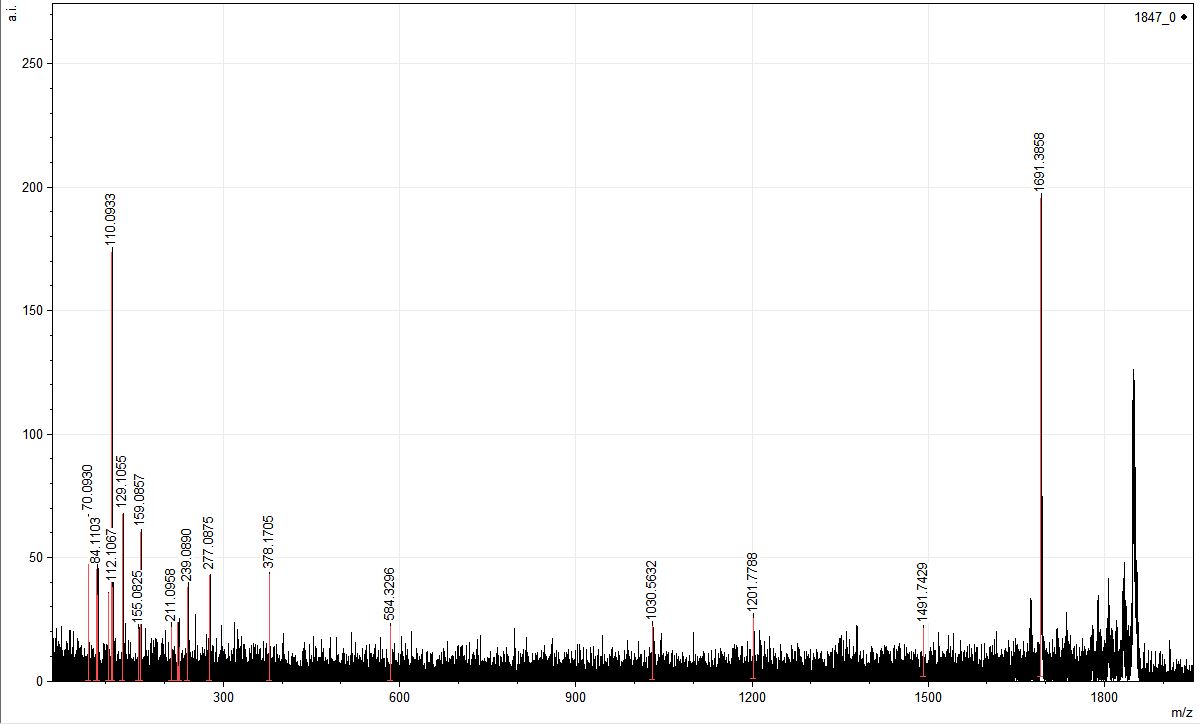

Supplement: Supplementary file 14 — Additional file 14: MS-Tag search results. MS-MS spectra, peptide lists and MS-Tag search results (including all the configuration parameter) for the fragmentation patters of the 12 MALDI-TOF/MS serum features. [file 12014_2018_9199_MOESM14_ESM.zip › New folder/1847_0 spectrum.JPG]

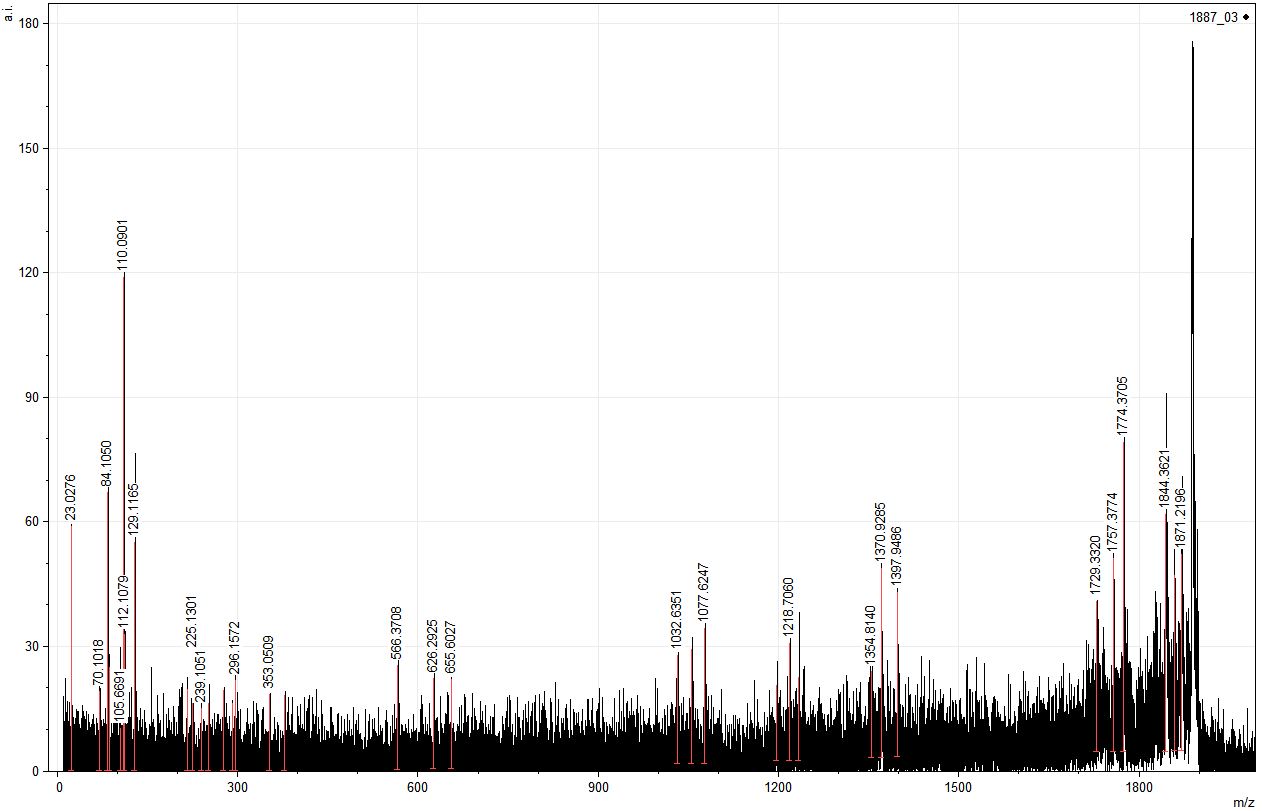

Supplement: Supplementary file 14 — Additional file 14: MS-Tag search results. MS-MS spectra, peptide lists and MS-Tag search results (including all the configuration parameter) for the fragmentation patters of the 12 MALDI-TOF/MS serum features. [file 12014_2018_9199_MOESM14_ESM.zip › New folder/1887_0 spectrum.JPG]

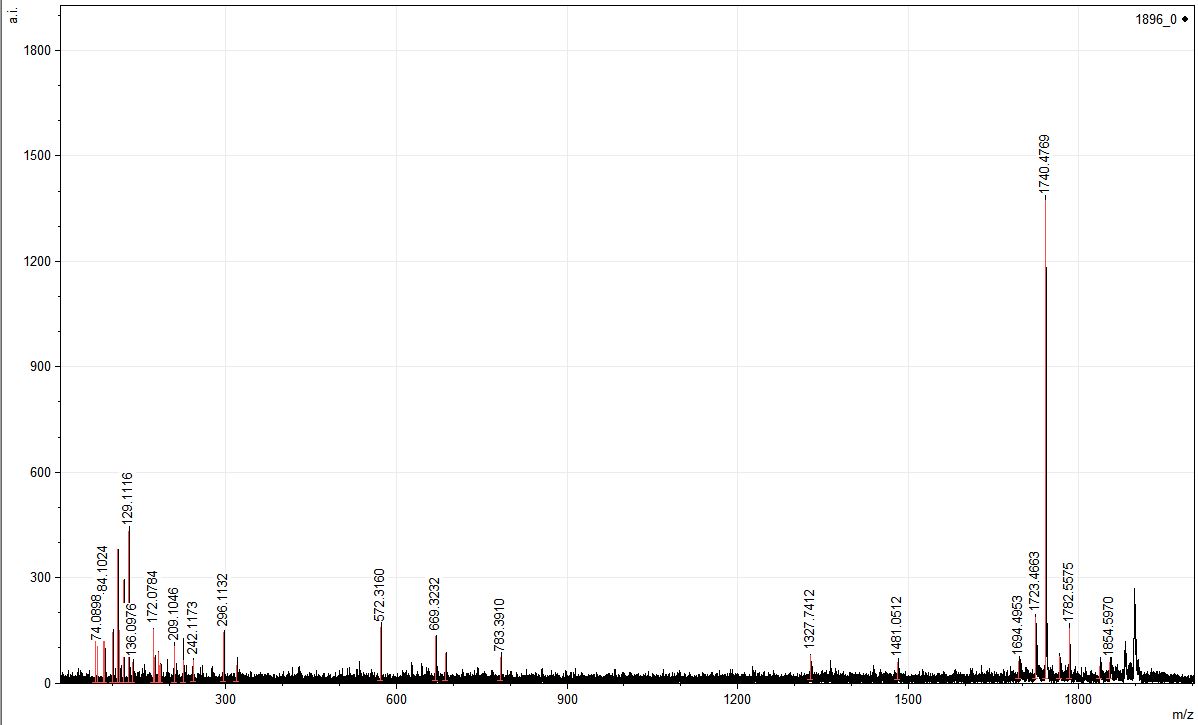

Supplement: Supplementary file 14 — Additional file 14: MS-Tag search results. MS-MS spectra, peptide lists and MS-Tag search results (including all the configuration parameter) for the fragmentation patters of the 12 MALDI-TOF/MS serum features. [file 12014_2018_9199_MOESM14_ESM.zip › New folder/1896_0 spectrum.JPG]

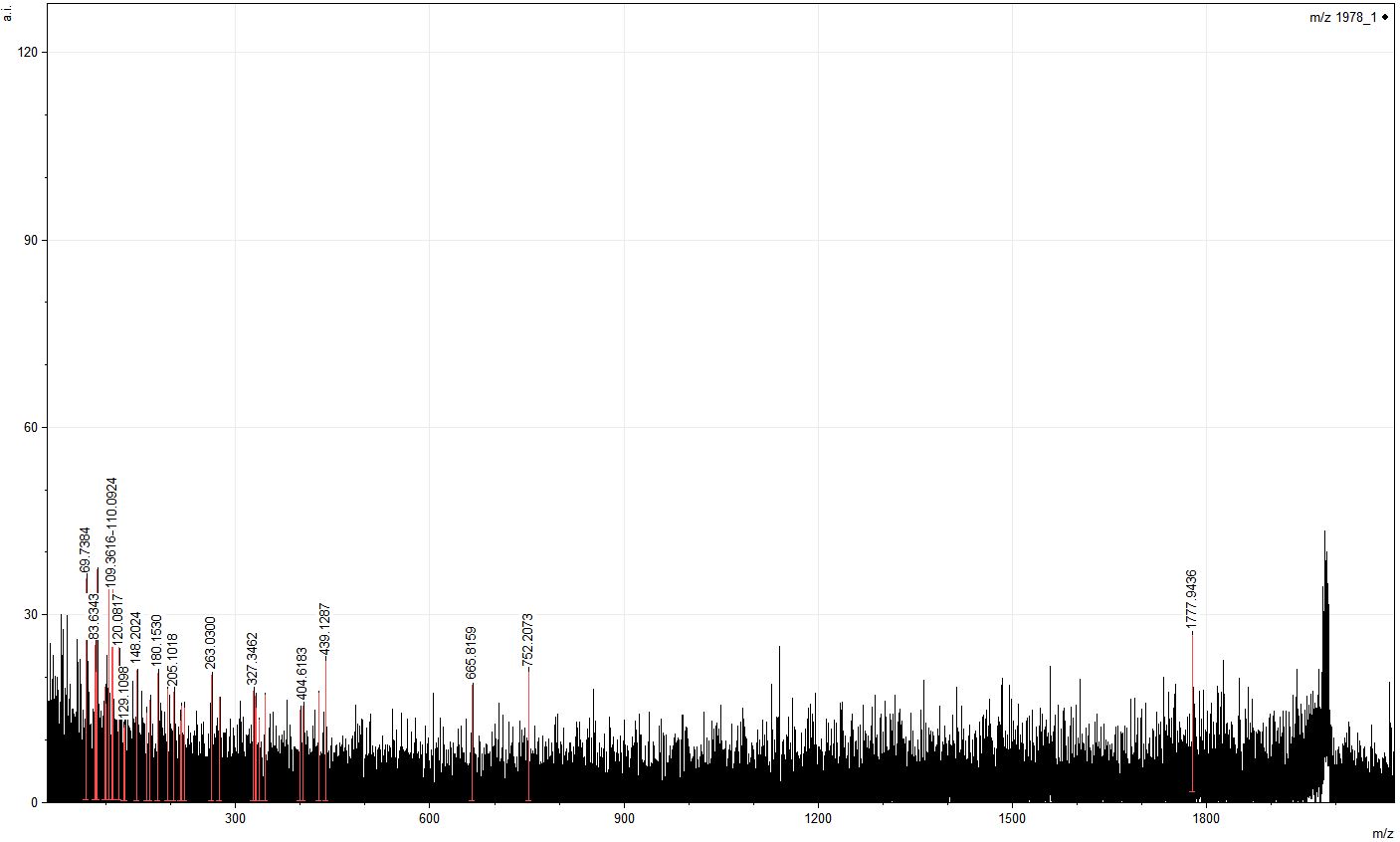

Supplement: Supplementary file 14 — Additional file 14: MS-Tag search results. MS-MS spectra, peptide lists and MS-Tag search results (including all the configuration parameter) for the fragmentation patters of the 12 MALDI-TOF/MS serum features. [file 12014_2018_9199_MOESM14_ESM.zip › New folder/1977_1 spectrum.JPG]

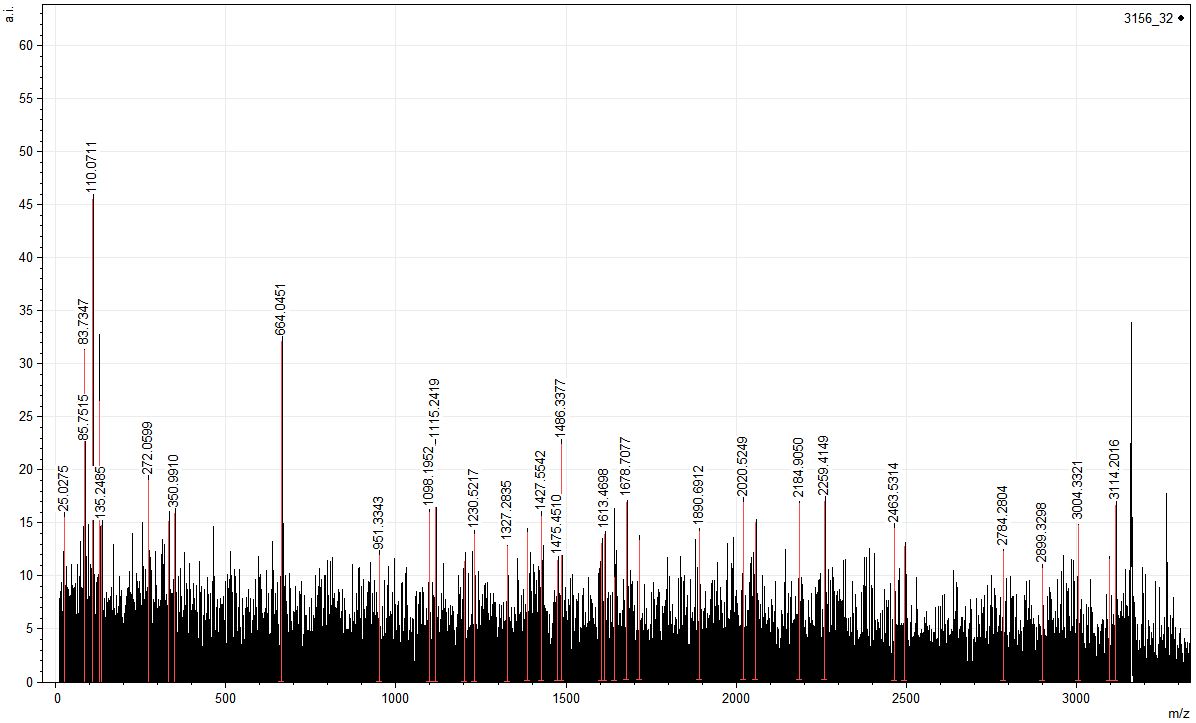

Supplement: Supplementary file 14 — Additional file 14: MS-Tag search results. MS-MS spectra, peptide lists and MS-Tag search results (including all the configuration parameter) for the fragmentation patters of the 12 MALDI-TOF/MS serum features. [file 12014_2018_9199_MOESM14_ESM.zip › New folder/3156_3 spectrum.JPG]

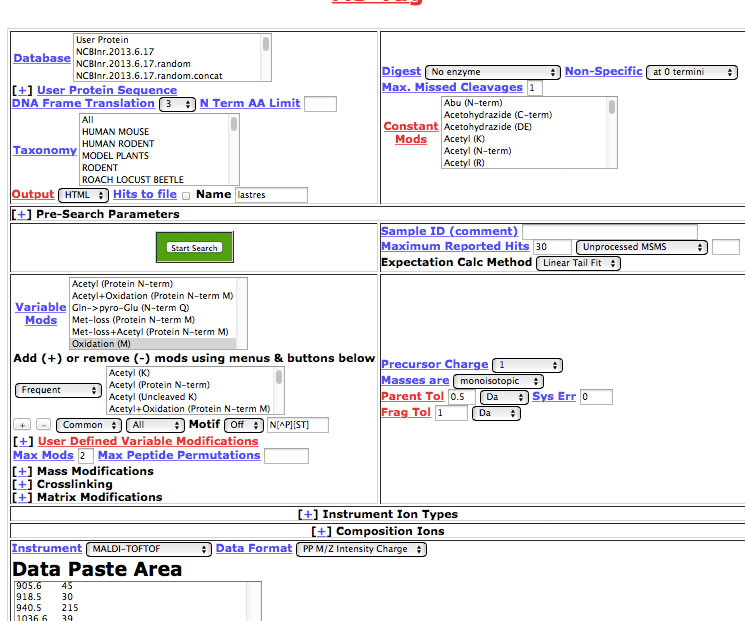

Supplement: Supplementary file 14 — Additional file 14: MS-Tag search results. MS-MS spectra, peptide lists and MS-Tag search results (including all the configuration parameter) for the fragmentation patters of the 12 MALDI-TOF/MS serum features. [file 12014_2018_9199_MOESM14_ESM.zip › New folder/MStag setting.jpeg]
