# Supplementary material for: MALDI-TOF peptidomic analysis of serum and post-prostatic massage urine specimens to identify prostate cancer biomarkers
Source: Clin Proteomics. 2018 Jul 25;15:23. doi: 10.1186/s12014-018-9199-8 (PMC6060548; doi:10.1186/s12014-018-9199-8)
Supplement: Supplementary file 5 — Additional file 5: Results. Monte Carlo simulations results confirmed that substituting the limit of detection (LOD) with LOD/2 does not affect the reliability of ICC estimation; The measurement error structure of peptidomi MALDI-TOF/MS-based analysis of the urinary and serum features [file 12014_2018_9199_MOESM5_ESM.doc]

**Supplementary Results**

**Monte Carlo simulations confirmed that substituting the limit of detection (LOD) with LOD/2 does not affect the reliability of ICC estimation**

According to the RCAL model, the intra-class correlation coefficient (ICC) is normally calculated (by ANOVA) to correctly estimate the error structure data error, both in internal or in external datasets [13]. Using a series of Monte Carlo simulation studies, we set out to verify if the ICC calculated using the ANOVA procedure was biased when applied to data affected by left censored issues. The mean values and standard deviation (sd) of all the sLMNLT MALDI-TOF/MS urinary features were 0.10 and 0.70, respectively. In order to perform simulations based on real conditions, a normal variable X (“true biomarker measurement”) was first generated with a mean of 0.1 and sd of 0.7; the two variables W1 and W2 (contaminated with random error) were then derived by X, as described in the Supplementary Material and Methods. Three independent sets of data with different percentages of left censored data (12.5%, 25% and 50%) were thus generated for testing different LOD conditions by choosing appropriate thresholds for W1 and W2. In Monte Carlo simulations, the measurement error variance (σ^2^ε) varied from 0.01 to 0.64, and the four different methods for handling left censoring were tested. All the conditions tested were compared to the “full dataset”, referring to a dataset without LOD issues. After N = 1000 iterations, the mean ICC and the corresponding standard error (SE) were calculated. The results are summarized in Supplementary Table 1 and in Supplementary Figure 1. The simulations showed that Richardson and Ciampi’s method [substitution of W < LOD by E(W|W < LOD)] outperformed the other methods, especially when the percentage of the left censored data was less than or equal to 25%. Shisterman’s method [Substitution of W < LOD by E(W|W > LOD)] and substituting the values below the LOD by zero had worse performances in all the conditions tested. Interestingly, the ICC results obtained for substituting the values below LOD by sLOD/2 were comparable to those obtained by the full dataset, indicating that ICC were not over- or under-estimating measurement error when LOD issues are correctly handled.

**The measurement error structure of peptidomic MALDI-TOF/MS-based analysis of the urinary and serum features**

As described by Carroll et al., measurement error structure should be evaluated for symmetry and constant variance; in particular, when W is measured in a replicated data set (replicated measurement of the same subjects), the sample standard deviation of the W-values should not be correlated with the individual mean. In cases of non-constant variance, it has been suggested that log transformation can be used [13]. With regard to the urinary and serum MALDI-TOF/MS peptidomic features used in the sets of data for the within-subject variability estimation, two replicates were used for each subject to calculate the median normalized standard deviation of the features’ signals and the corresponding average value. Supplementary Figure 2 (urine, upper left = before and upper right = after log_10_ transformation) and Supplementary Figure 3 (serum, upper left = before and upper right = after log_10_ transformation) show two scatterplots reporting features’ SD *vs* the mean before and after taking the log_10_ transformation. Supplementary Figure 2 (urine, lower left = before and lower right = after log_10_ transformation) and Supplementary Figure 3 (serum, lower left = before and lower right = after log_10_ transformation) show the QQ plot of the differences between subjects’ replicates, before and after log_10_ transformation of urinary and serum median normalized peptidome features’ signals. Urinary and serum results are similar. In particular, log transformations appeared to reduce the marked dependence of within-subject standard deviation *vs* mean, while the QQ plot indicated nearly normal distributed measurement error.

**Legend to the supplementary figures**

**Supplementary Figure 1:** The results of ICC estimation obtained by a) varying the measurement error amount (x-axis of each graph); b) by considering different strategies for handling limit of detection (LOD) issues; c) by considering three different LOD scenarios (12.5 %, 25% and 50% of values below LOD). The different strategies for handling LOD issues evaluated were: 1) sub W < LOD by E(W|W < LOD) = Richardson and Ciampi’s method; 2) sub W < LOD by E(W|W > LOD) = Schisterman’s method; 3) sub W < LOD by Zero and 4) sub W < LOD by LOD/2 (see Supplementary materials and methods for further details).

**Supplementary Figure 2:** Scatterplots of the within-subject replicates vs mean values and a QQ plot of the differences of between-subjects replicates, *Urine.*

**Supplementary Figure 3:** Scatterplots of the within-subject replicates vs mean values and a QQ plot of the differences of between-subjects replicates, *Serum.*
