# Supplementary material for: MALDI-TOF peptidomic analysis of serum and post-prostatic massage urine specimens to identify prostate cancer biomarkers
Source: Clin Proteomics. 2018 Jul 25;15:23. doi: 10.1186/s12014-018-9199-8 (PMC6060548; doi:10.1186/s12014-018-9199-8)
Supplement: Supplementary file 8 — Additional file 8: Table S2. Free prostate specific antigen (fPSA), total PSA (tPSA), free to total PSA (f/tPSA) and prostate cancer antigen3 (PCA3) median and IQR values for the four classifications utilized in the PCa study. [file 12014_2018_9199_MOESM8_ESM.doc]

**Supplementary Table 2**: Free prostate specific antigen (fPSA), total PSA (tPSA), free to total PSA (f/tPSA) and prostate cancer antigen3 (PCA3) median and IQR values for the four classifications utilized in the PCa study.

| **Group** | | **N (%)** | **fPSA**  (μg/L)  Median  (IQR)  (n=146) | **tPSA**  (μg/L)  Median  (IQR)  (n=146) | **f/tPSA**  (μg/L)  Median  (IQR)  (n=146) | **PCA3**  **score**  Median  (IQR)  (n=146) |
| --- | --- | --- | --- | --- | --- | --- |
| **A** | No Alterations | 57 (39.0%) | 0.74  (0.43-1.31) | 5.05  (3.61-7.6) | 15.70  (10.10-19.25) | 23.50  (13.00-46.50) |
| **B** | BPH + Inflammation | 19 (13.0%) | 0.51  (0.41-1.33) | 4.97  (3.11-9.3) | 14.90  (9.30-13.98) | 29.50  (19.50-48.75) |
| **C** | HGPIN+ASAP | 15 (10.3%) | 0.71  (0.36-1.08) | 6.34  (4.33-8.54) | 11.30  (8.65-15.05) | 20.00  (13.50-51.5) |
| **D** | PCa | 55 (37.7%) | 0.43  (0.27-0.83) | 6.54  (4.09-8.86) | 8.10  (5.35-10.95) | 50.00  (24.00-88.00) |
|  |  |  | p = n.s. | p = n.s. | p = 0.023 for A vs D, p= 0.028 B vs D and  p = 0.018 for C vs D | p = 0.023 for A vs D |

BPH=benign prostatic hyperplasia; Inflammation = chronic inflammation; ASAP = Atypical small acinar proliferation; HGPIN = high-grade prostatic intraepithelial neoplasia; PCa = prostatic neoplasia.

The two patients with atypical adenomatous hyperplasia (AAH) were left out from grouping, because this condition was initially assumed to be a precursor of adenocarcinoma, while actually it is consider a benign condition (Kowalewski A, Szylberg Ł, Skórczewska A, Marszałek A. Diagnostic Difficulties With Atrophy, Atypical Adenomatous Hyperplasia, and Atypical Small Acinar Proliferation: A Systematic Review of Current Literature. Clin Genitourin Cancer. 2016 Oct;14(5):361-365).
