# Supplementary material for: MALDI-TOF peptidomic analysis of serum and post-prostatic massage urine specimens to identify prostate cancer biomarkers
Source: Clin Proteomics. 2018 Jul 25;15:23. doi: 10.1186/s12014-018-9199-8 (PMC6060548; doi:10.1186/s12014-018-9199-8)
Supplement: Supplementary file 9 — Additional file 9: Table S3. A comparison of MALDI-TOF/MS serum and urinary features. Mean and standard error are reported in arbitrary units. Blank spaces are missing features. [file 12014_2018_9199_MOESM9_ESM.doc]

**Supplementary Table 3:** A comparison of MALDI-TOF/MS serum and urinary features. Mean and standard error are reported in arbitrary units. Blank spaces are missing features.

| **Serum MALDI-TOF/MS features, sLOD adjusted, Median normalized intensities** | | | **Urine MALDI-TOF/MS features, sLOD adjusted, Median normalized intensities** | | |
| --- | --- | --- | --- | --- | --- |
| **Serum feature's mz** | **Mean** | **standard error** | **Urine features’ mz** | **Mean** | **standard error** |
| 1011.0 | 40.88 | 0.33 |  |  |  |
| 1015.7 | 205.30 | 0.99 |  |  |  |
| 1020.5 | 247.93 | 3.08 | 1020.4 | 82.60 | 0.53 |
| 1032.5 | 67.30 | 0.38 |  |  |  |
| 1035.6 | 28.40 | 0.16 |  |  |  |
| 1039.6 | 75.03 | 0.30 |  |  |  |
| 1055.5 | 114.55 | 0.94 |  |  |  |
| 1075.6 | 343.74 | 4.27 | 1075.6 | 99.04 | 0.43 |
| 1091.6 | 60.14 | 0.43 |  |  |  |
| 1098.6 | 816.60 | 35.27 |  |  |  |
| 1122.6 | 27.38 | 0.26 | 1122.7 | 676.42 | 8.80 |
| 1136.5 | 106.26 | 1.07 |  |  |  |
| 1140.6 | 33.81 | 0.22 |  |  |  |
| 1171.5 | 27.18 | 0.15 | 1171.6 | 115.25 | 0.58 |
| 1192.4 | 47.82 | 0.74 | 1192.5 | 206.42 | 2.01 |
| 1196.8 | 48.96 | 0.33 |  |  |  |
| 1200.5 | 18.58 | 0.08 |  |  |  |
| 1201.6 | 26.87 | 0.20 |  |  |  |
| 1206.6 | 1544.29 | 15.08 |  |  |  |
| 1211.6 | 5630.47 | 41.69 |  |  |  |
| 1218.6 | 39.67 | 0.31 | 1218.6 | 369.21 | 2.72 |
| 1223.6 | 88.96 | 2.22 |  |  |  |
| 1235.6 | 54.90 | 1.08 |  |  |  |
| 1249.6 | 36.60 | 0.17 | 1249.7 | 120.29 | 1.29 |
| 1260.5 | 963.69 | 7.34 |  |  |  |
| 1263.6 | 226.76 | 3.71 |  |  |  |
| 1271.6 | 24.02 | 0.23 |  |  |  |
| 1276.5 | 144.12 | 1.52 | 1276.7 | 59.59 | 0.29 |
| 1282.3 | 33.64 | 0.21 |  |  |  |
| 1293.7 | 60.49 | 0.56 | 1293.6 | 99.84 | 1.13 |
| 1296.5 | 33.13 | 0.14 | 1296.7 | 156.68 | 3.66 |
| 1298.4 | 26.22 | 0.13 |  |  |  |
| 1301.5 | 21.96 | 0.11 |  |  |  |
| 1303.5 | 22.74 | 0.16 | 1303.7 | 70.29 | 0.29 |
| 1306.7 | 69.73 | 0.94 |  |  |  |
| 1324.7 | 42.60 | 0.24 |  |  |  |
| 1331.5 | 36.94 | 0.19 |  |  |  |
| 1336.7 | 247.26 | 10.06 |  |  |  |
| 1347.4 | 70.26 | 0.47 | 1347.7 | 80.39 | 2.36 |
| 1348.7 | 3839.50 | 31.81 |  |  |  |
| 1367.8 | 91.99 | 1.07 |  |  |  |
| 1377.7 | 48.69 | 0.43 | 1377.7 | 92.82 | 0.92 |
| 1389.6 | 33.61 | 0.17 |  |  |  |
| 1393.7 | 74.76 | 0.61 | 1393.7 | 46.01 | 0.18 |
| 1395.7 | 61.03 | 0.47 |  |  |  |
| 1403.7 | 42.15 | 0.19 |  |  |  |
| 1405.7 | 47.42 | 0.28 |  |  |  |
| 1411.4 | 55.92 | 0.44 |  |  |  |
| 1418.6 | 327.69 | 7.44 |  |  |  |
| 1423.8 | 41.97 | 0.61 | 1423.9 | 117.73 | 0.80 |
| 1429.6 | 25.08 | 0.18 | 1429.8 | 48.94 | 0.21 |
| 1434.7 | 94.30 | 0.79 |  |  |  |
| 1440.6 | 28.25 | 0.21 | 1440.8 | 67.21 | 0.40 |
| 1449.7 | 22756.23 | 163.23 |  |  |  |
| 1460.7 | 78.38 | 0.55 | 1460.6 | 71.36 | 0.55 |
| 1464.8 | 159.63 | 2.02 | 1464.8 | 55.08 | 0.32 |
| 1471.7 | 85.78 | 0.60 | 1471.9 | 76.27 | 0.46 |
| 1484.7 | 32.51 | 0.29 |  |  |  |
| 1487.7 | 34.07 | 0.18 | 1487.8 | 79.44 | 0.43 |
| 1489.6 | 26.90 | 0.13 |  |  |  |
| 1494.2 | 22.33 | 0.13 |  |  |  |
| 1498.7 | 63.71 | 2.02 |  |  |  |
| 1504.9 | 114.34 | 1.42 |  |  |  |
| 1511.7 | 24.37 | 0.10 |  |  |  |
| 1516.0 | 58.86 | 0.69 |  |  |  |
| 1518.7 | 114.08 | 0.85 | 1518.8 | 82.49 | 0.43 |
| 1530.9 | 964.01 | 14.23 |  |  |  |
| 1536.6 | 273.51 | 8.78 |  |  |  |
| 1542.7 | 27.68 | 0.19 |  |  |  |
| 1545.5 | 60.17 | 0.39 |  |  |  |
| 1551.7 | 523.50 | 8.92 |  |  |  |
| 1561.6 | 167.70 | 1.65 | 1561.8 | 76.03 | 0.66 |
| 1568.7 | 32.05 | 0.16 |  |  |  |
| 1577.8 | 64.96 | 1.30 |  |  |  |
| 1585.8 | 39.97 | 0.86 |  |  |  |
| 1591.1 | 45.44 | 0.44 |  |  |  |
| 1605.9 | 857.26 | 11.77 | 1605.9 | 3133.56 | 32.22 |
| 1614.7 | 34.08 | 0.23 |  |  |  |
| 1616.6 | 99.17 | 0.97 |  |  |  |
| 1626.8 | 284.00 | 2.58 |  |  |  |
| 1634.8 | 17.62 | 0.09 |  |  |  |
| 1638.8 | 91.25 | 1.74 |  |  |  |
| 1651.6 | 36.47 | 1.83 |  |  |  |
| 1655.8 | 28.01 | 0.21 | 1655.9 | 170.47 | 1.50 |
| 1657.9 | 33.22 | 0.83 |  |  |  |
| 1659.7 | 42.83 | 0.32 | 1659.8 | 65.26 | 0.32 |
| 1664.9 | 79.49 | 2.83 |  |  |  |
| 1678.7 | 83.58 | 0.83 |  |  |  |
| 1690.9 | 4525.61 | 42.67 |  |  |  |
| 1701.8 | 80.91 | 0.78 |  |  |  |
| 1705.7 | 46.44 | 0.36 |  |  |  |
| 1711.8 | 20.03 | 0.38 | 1712.0 | 62.55 | 0.29 |
| 1719.0 | 97.63 | 1.04 |  |  |  |
| 1726.7 | 72.39 | 0.90 |  |  |  |
| 1733.9 | 32.50 | 0.17 | 1734.1 | 113.10 | 0.65 |
| 1739.9 | 618.58 | 5.95 |  |  |  |
| 1751.9 | 324.36 | 13.50 | 1751.9 | 64.63 | 0.26 |
| 1763.0 | 82.71 | 0.49 |  |  |  |
| 1767.0 | 120.70 | 4.88 |  |  |  |
| 1771.8 | 89.22 | 2.08 | 1772.1 | 73.29 | 1.11 |
| 1778.0 | 9258.21 | 82.85 |  |  |  |
| 1786.8 | 268.40 | 3.09 |  |  |  |
| 1796.9 | 24.96 | 0.18 |  |  |  |
| 1799.9 | 33.24 | 0.21 |  |  |  |
| 1818.9 | 45.58 | 0.32 |  |  |  |
| 1821.0 | 72.94 | 0.88 |  |  |  |
| 1826.7 | 45.78 | 0.52 |  |  |  |
| 1832.1 | 38.55 | 0.30 |  |  |  |
| 1835.0 | 53.77 | 0.53 | 1835.2 | 68.06 | 0.45 |
| 1838.9 | 59.65 | 0.69 |  |  |  |
| 1847.0 | 189.26 | 3.03 |  |  |  |
| 1850.3 | 379.28 | 5.53 |  |  |  |
| 1853.9 | 47.63 | 0.47 | 1854 | 54.43 | 0.27 |
| 1865.0 | 32022.00 | 312.38 |  |  |  |
| 1881.1 | 179.85 | 1.37 |  |  |  |
| 1886.0 | 55.76 | 1.00 |  |  |  |
| 1895.9 | 1775.54 | 17.07 |  |  |  |
| 1902.9 | 29.90 | 0.20 |  |  |  |
| 1913.8 | 64.10 | 0.78 |  |  |  |
| 1919.2 | 55.58 | 0.64 | 1919.0 | 43.55 | 0.24 |
| 1928.0 | 52.92 | 0.75 |  |  |  |
| 1934.1 | 557.48 | 10.06 |  |  |  |
| 1943.9 | 2421.50 | 19.66 |  |  |  |
| 1953.0 | 32.76 | 0.32 |  |  |  |
| 1968.9 | 33.82 | 0.49 |  |  |  |
| 1977.1 | 26.92 | 0.32 |  |  |  |
| 1980.2 | 28.74 | 0.28 |  |  |  |
| 1984.5 | 118.98 | 2.70 |  |  |  |
| 1994.9 | 29.58 | 0.38 |  |  |  |
| 2006.3 | 601.66 | 13.68 |  |  |  |
| 2011.0 | 613.17 | 5.34 | 2011.3 | 66.83 | 0.34 |
| 2021.0 | 5382.80 | 102.24 |  |  |  |
| 2027.9 | 291.67 | 2.29 |  |  |  |
| 2037.1 | 31.30 | 0.29 |  |  |  |
| 2043.0 | 32.77 | 0.23 |  |  |  |
| 2052.0 | 35.26 | 0.20 |  |  |  |
| 2069.9 | 69.46 | 0.48 |  |  |  |
| 2081.1 | 666.17 | 3.99 |  |  |  |
| 2109.1 | 33.70 | 0.17 |  |  |  |
| 2116.0 | 237.61 | 3.87 |  |  |  |
| 2127.1 | 181.91 | 1.72 |  |  |  |
| 2152.9 | 50.03 | 0.28 |  |  |  |
| 2167.0 | 30.74 | 0.20 |  |  |  |
| 2180.1 | 21.58 | 0.10 | 2180.2 | 43.93 | 0.21 |
| 2184.1 | 77.28 | 1.37 |  |  |  |
| 2209.2 | 928.38 | 6.99 |  |  |  |
| 2215.1 | 20.01 | 0.14 |  |  |  |
| 2228.9 | 35.31 | 0.23 | 2229.1 | 174.51 | 3.42 |
| 2231.9 | 26.29 | 0.15 |  |  |  |
| 2238.1 | 21.87 | 0.12 |  |  |  |
| 2247.9 | 16.60 | 0.10 |  |  |  |
| 2251.1 | 20.85 | 0.12 |  |  |  |
| 2256.1 | 24.77 | 0.79 |  |  |  |
| 2267.0 | 95.58 | 1.69 |  |  |  |
| 2271.1 | 387.11 | 4.41 |  |  |  |
| 2287.0 | 17.79 | 0.10 |  |  |  |
| 2292.0 | 18.80 | 0.14 |  |  |  |
| 2305.1 | 22.98 | 0.16 | 2305 | 43.51 | 0.22 |
| 2310.1 | 16.67 | 0.13 | 2310.2 | 42.27 | 0.25 |
| 2337.0 | 29.31 | 0.30 |  |  |  |
| 2358.1 | 51.66 | 0.72 |  |  |  |
| 2394.2 | 16.24 | 0.17 |  |  |  |
| 2409.2 | 64.37 | 1.07 |  |  |  |
| 2451.2 | 172.17 | 13.43 |  |  |  |
| 2542.3 | 16.91 | 0.14 | 2542.4 | 50.63 | 1.21 |
| 2551.1 | 35.50 | 0.47 |  |  |  |
| 2554.0 | 494.79 | 4.09 | 2554.1 | 30.02 | 0.15 |
| 2565.3 | 14.91 | 0.23 | 2565.3 | 231.37 | 1.85 |
| 2583.2 | 28.99 | 0.19 | 2583.4 | 100.36 | 1.33 |
| 2599.1 | 28.65 | 0.54 |  |  |  |
| 2603.2 | 68.04 | 0.41 |  |  |  |
| 2616.3 | 13.40 | 0.11 |  |  |  |
| 2627.3 | 16.78 | 0.14 |  |  |  |
| 2645.3 | 10.17 | 0.20 | 2645.4 | 34.95 | 0.15 |
| 2691.4 | 9.46 | 0.14 |  |  |  |
| 2724.3 | 55.57 | 0.44 |  |  |  |
| 2752.4 | 18.47 | 0.09 |  |  |  |
| 2755.4 | 62.12 | 0.88 |  |  |  |
| 2762.2 | 7.66 | 0.07 |  |  |  |
| 2816.3 | 19.55 | 0.12 |  |  |  |
| 2858.5 | 7.20 | 0.05 |  |  |  |
| 2931.2 | 48.20 | 0.48 |  |  |  |
| 3141.5 | 8.89 | 0.03 |  |  |  |
| 3156.6 | 57.34 | 0.52 |  |  |  |
| 3272.6 | 19.21 | 0.11 |  |  |  |
| 3681.0 | 5.87 | 0.03 |  |  |  |
