# Supplementary material for: MALDI-TOF peptidomic analysis of serum and post-prostatic massage urine specimens to identify prostate cancer biomarkers
Source: Clin Proteomics. 2018 Jul 25;15:23. doi: 10.1186/s12014-018-9199-8 (PMC6060548; doi:10.1186/s12014-018-9199-8)
Supplement: Supplementary file 13 — Additional file 13: Table S4. The significant MS-MS fragmentation patterns of the serum features analyzed using MALDI-TOF/MS set at CID conditions. [file 12014_2018_9199_MOESM13_ESM.doc]

**Supplementary Table 4:** The significant MS-MS fragmentation patterns of the serum features analyzed using MALDI-TOF/MS set at CID conditions.

| **m/z** | **Score** | **MS-Tag derived Sequence** | **Uniprot Accession number** | **Candidate protein Name** |
| --- | --- | --- | --- | --- |
| 1739.9 | 44.2 | (R)NGFKSHALQLNNRQINGFKSHALQLNNRQI(R) | P0C0L4 | Complement C4-A |
| 1978.1 | 31.3 | (Q)AGSQHGQSESIVPERHGTTAGSQHGQSESIVPERHGTT(H) | Q5D862 | Filaggrin-2 |
| 1020.5 | 30.2 | (G)DFLAEGGGVRDFLAEGGGVR(G) | P02671 | Fibrinogen alpha chain |
| 1896.0 | 29.8 | (R)NGFKSHALQLNNRQIRNGFKSHALQLNNRQIR(G) | P0C0L4 | Complement C4-A |
| 1419.3 | 29.4 | (P)PRPPATSTAASPLGPPRPPATSTAASPLGP(L) | E7ERA6 | RING finger protein 223 |
| 1192.4 | 29.3 | (A)RVMLPPGAQHS(D) | P55196 | Afadin |
| 1605.8 | 28.4 | (I)THRIHWESASLLRTHRIHWESASLLR(S) | P01024 | Complement C3 |
| 1887 | 26.1 | (Y)EVRFHWGRENQRGSE(H) | P35219 | Carbonic anhydrase-related protein |
| 1847 | 24.1 | (A)TFRLKDGVLAYARLSHTFRLKDGVLAYARLSH(L) | Q14690 | Protein RRP5 homolog |
| 3156.3 | 23.4 | (T)VASSKPSSARKTPESFLGPNAALVNLDSLVT(R) | O95208 | Epsin-2 |
| 1504.7 | 23.3 | (G)GGGGSGASSGGGAGGLQPSSGGGGSGASSGGGAGGLQPSS(R) | Q9ULU8 | Calcium-dependent secretion activator 1 |
| 1367.7 | 23.2 | (Y)HRLKEGAVPTIFHRLKEGAVPTIF(E) | Q9BT49 | THAP domain-containing protein 7 |
