# Supplementary material for: MALDI-TOF peptidomic analysis of serum and post-prostatic massage urine specimens to identify prostate cancer biomarkers
Source: Clin Proteomics. 2018 Jul 25;15:23. doi: 10.1186/s12014-018-9199-8 (PMC6060548; doi:10.1186/s12014-018-9199-8)
Supplement: Supplementary file 14 — Additional file 14: MS-Tag search results. MS-MS spectra, peptide lists and MS-Tag search results (including all the configuration parameter) for the fragmentation patters of the 12 MALDI-TOF/MS serum features. [file 12014_2018_9199_MOESM14_ESM.zip › New folder/1020_5.pdf]

MS-Tag Search Results

Search completed. 12 sec elapsed. 0 sec remaining.

[–] **Parameters**

Database searched: **SwissProt.2016.5.30**
Digest Used: **No enzyme**
Max. # Missed Cleavages: **1**
Constant Modification: **Carbamidomethyl (C)**
Ion Types Considered: **a, a-NH3, a-H2O, b, b-NH3, b-H2O, b+H2O, y, y-NH3, y-H2O, I, i, P, S, M-H2O, M-NH3, M-SOCH4**
Search Mode:
Max Modifications: **2**
Peptide Masses are: **monoisotopic**

[–] **Pre Search Results (SwissProt.2016.5.30)**

Number of entries in the database: **551193**
Full Molecular Weight range: **551193** entries.
Full pI range: **551193** entries.
Taxonomy search **HOMO SAPIENS** selects **20202** entries.
Pre searches select **20202** entries.

Results

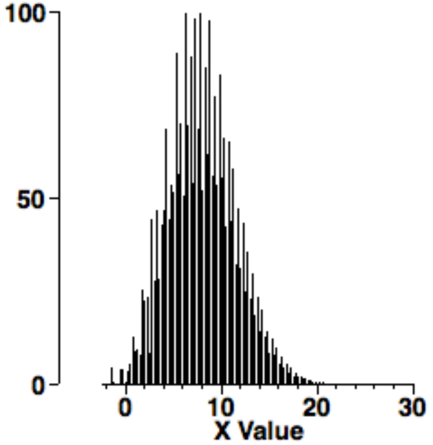

<< < > >> >< <> All

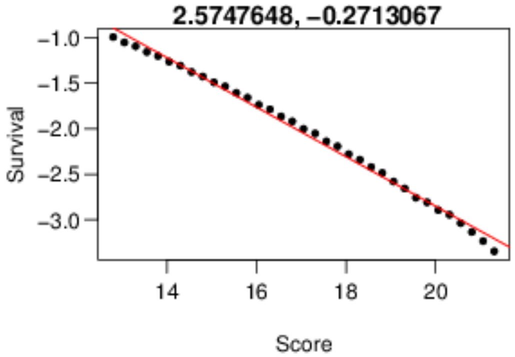

expectation value = 0.288
num peptides considered = 119649
MS-Tag search selects **31** entries (results displayed for top **30** matches).

Parent mass: **1020.5000 (+/- 0.500 Da)**
[–] **Fragment Ions**

26 Ions used in search: **59.1000, 72.1000, 72.1000, 86.1000, 102.1000, 112.1000, 115.1000, 120.1000, 129.2000, 157.2000, 158.1000, 175.1000, 201.1000, 235.2000, 243.2000, 257.2000, 263.1000, 314.2000, 371.3000, 376.2000, 388.3000, 428.3000, 445.3000, 447.3000, 905.8000, 959.8000 (+/- 1.00 Da)**

Rank	# Unmatched Ions	Sequence	Score	Expect	MH^+ Calculated (Da)	Error (Da)	Protein MW (Da)/pI	Accession #	Species	Protein Name
1	**2**	**(G)DFLAEGGGVRDFLAEGGGVR(G)**	**30.2**	**0.29**	**1020.5109**	**-0.0109**	**94974/5.7**	**P02671 P02671**	HUMAN	Fibrinogen alpha chain
2	**5**	**(A)NSSVGKWQDNSSVGKWQD(R)**	**26.1**	**3.7**	**1020.4745**	**0.0255**	**66527/5.4**	**Q6UN15 Q6UN15**	HUMAN	Pre-mRNA 3'-end-processing factor FIP1
3	**6**	**(F)DFPGSAGVDRDFPGSAGVDR(C)**	**25.0**	**7.4**	**1020.4745**	**0.0255**	**136002/6.2**	**Q10571 Q10571**	HUMAN	Transcriptional activator MN1
4	**8**	**(S)LSASSKALFPLSASSKALFP(S)**	**24.7**	**8.9**	**1020.5724**	**-0.0724**	**50751/9.2**	**O43670 O43670**	HUMAN	BUB3-interacting and GLEBS motif-containing protein ZNF207
5	**5**	**(P)VSGAEMGTLRVSGAEMGTLR(R)**	**24.2**	**12**	**1020.5143**	**-0.0143**	**136387/5.5**	**Q9P2M7 Q9P2M7**	HUMAN	Cingulin
6	**9**	**(S)GTVLGM(Oxidation)ALRSGTVLGM(Oxidation)ALRS(H)**	**23.9**	**15**	**1020.5506**	**-0.0506**	**30792/8.3**	**Q8N8Q3 Q8N8Q3**	HUMAN	Endonuclease V
7	**5**	**(D)NFDIAEGVRNFDIAEGVR(T)**	**23.8**	**16**	**1020.5109**	**-0.0109**	**82431/8.5**	**P29728 P29728**	HUMAN	2'-5'-oligoadenylate synthase 2
8	**6**	**(Q)ESTAGVTISRESTAGVTISR(V)**	**23.6**	**18**	**1020.5320**	**-0.0320**	**240122/8.0**	**Q86YW9 Q86YW9**	HUMAN	Mediator of RNA polymerase II transcription subunit 12-like protein

9	**8**	**(L)PC(Carbamidomethyl)TSKAIM(Oxidation)PPC(Carbamidomethyl)TSKAIM(Oxidation)P(Y)**	**23.5**	**19**	**1020.4853**	**0.0147**	**82237/7.2**	**Q9NVR2**	**Q9NVR2**	HUMAN	Integrator complex subunit 10
10	**5**	**(M)AAM(Oxidation)AVGGAGGSRAAM(Oxidation)AVGGAGGSR(V)**	**23.4**	**20**	**1020.4891**	**0.0109**	**72685/5.9**	**O14744**	**O14744**	HUMAN	Protein arginine N-methyltransferase 5
11	**6**	**(I)SFNVQNGVRSFNVQNGVR(A)**	**23.3**	**21**	**1020.5221**	**-0.0221**	**63267/9.1**	**Q8NDF8**	**Q8NDF8**	HUMAN	Non-canonical poly(A) RNA polymerase PAPD5
12	**8**	**(R)DPFSQALSRDPFSQALSR(C)**	**23.2**	**23**	**1020.5109**	**-0.0109**	**52568/7.1**	**Q96DF8**	**Q96DF8**	HUMAN	Protein DGCR14
12	**6**	**(A)GGFGKASGALVGGGFGKASGALVG(A)**	**23.2**	**23**	**1020.5473**	**-0.0473**	**39473/7.5**	**Q9Y5R6**	**Q9Y5R6**	HUMAN	Doublesex- and mab-3-related transcription factor 1
13	**8**	**(A)AVSGM(Oxidation)IAISRAVSGM(Oxidation)IAISR(M)**	**23.1**	**24**	**1020.5506**	**-0.0506**	**46427/4.8**	**Q99574**	**Q99574**	HUMAN	Neuroserpin
14	**8**	**(N)SSSNC(Carbamidomethyl)NRVPSSSNC(Carbamidomethyl)NRVP(D)**	**23.0**	**26**	**1020.4527**	**0.0473**	**73769/6.5**	**Q92611**	**Q92611**	HUMAN	ER degradation-enhancing alpha-mannosidase-like protein 1
15	**7**	**(N)SSVSGNILFPSSVSGNILFP(V)**	**22.9**	**28**	**1020.5360**	**-0.0360**	**138862/5.8**	**Q96QP1**	**Q96QP1**	HUMAN	Alpha-protein kinase 1
16	**9**	**(K)KKMAADIFPKKMAADIFP(R)**	**22.8**	**29**	**1020.5547**	**-0.0547**	**58420/7.4**	**Q9Y2F9**	**Q9Y2F9**	HUMAN	BTB/POZ domain-containing protein 3
17	**7**	**(S)DM(Oxidation)LAAGGKIEDM(Oxidation)LAAGGKIE(S)**	**22.5**	**35**	**1020.5030**	**-0.00302**	**129932/4.4**	**Q9NQC3**	**Q9NQC3**	HUMAN	Reticulon-4
17	**7**	**(A)GGGAQDESGSRGGGAQDESGSR(I)**	**22.5**	**35**	**1020.4341**	**0.0659**	**78637/8.8**	**Q2TAK8**	**Q2TAK8**	HUMAN	PWWP domain-containing protein MUM1
18	**10**	**(E)SSSSVKTDLPSSSSVKTDLP(Q)**	**22.4**	**38**	**1020.5208**	**-0.0208**	**71344/5.5**	**Q9H6X5**	**Q9H6X5**	HUMAN	Uncharacterized protein C19orf44
19	**6**	**(N)SSELGVFQGPSSELGVFQGP(D)**	**22.3**	**40**	**1020.4997**	**3.48e-4**	**57167/9.3**	**Q9NZ20**	**Q9NZ20**	HUMAN	Group 3 secretory phospholipase A2
20	**8**	**(S)KAGMSGARSRKAGMSGARSR(N)**	**22.2**	**43**	**1020.5367**	**-0.0367**	**79213/6.4**	**Q8N8K9**	**Q8N8K9**	HUMAN	Uncharacterized protein KIAA1958
20	**9**	**(N)SSSKRVIFPSSSKRVIFP(R)**	**22.2**	**43**	**1020.5837**	**-0.0837**	**57876/8.4**	**Q8N8W4**	**Q8N8W4**	HUMAN	Patatin-like phospholipase domain-containing protein 1
											Mitogen-

21	**8**	**(L)VKC(Carbamidomethyl)ADANSRVKC(Carbamidomethyl)ADANSR(T)**	**22.1**	**45**	**1020.4891**	**0.0109**	**164471/7.9**	**Q13233 Q13233**	HUMAN	activated protein kinase kinase kinase 1
21	**10**	**(G)KQGGM(Oxidation)DGSKPKQGGM(Oxidation)DGSKP(A)**	**22.1**	**45**	**1020.4779**	**0.0221**	**31858/6.5**	**Q96DX5 Q96DX5**	HUMAN	Ankyrin repeat and SOCS box protein 9
21	**10**	**(N)KNADSELM(Oxidation)PKNADSELM(Oxidation)P(P)**	**22.1**	**45**	**1020.4666**	**0.0334**	**59310/4.5**	**Q9BUR4 Q9BUR4**	HUMAN	Telomerase Cajal body protein 1
22	**9**	**(V)TSVTIAANM(Oxidation)PTSVTIAANM(Oxidation)P(S)**	**22.0**	**48**	**1020.5030**	**-0.00302**	**63342/7.4**	**O15405 O15405**	HUMAN	TOX high mobility group box family member 3
22	**7**	**(T)GTVIDSGDGVTGTVIDSGDGVT(H)**	**22.0**	**48**	**1020.4844**	**0.0156**	**47372/5.6**	**P61158 P61158**	HUMAN	Actin-related protein 3
22	**8**	**(L)SAVMGVTLTAASAVMGVTLTAA(I)**	**22.0**	**48**	**1020.5394**	**-0.0394**	**113897/8.3**	**Q13423 Q13423**	HUMAN	NAD(P) transhydrogenase, mitochondrial
23	**8**	**(K)DSENKAIFPDSENKAIFP(D)**	**21.9**	**51**	**1020.4997**	**3.48e-4**	**40105/5.6**	**Q8NDY3 Q8NDY3**	HUMAN	[Protein ADP-ribosylarginine] hydrolase-like protein 1
