# Supplementary material for: MALDI-TOF peptidomic analysis of serum and post-prostatic massage urine specimens to identify prostate cancer biomarkers
Source: Clin Proteomics. 2018 Jul 25;15:23. doi: 10.1186/s12014-018-9199-8 (PMC6060548; doi:10.1186/s12014-018-9199-8)
Supplement: Supplementary file 14 — Additional file 14: MS-Tag search results. MS-MS spectra, peptide lists and MS-Tag search results (including all the configuration parameter) for the fragmentation patters of the 12 MALDI-TOF/MS serum features. [file 12014_2018_9199_MOESM14_ESM.zip › New folder/1192_6.pdf]

MS-Tag Search Results

Search completed. 13 sec elapsed. 0 sec remaining.

[–] **Parameters**

Database searched: **SwissProt.2016.5.30**
Digest Used: **No enzyme**
Max. # Missed Cleavages: **1**
Constant Modification: **Carbamidomethyl (C)**
Ion Types Considered: **a, a-NH3, a-H2O, b, b-NH3, b-H2O, b+H2O, y, y-NH3, y-H2O, I, i, P, S, M-H2O, M-NH3, M-SOCH4**
Search Mode:
Max Modifications: **2**
Peptide Masses are: **monoisotopic**

[–] **Pre Search Results (SwissProt.2016.5.30)**

Number of entries in the database: **551193**
Full Molecular Weight range: **551193** entries.
Full pI range: **551193** entries.
Taxonomy search **HOMO SAPIENS** selects **20202** entries.
Pre searches select **20202** entries.

Results

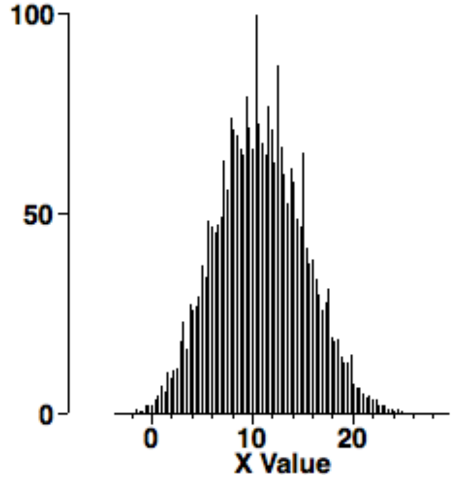

<< < > >> >< <> All

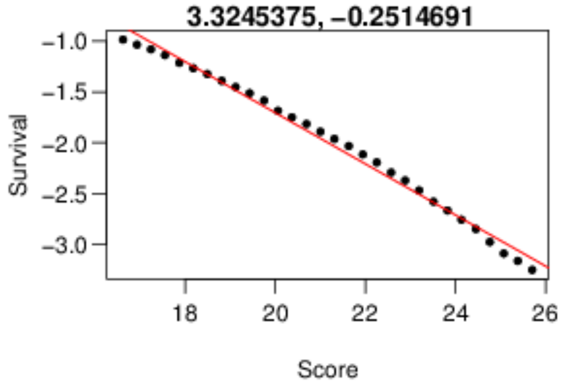

expectation value = 10.8
num peptides considered = 119233
MS-Tag search selects **31** entries (results displayed for top **30** matches).

Parent mass: **1192.4000 (+/- 0.500 Da)**
[–] **Fragment Ions**

50 Ions used in search: **23.0000, 39.0000, 44.1000, 69.6000, 70.1000, 72.1000, 84.1000, 86.1000, 87.1000, 95.1000, 104.1000, 110.1000, 111.1000, 112.1000, 120.1000, 121.1000, 129.1000, 154.1000, 170.1000, 171.1000, 171.9000, 175.1000, 184.1000, 195.1000, 197.1000, 239.1000, 249.2000, 253.1000, 294.2000, 296.1000, 359.2000, 371.2000, 372.1000, 403.1000, 409.2000, 445.2000, 575.4000, 695.4000, 706.3000, 762.3000, 777.4000, 901.3000, 905.6000, 918.5000, 940.5000, 1036.6000, 1046.6000, 1064.7000, 1090.4000, 1108.8000 (+/- 1.00 Da)**

Rank	# Unmatched	Sequence	Score	Expect	MH$^+$ Calculated	Error	Protein MW	Accession	Species	Protein Name

	Ions				(Da)	(Da)	(Da)/pI	#		
1	**20**	**(A)RVMLPPGAQHSRVMLPPGAQHS(D)**	**29.3**	**11**	**1192.6255**	**-0.226**	**206806/6.1**	**P55196 P55196**	HUMAN	Afadin
2	**25**	**(P)QVFSQMTPVRQVFSQMTPVR(P)**	**28.7**	**15**	**1192.6143**	**-0.214**	**155345/7.1**	**Q7Z3K3 Q7Z3K3**	HUMAN	Pogo transposable element with ZNF domain
3	**25**	**(P)RSTSM(Oxidation)QDPVRRSTSM(Oxidation)QDPVR(E)**	**28.5**	**17**	**1192.5739**	**-0.174**	**184669/9.0**	**Q9BYB0 Q9BYB0**	HUMAN	SH3 and multiple ankyrin repeat domains protein 3
4	**25**	**(L)QELFGRVTSRQELFGRVTSR(V)**	**28.4**	**18**	**1192.6433**	**-0.243**	**96633/5.4**	**Q6P3X3 Q6P3X3**	HUMAN	Tetratricopeptide repeat protein 27
5	**25**	**(V)RMAPSYVLKKRMAPSYVLKK(A)**	**28.2**	**20**	**1192.6871**	**-0.287**	**102004/5.7**	**Q9Y2K6 Q9Y2K6**	HUMAN	Ubiquitin carboxyl-terminal hydrolase 20
6	**25**	**(L)IGTFRMVLQKIGTFRMVLQK(V)**	**28.0**	**23**	**1192.6871**	**-0.287**	**226755/5.5**	**Q9HC10 Q9HC10**	HUMAN	Otoferlin
6	**24**	**(N)AGQVVHLTLVRAGQVVHLTLVR(R)**	**28.0**	**23**	**1192.7161**	**-0.316**	**196370/4.8**	**Q8NI35 Q8NI35**	HUMAN	InaD-like protein
7	**24**	**(L)KFKGPM(Oxidation)TVLRKFKGPM(Oxidation)TVLR(L)**	**27.9**	**24**	**1192.6871**	**-0.287**	**51802/9.3**	**P14867 P14867**	HUMAN	Gamma-aminobutyric acid receptor subunit alpha-1
8	**25**	**(P)RLTPNMINSFRLTPNMINSF(V)**	**27.7**	**27**	**1192.6143**	**-0.214**	**65323/9.2**	**Q96NX9 Q96NX9**	HUMAN	Dachshund homolog 2
8	**26**	**(L)SKAEFVEKVRSKAEFVEKVR(Q)**	**27.7**	**27**	**1192.6684**	**-0.268**	**270887/6.4**	**Q96AY4 Q96AY4**	HUMAN	Tetratricopeptide repeat protein 28
9	**25**	**(R)VPGGPPASNLRKVPGGPPASNLRK(Q)**	**27.6**	**29**	**1192.6797**	**-0.280**	**202473/8.2**	**Q8NEY1 Q8NEY1**	HUMAN	Neuron navigator 1
10	**23**	**(S)RHHNSTAELQRHHNSTAELQ(K)**	**27.3**	**34**	**1192.5818**	**-0.182**	**171593/6.7**	**P33527 P33527**	HUMAN	Multidrug resistance-associated protein 1
10	**26**	**(D)RLTSQLEAFQRLTSQLEAFQ(A)**	**27.3**	**34**	**1192.6321**	**-0.232**	**34797/5.6**	**A6NI79 A6NI79**	HUMAN	Coiled-coil domain-containing protein 69
10	**23**	**(I)RPSTLHGLSPKRPSTLHGLSPK(L)**	**27.3**	**34**	**1192.6797**	**-0.280**	**170678/8.7**	**Q9Y2H9 Q9Y2H9**	HUMAN	Microtubule-associated serine/threonine-protein kinase 1
11	**22**	**(W)QSPDIHQVRIQSPDIHQVRI(P)**	**27.2**	**36**	**1192.6433**	**-0.243**	**49976/6.1**	**Q9Y2G5 Q9Y2G5**	HUMAN	GDP-fucose protein O-fucosyltransferase

										2
11	**24**	**(G)RTAPLGQPPGAQRTAPLGQPPGAQ(L)**	**27.2**	**36**	**1192.6433**	**-0.243**	**294654/7.4**	**Q9BYK8 Q9BYK8**	HUMAN	Helicase with zinc finger domain 2
12	**25**	**(P)KALTAGQNRPHKALTAGQNRPH(P)**	**27.1**	**39**	**1192.6545**	**-0.255**	**34263/6.0**	**Q7Z7C8 Q7Z7C8**	HUMAN	Transcription initiation factor TFIID subunit 8
13	**23**	**(L)RLLSMFGLKQRLLSMFGLKQ(R)**	**26.8**	**46**	**1192.6871**	**-0.287**	**44702/9.1**	**P12643 P12643**	HUMAN	Bone morphogenetic protein 2
14	**29**	**(D)AGFLEMKGALRAGFLEMKGALR(E)**	**26.7**	**49**	**1192.6507**	**-0.251**	**121051/8.7**	**Q9BQI6 Q9BQI6**	HUMAN	SMC5-SMC6 complex localization factor protein 1
14	**27**	**(K)KSSVKSGSREKKSSVKSGSREK(Q)**	**26.7**	**49**	**1192.6644**	**-0.264**	**51467/9.8**	**O95232 O95232**	HUMAN	Luc7-like protein 3
14	**24**	**(I)SSRGSTVRSKKSSRGSTVRSKK(S)**	**26.7**	**49**	**1192.6757**	**-0.276**	**141328/5.3**	**Q8N3U4 Q8N3U4**	HUMAN	Cohesin subunit SA-2
15	**23**	**(A)GSLGTAGRVC(Carbamidomethyl)SKGSLGTAGRVC(Carbamidomethyl)SK(T)**	**26.6**	**51**	**1192.6103**	**-0.210**	**43770/9.3**	**Q93097 Q93097**	HUMAN	Protein Wnt-2b
15	**27**	**(L)KENADFLSLRKENADFLSLR(Q)**	**26.6**	**51**	**1192.6321**	**-0.232**	**235654/6.6**	**Q6UB98 Q6UB98**	HUMAN	Ankyrin repeat domain-containing protein 12
15	**23**	**(P)RLLSFTSQLKRLLSFTSQLK(A)**	**26.6**	**51**	**1192.7048**	**-0.305**	**119107/6.3**	**Q9Y666 Q9Y666**	HUMAN	Solute carrier family 12 member 7
16	**26**	**(R)KADGGEM(Oxidation)TVIRKADGGEM(Oxidation)TVIR(S)**	**26.5**	**55**	**1192.5990**	**-0.199**	**521963/4.9**	**P98164 P98164**	HUMAN	Low-density lipoprotein receptor-related protein 2
16	**25**	**(S)QETPLHVAAARQETPLHVAAAR(G)**	**26.5**	**55**	**1192.6433**	**-0.243**	**49637/8.4**	**Q96NS5 Q96NS5**	HUMAN	Ankyrin repeat and SOCS box protein 16
16	**25**	**(P)GRPSVIPDHSKGRPSVIPDHSK(K)**	**26.5**	**55**	**1192.6433**	**-0.243**	**122653/9.7**	**Q9P275 Q9P275**	HUMAN	Ubiquitin carboxyl-terminal hydrolase 36
17	**25**	**(M)RHMATHSPQKRHMATHSPQK(S)**	**26.4**	**58**	**1192.6004**	**-0.200**	**50820/8.8**	**Q9UM63 Q9UM63**	HUMAN	Zinc finger protein PLAGL1
17	**26**	**(A)KVSTVMDTVGRKVSTVMDTVGR(R)**	**26.4**	**58**	**1192.6354**	**-0.235**	**68761/8.2**	**Q9H845 Q9H845**	HUMAN	Acyl-CoA dehydrogenase family member 9,

										mitochondrial
17	**25**	**(T)KPSSSVTPRHPKPSSSVTPRHP(L)**	**26.4**	**58**	**1192.6433**	**-0.243**	**110539/7.0**	**Q8IWR0**	**Q8IWR0** HUMAN	Zinc finger CCCH domain-containing protein 7A
