# Supplementary material for: MALDI-TOF peptidomic analysis of serum and post-prostatic massage urine specimens to identify prostate cancer biomarkers
Source: Clin Proteomics. 2018 Jul 25;15:23. doi: 10.1186/s12014-018-9199-8 (PMC6060548; doi:10.1186/s12014-018-9199-8)
Supplement: Supplementary file 14 — Additional file 14: MS-Tag search results. MS-MS spectra, peptide lists and MS-Tag search results (including all the configuration parameter) for the fragmentation patters of the 12 MALDI-TOF/MS serum features. [file 12014_2018_9199_MOESM14_ESM.zip › New folder/1367_7.pdf]

MS-Tag Search Results

Search completed. 12 sec elapsed. 0 sec remaining.

[–] **Parameters**

Database searched: **SwissProt.2016.5.30**
Digest Used: **No enzyme**
Max. # Missed Cleavages: **1**
Constant Modification: **Carbamidomethyl (C)**
Ion Types Considered: **a, a-NH3, a-H2O, b, b-NH3, b-H2O, b+H2O, y, y-NH3, y-H2O, I, i, P, S, M-H2O, M-NH3, M-SOCH4**
Search Mode:
Max Modifications: **2**
Peptide Masses are: **monoisotopic**

[–] **Pre Search Results (SwissProt.2016.5.30)**

Number of entries in the database: **551193**
Full Molecular Weight range: **551193** entries.
Full pI range: **551193** entries.
Taxonomy search **HOMO SAPIENS** selects **20202** entries.
Pre searches select **20202** entries.

Results

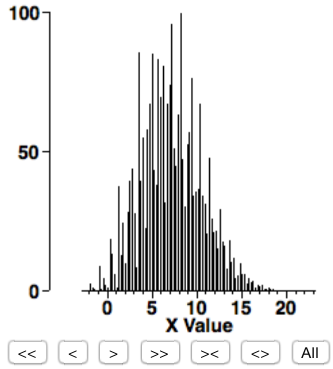

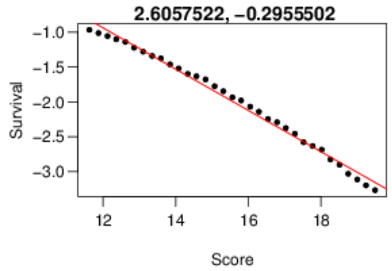

expectation value = 6.18
num peptides considered = 117801
MS-Tag search selects **31** entries (results displayed for top **30** matches).

Parent mass: **1367.7000 (+/- 0.500 Da)**
[–] **Fragment Ions**

30 Ions used in search: **23.0000, 39.0000, 60.1000, 70.1000, 86.1000, 110.1000, 111.1000, 112.1000, 113.1000, 115.1000, 130.1000, 157.1000, 159.1000, 166.1000, 169.1000, 170.1000, 175.1000, 225.2000, 296.2000, 324.2000, 362.3000, 379.3000, 390.3000, 646.4000, 722.4000, 961.7000, 1212.0000, 1308.1000, 1326.1000, 1351.2000 (+/- 1.00 Da)**

Rank	# Unmatched Ions	Sequence	Score	Expect	MH^+ Calculated (Da)	Error (Da)	Protein MW (Da)/pI	Accession #	Species	Protein Name
1	**11**	**(Y)HRLKEGAVPTIFHRLKEGAVPTIF(E)**	**23.3**	**6.2**	**1367.7794**	**-0.0794**	**34414/9.5**	**Q9BT49 Q9BT49**	HUMAN	THAP domain-containing protein 7
2	**10**	**(H)RIHWESASLLRRIHWESASLLR(S)**	**23.1**	**7.1**	**1367.7542**	**-0.0542**	**187150/6.0**	**P01024 P01024**	HUMAN	Complement C3
3	**10**	**(Y)HLEEGGVSKKQRHLEEGGVSKKQR(T)**	**23.0**	**7.6**	**1367.7390**	**-0.0390**	**71423/5.1**	**P52739 P52739**	HUMAN	Zinc finger protein 131
4	**12**	**(S)VGRVSFPSLVC(Carbamidomethyl)FVGRVSFPSLVC(Carbamidomethyl)F(L)**	**22.6**	**9.9**	**1367.7140**	**-0.0140**	**76697/7.0**	**Q6MZZ7 Q6MZZ7**	HUMAN	Calpain-13
5	**11**	**(S)PAPPEGPSHPREPPAPPEGPSHPREP(S)**	**22.5**	**11**	**1367.6702**	**0.0298**	**231433/6.2**	**O14578 O14578**	HUMAN	Citron Rho-interacting kinase
6	**13**	**(G)HLQAVPELRHAPHLQAVPELRHAP(D)**	**21.8**	**17**	**1367.7542**	**-0.0542**	**41366/5.1**	**Q969T3 Q969T3**	HUMAN	Sorting nexin-21
7	**10**	**(Q)RAAKNGPPVSDQKRAAKNGPPVSDQK(E)**	**21.4**	**23**	**1367.7390**	**-0.0390**	**100147/6.9**	**Q13469 Q13469**	HUMAN	Nuclear factor of activated T-cells,

										cytoplasmic 2
8	**11**	**(L)HSAISVPISSDASPHSAISVPISSDASP(F)**	**21.3**	**24**	**1367.6801**	**0.0199**	**190361/5.7**	**Q7Z3U7 Q7Z3U7**	HUMAN	Protein MON2 homolog
8	**11**	**(K)RLHSADISPQDERLHSADISPQDE(Q)**	**21.3**	**24**	**1367.6550**	**0.0450**	**71664/8.0**	**Q9NQV8 Q9NQV8**	HUMAN	PR domain zinc finger protein 8
9	**11**	**(L)VGSSPHREASPM(Oxidation)PVGSSPHREASPM(Oxidation)P(G)**	**21.2**	**26**	**1367.6372**	**0.0628**	**248622/4.9**	**Q3T8J9 Q3T8J9**	HUMAN	GON-4-like protein
9	**12**	**(R)GKFLLSTLEGFRGKFLLSTLEGFR(S)**	**21.2**	**26**	**1367.7682**	**-0.0682**	**141163/6.2**	**Q5TIA1 Q5TIA1**	HUMAN	Meiosis inhibitor protein 1
10	**12**	**(S)FM(Oxidation)EGGALRVSERFM(Oxidation)EGGALRVSER(T)**	**21.1**	**28**	**1367.6736**	**0.0264**	**96624/6.0**	**Q9H8M5 Q9H8M5**	HUMAN	Metal transporter CNNM2
10	**10**	**(K)RSSSLGGSTGSTPSSRSSSLGGSTGSTPSS(S)**	**21.1**	**28**	**1367.6397**	**0.0603**	**120675/5.7**	**P41180 P41180**	HUMAN	Extracellular calcium-sensing receptor
11	**12**	**(H)ASSESPSTIKLTM(Oxidation)ASSESPSTIKLTM(Oxidation)(A)**	**21.0**	**30**	**1367.6723**	**0.0277**	**1519187/5.1**	**Q8WXI7 Q8WXI7**	HUMAN	Mucin-16
11	**11**	**(L)HDAAAAADSPAGTRGHDAAAAADSPAGTRG(G)**	**21.0**	**30**	**1367.6298**	**0.0702**	**71687/9.4**	**Q9HDC5 Q9HDC5**	HUMAN	Junctophilin-1
11	**11**	**(E)ASLKQKGHKSQRASLKQKGHKSQR(E)**	**21.0**	**30**	**1367.7866**	**-0.0866**	**229834/5.8**	**Q6GYQ0 Q6GYQ0**	HUMAN	Ral GTPase-activating protein subunit alpha-1
12	**12**	**(Q)SAQAMASRHRPRSAQAMASRHRPR(P)**	**20.9**	**32**	**1367.7073**	**-0.00731**	**23406/10.7**	**Q9NTK1 Q9NTK1**	HUMAN	Protein DEPP
12	**10**	**(L)GTSTLQRHLQARGTSTLQRHLQAR(H)**	**20.9**	**32**	**1367.7502**	**-0.0502**	**109974/6.1**	**P86452 P86452**	HUMAN	Zinc finger BED domain-containing protein 6
13	**11**	**(A)GAPSRASPGVPSERGAPSRASPGVPSER(T)**	**20.8**	**34**	**1367.7026**	**-0.00260**	**186798/5.6**	**Q14766 Q14766**	HUMAN	Latent-transforming growth factor beta-binding protein 1
13	**12**	**(G)GRKPWPQKGTGRGRKPWPQKGTGR(A)**	**20.8**	**34**	**1367.7655**	**-0.0655**	**34920/9.7**	**Q9BYD3 Q9BYD3**	HUMAN	39S ribosomal protein L4, mitochondrial
14	**12**	**(A)ASHLEAIRARDEASHLEAIRARDE(W)**	**20.7**	**36**	**1367.7026**	**-0.00260**	**23390/11.2**	**Q2TAM9 Q2TAM9**	HUMAN	Tumor suppressor candidate gene 1 protein
14	**11**	**(L)RHDLAQLQAAASSRHDLAQLQAAASS(A)**	**20.7**	**36**	**1367.7026**	**-0.00260**	**128121/7.4**	**Q14147 Q14147**	HUMAN	Probable ATP-dependent RNA helicase DHX34
15	**11**	**(P)GTGHPPSKRARGFGTGHPPSKRARGF(S)**	**20.6**	**39**	**1367.7291**	**-0.0291**	**85839/5.9**	**Q8WXE1 Q8WXE1**	HUMAN	ATR-interacting protein
16	**10**	**(R)RTRSSGASHQPSPRTRSSGASHQPSP(T)**	**20.5**	**42**	**1367.6774**	**0.0226**	**86372/5.5**	**Q8TBP0 Q8TBP0**	HUMAN	TBC1 domain family member 16
16	**12**	**(T)RGEESSSDTNKSARGEESSSDTNKSA(-)**	**20.5**	**42**	**1367.6033**	**0.0967**	**36428/5.2**	**P78410 P78410**	HUMAN	Butyrophilin subfamily 3 member A2
17	**11**	**(H)TPDHQGEGRGSARTPDHQGEGRGSAR(N)**	**20.4**	**44**	**1367.6411**	**0.0589**	**73307/6.1**	**P0C7T5 P0C7T5**	HUMAN	Ataxin-1-like
18	**13**	**(F)RPPSQRVSTSPVGRPPSQRVSTSPVG(L)**	**20.3**	**48**	**1367.7390**	**-0.0390**	**269460/5.5**	**Q8IWZ3 Q8IWZ3**	HUMAN	Ankyrin repeat and KH domain-containing protein 1
18	**11**	**(T)ASKGRVQIHDTRASKGRVQIHDTR(P)**	**20.3**	**48**	**1367.7502**	**-0.0502**	**130961/6.4**	**Q86XI2 Q86XI2**	HUMAN	Condensin-2 complex subunit G2
19	**10**	**(D)YSRSDASSPTISPYSRSDASSPTISP(A)**	**20.2**	**51**	**1367.6437**	**0.0563**	**31180/9.6**	**Q9Y2Y9 Q9Y2Y9**	HUMAN	Krueppel-like factor 13
20	**13**	**(A)VGSAM(Oxidation)FLRFINPVGSAM(Oxidation)FLRFINP(A)**	**20.1**	**55**	**1367.7140**	**-0.0140**	**319375/7.1**	**P21359 P21359**	HUMAN	Neurofibromin
