# Supplementary material for: MALDI-TOF peptidomic analysis of serum and post-prostatic massage urine specimens to identify prostate cancer biomarkers
Source: Clin Proteomics. 2018 Jul 25;15:23. doi: 10.1186/s12014-018-9199-8 (PMC6060548; doi:10.1186/s12014-018-9199-8)
Supplement: Supplementary file 14 — Additional file 14: MS-Tag search results. MS-MS spectra, peptide lists and MS-Tag search results (including all the configuration parameter) for the fragmentation patters of the 12 MALDI-TOF/MS serum features. [file 12014_2018_9199_MOESM14_ESM.zip › New folder/1419_3.pdf]

MS-Tag Search Results

Search completed. 15 sec elapsed. 0 sec remaining.

[–] **Parameters**

Database searched: **SwissProt.2016.5.30**
Digest Used: **No enzyme**
Max. # Missed Cleavages: **1**
Ion Types Considered: **a, a-NH3, a-H2O, b, b-NH3, b-H2O, b+H2O, y, y-NH3, y-H2O, I, i, P, S, M-H2O, M-NH3, M-SOCH4**
Search Mode:
Max Modifications: **2**
Peptide Masses are: **monoisotopic**

[–] **Pre Search Results (SwissProt.2016.5.30)**

Number of entries in the database: **551193**
Full Molecular Weight range: **551193** entries.
Full pI range: **551193** entries.
Taxonomy search **HOMO SAPIENS** selects **20202** entries.
Pre searches select **20202** entries.

Results

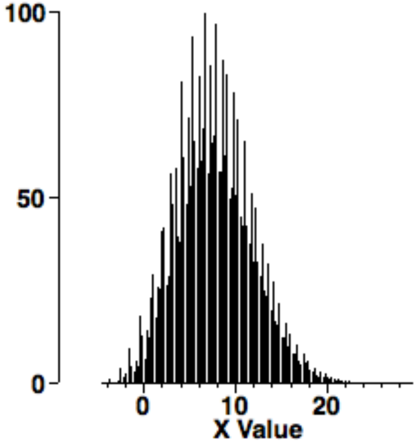

<< < > >> >< <> All

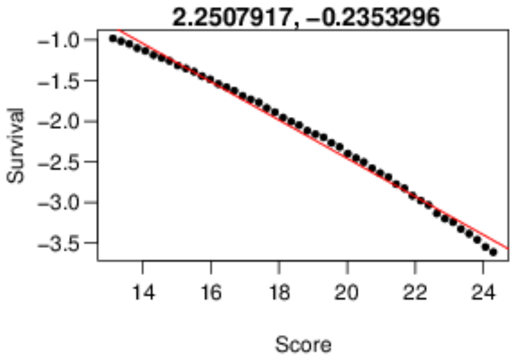

expectation value = 4.98
num peptides considered = 231741
MS-Tag search selects **32** entries (results displayed for top **30** matches).

Parent mass: **1419.3500 (+/- 0.500 Da)**
[–] **Fragment Ions**

48 Ions used in search: **23.0300, 39.0000, 70.1100, 86.1200, 104.1300, 110.1100, 112.1100, 120.1100, 184.0700, 238.1000, 262.1200, 333.2500, 447.2800, 479.2300, 519.2300, 536.3000, 550.3400, 562.3500, 588.2700, 590.3800, 604.3900, 608.3400, 676.4000, 679.3400, 718.4700, 733.5100, 762.3500, 780.3600, 791.4600, 805.5300, 808.4200, 851.4600, 862.5900, 891.4500, 905.4600, 922.5000, 966.5000, 1020.5400, 1037.5800, 1067.7500, 1091.8200, 1134.6300, 1151.6700, 1214.9000, 1260.8700, 1301.9200, 1301.9200, 1347.9100 (+/- 1.00 Da)**

Rank	# Unmatched Ions	Sequence	Score	Expect	MH^+ Calculated (Da)	Error (Da)	Protein MW (Da)/pI	Accession #	Species	Protein Name
1	**20**	**(P)PRPPATSTAASPLGPPRPPATSTAASPLGP(L)**	**29.4**	**5.0**	**1419.7591**	**-0.409**	**26629/9.4**	**E7ERA6 E7ERA6**	HUMAN	RING finger protein 223
2	**23**	**(T)ERPAQ(Deamidated)PGAANPLVVERPAQ(Deamidated)PGAANPLVV(G)**	**29.2**	**5.5**	**1419.7591**	**-0.409**	**65861/6.4**	**Q96JJ6 Q96JJ6**	HUMAN	Junctophilin-4
3	**23**	**(E)ASMLHALQHPCIVASMLHALQHPCIV(A)**	**28.6**	**7.7**	**1419.7235**	**-0.374**	**225395/6.3**	**Q38SD2 Q38SD2**	HUMAN	Leucine-rich repeat serine/threonine-protein kinase 1
4	**25**	**(L)ERHQETCM(Oxidation)RDVERHQETCM(Oxidation)RDV(R)**	**28.5**	**8.1**	**1419.6104**	**-0.260**	**61182/8.8**	**Q49AA0 Q49AA0**	HUMAN	Zinc finger protein ZFP69
5	**26**	**(L)GPRAPASPAPAQAQTGPRAPASPAPAQAQT(P)**	**28.2**	**9.5**	**1419.7339**	**-0.384**	**51075/6.6**	**Q2NL68 Q2NL68**	HUMAN	Proline and serine-rich protein 3
6	**25**	**(E)AGRPADKIQM(Oxidation)AM(Oxidation)VAGRPADKIQM(Oxidation)AM(Oxidation)V(H)**	**28.1**	**10**	**1419.7083**	**-0.358**	**114570/6.8**	**Q07912 Q07912**	HUMAN	Activated CDC42 kinase 1
7	**19**	**(G)GM(Oxidation)ENM(Oxidation)GRFGSGMNGM(Oxidation)ENM(Oxidation)GRFGSGMN(M)**	**27.7**	**13**	**1419.5450**	**-0.195**	**77516/8.8**	**P52272 P52272**	HUMAN	Heterogeneous nuclear ribonucleoprotein M
										Cold shock

8	**20**	**(L)RQ(Deamidated)PRGPDNSMGFGRQ(Deamidated)PRGPDNSMGFG(A)**	**27.4**	**15**	**1419.6434**	**-0.293**	**88885/5.9**	**O75534 O75534**	HUMAN	domain-containing protein E1
9	**23**	**(T)LPRSPASVFMEQ(Deamidated)GLPRSPASVFMEQ(Deamidated)G(S)**	**27.2**	**16**	**1419.6937**	**-0.344**	**372823/4.4**	**P13611 P13611**	HUMAN	Versican core protein
9	**29**	**(A)AAAAAAAAAATGPQGLHAAAAAAAAAATGPQGLH(L)**	**27.2**	**16**	**1419.7339**	**-0.384**	**48389/10.0**	**Q9HAH7 Q9HAH7**	HUMAN	Probable fibrosin-1
10	**23**	**(M)PM(Oxidation)GRFGGSGGMNHVPM(Oxidation)GRFGGSGGMNHV(S)**	**26.8**	**20**	**1419.6256**	**-0.276**	**159158/6.2**	**Q15596 Q15596**	HUMAN	Nuclear receptor coactivator 2
10	**27**	**(G)VTGAHGFPCCGKGSVVTGAHGFPCCGKGSV(E)**	**26.8**	**20**	**1419.6508**	**-0.301**	**42326/4.7**	**Q96LK8 Q96LK8**	HUMAN	Spermatogenesis-associated protein 32
11	**23**	**(L)ADHRELGKMM(Oxidation)N(Deamidated)TADHRELGKMM(Oxidation)N(Deamidated)T(I)**	**26.7**	**22**	**1419.6355**	**-0.286**	**128791/6.2**	**Q9Y2A7 Q9Y2A7**	HUMAN	Nck-associated protein 1
11	**23**	**(Y)MYSAQGNGGGPPRKMYSAQGNGGGPPRK(G)**	**26.7**	**22**	**1419.6798**	**-0.330**	**89632/5.9**	**Q96KG9 Q96KG9**	HUMAN	N-terminal kinase-like protein
12	**26**	**(T)PQRPGAN(Deamidated)PNPGQSVPQRPGAN(Deamidated)PNPGQSV(S)**	**26.6**	**23**	**1419.6975**	**-0.348**	**53537/5.4**	**O15079 O15079**	HUMAN	Syntaphilin
12	**28**	**(D)GLPGPKGAQGERGPVGLPGPKGAQGERGPV(G)**	**26.6**	**23**	**1419.7703**	**-0.420**	**144911/6.1**	**P05997 P05997**	HUMAN	Collagen alpha-2(V) chain
13	**26**	**(P)VDAIVLEAPFTNMVDAIVLEAPFTNM(W)**	**26.5**	**24**	**1419.7188**	**-0.369**	**40777/8.6**	**Q7Z5M8 Q7Z5M8**	HUMAN	Protein ABHD12B
13	**25**	**(S)AGPRPKARHQ(Deamidated)AETAGPRPKARHQ(Deamidated)AET(S)**	**26.5**	**24**	**1419.7451**	**-0.395**	**83858/7.2**	**Q12815 Q12815**	HUMAN	Tastin
14	**24**	**(S)RPPVTTSNAIPPAVRPPVTTSNAIPPAV(V)**	**26.4**	**25**	**1419.7954**	**-0.445**	**110325/9.8**	**Q9H0E3 Q9H0E3**	HUMAN	Histone deacetylase complex subunit SAP130
15	**27**	**(P)ASTPPQ(Deamidated)GSMANSTAVASTPPQ(Deamidated)GSMANSTAV(V)**	**26.1**	**30**	**1419.6420**	**-0.292**	**17925/7.8**	**Q16514 Q16514**	HUMAN	Transcription initiation factor TFIID subunit 12
15	**24**	**(G)TMTYLSKAAATYVTMTYLSKAAATYV(Q)**	**26.1**	**30**	**1419.7188**	**-0.369**	**137313/5.8**	**P46020 P46020**	HUMAN	Phosphorylase b kinase regulatory subunit alpha, skeletal muscle isoform
16	**26**	**(T)PQ(Deamidated)RPGANPNPGQSVPQ(Deamidated)RPGANPNPGQSV(S)**	**25.9**	**33**	**1419.6975**	**-0.348**	**53537/5.4**	**O15079 O15079**	HUMAN	Syntaphilin
16	**23**	**(N)KRPDPFPM(Oxidation)PTFAKRPDPFPM(Oxidation)PTFA(A)**	**25.9**	**33**	**1419.7089**	**-0.359**	**35170/8.5**	**Q8N5C1 Q8N5C1**	HUMAN	Protein FAM26E
17	**24**	**(G)AKPPRGASTGGSGGYGAKPPRGASTGGSGGYG(G)**	**25.7**	**37**	**1419.6975**	**-0.348**	**44995/8.7**	**Q8TDD2 Q8TDD2**	HUMAN	Transcription factor Sp7
18	**26**	**(T)PQRPGANPNPGQ(Deamidated)SVPQRPGANPNPGQ(Deamidated)SV(S)**	**25.6**	**39**	**1419.6975**	**-0.348**	**53537/5.4**	**O15079 O15079**	HUMAN	Syntaphilin
19	**27**	**(I)DFHNPDRAYDAVDFHNPDRAYDAV(Q)**	**25.5**	**41**	**1419.6288**	**-0.279**	**40924/7.5**	**P19086 P19086**	HUMAN	Guanine nucleotide-binding protein G(z) subunit

										alpha
19	**26**	**(T)SRRPCHSQ(Deamidated)DFSVSRRPCHSQ(Deamidated)DFSV(H)**	**25.5**	**41**	**1419.6434**	**-0.293**	**81668/6.2**	**Q96SM3 Q96SM3**	HUMAN	Probable carboxypeptidase X1
19	**28**	**(G)CASAAPRRGPALLHCASAAPRRGPALLH(I)**	**25.5**	**41**	**1419.7638**	**-0.414**	**168418/8.3**	**Q03468 Q03468**	HUMAN	DNA excision repair protein ERCC-6
20	**24**	**(A)AEPRPGAGSLQ(Deamidated)HAQAEPRPGAGSLQ(Deamidated)HAQ(P)**	**25.4**	**43**	**1419.6975**	**-0.348**	**63152/7.0**	**O15530 O15530**	HUMAN	3-phosphoinositide-dependent protein kinase 1
20	**24**	**(A)AEPRPGAGSLQ(Deamidated)HAQAEPRPGAGSLQ(Deamidated)HAQ(P)**	**25.4**	**43**	**1419.6975**	**-0.348**	**44766/8.6**	**Q6A1A2 Q6A1A2**	HUMAN	Putative 3-phosphoinositide-dependent protein kinase 2
20	**26**	**(E)KRQHSSQDVHVVKRQHSSQDVHVV(L)**	**25.4**	**43**	**1419.7451**	**-0.395**	**40573/5.0**	**Q9UNZ2 Q9UNZ2**	HUMAN	NSFL1 cofactor p47
