# Supplementary material for: MALDI-TOF peptidomic analysis of serum and post-prostatic massage urine specimens to identify prostate cancer biomarkers
Source: Clin Proteomics. 2018 Jul 25;15:23. doi: 10.1186/s12014-018-9199-8 (PMC6060548; doi:10.1186/s12014-018-9199-8)
Supplement: Supplementary file 14 — Additional file 14: MS-Tag search results. MS-MS spectra, peptide lists and MS-Tag search results (including all the configuration parameter) for the fragmentation patters of the 12 MALDI-TOF/MS serum features. [file 12014_2018_9199_MOESM14_ESM.zip › New folder/1504_7.pdf]

MS-Tag Search Results

Search completed. 2 sec elapsed. 0 sec remaining.

[+] **Parameters**

[+] **Pre Search Results (SwissProt.2017.11.01)**

Results

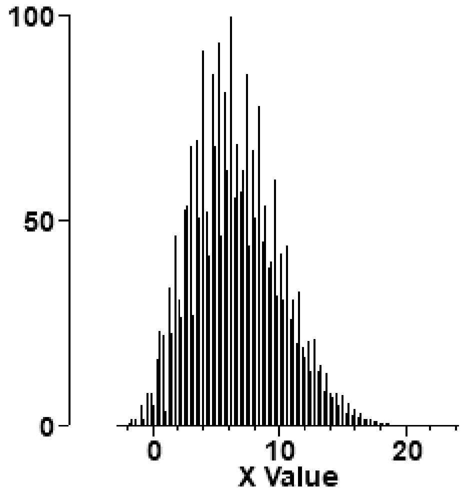

<< < > >> >< <> All

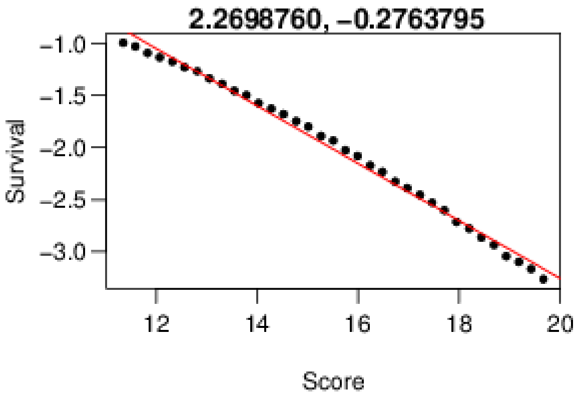

expectation value = 4.71
num peptides considered = 123476
MS-Tag search selects **35** entries (results displayed for top **30** matches).

Parent mass: **1504.7000 (+/- 0.500 Da)**
[+] **Fragment Ions**

Rank	# Unmatched Ions	Sequence	Score	Expect	MH^+ Calculated (Da)	Error (Da)	Protein MW (Da)/pI	Accession #	Species	Protein Name
1	**10**	**(G)GGGGSGASSGGGAGGLQPSS(R)**	**24.2**	**4.7**	**1504.6623**	**0.0377**	**152788/5.5**	**Q9ULU8**	HUMAN	Calcium-dependent secretion activator 1
2	**11**	**(R)SSKNETKGRGSPKE(K)**	**22.8**	**11**	**1504.7714**	**-0.0714**	**45287/8.4**	**Q9NYS7**	HUMAN	WD repeat and SOCS box-containing protein 2
3	**13**	**(L)HQLLSLHGGSTPGSGG(P)**	**22.4**	**15**	**1504.7503**	**-0.0503**	**74699/9.9**	**P51787**	HUMAN	Potassium voltage-gated channel subfamily KQT member 1
4	**13**	**(R)PSPPPLGFRDEHGV(A)**	**22.3**	**16**	**1504.7543**	**-0.0543**	**36335/8.5**	**P52961**	HUMAN	GPI-linked NAD(P)(+)--arginine ADP-ribosyltransferase 1
5	**10**	**(G)KVSGHLHVGHTPVH(F)**	**22.2**	**17**	**1504.8132**	**-0.113**	**151255/5.1**	**Q14112**	HUMAN	Nidogen-2
6	**13**	**(P)GMQNERHGQSFVD(S)**	**21.9**	**20**	**1504.6598**	**0.0402**	**335930/6.0**	**Q9P2D1**	HUMAN	Chromodomain-helicase-DNA-binding protein 7
7	**14**	**(H)TYGGGGGPDALLGTLR(V)**	**21.8**	**22**	**1504.7754**	**-0.0754**	**39744/11.3**	**Q8NCU7**	HUMAN	C2 calcium-dependent domain-containing protein 4A
8	**12**	**(W)AGHGGAGGTDRGAQHR(A)**	**21.7**	**23**	**1504.7112**	**-0.0112**	**33953/5.8**	**A8MZ36**	HUMAN	Envoplakin-like protein
9	**12**	**(D)AATIPSSAMQAPTVC(Carbamidomethyl)(I)**	**21.5**	**26**	**1504.7134**	**-0.0134**	**73355/9.4**	**Q96GD3**	HUMAN	Polycomb protein SCMH1

10	**12**	**(T)LNSFVIPSESDVPT(H)**	**21.4**	**28**	**1504.7530**	**-0.0530**	**166013/6.1**	**P22897**	HUMAN	Macrophage mannose receptor 1
11	**12**	**(E)RGPPAPRHQEMASA(S)**	**21.1**	**34**	**1504.7438**	**-0.0438**	**53675/6.8**	**O60304**	HUMAN	Zinc finger protein 500
12	**12**	**(H)SAGTNGTGVSM(Oxidation)EPSPP(T)**	**20.9**	**38**	**1504.6584**	**0.0416**	**59545/9.0**	**Q86XZ4**	HUMAN	Spermatogenesis-associated serine-rich protein 2
13	**12**	**(L)ASHEDSVPSAM(Oxidation)TTR(L)**	**20.8**	**41**	**1504.6696**	**0.0304**	**106162/6.1**	**Q8NDX5**	HUMAN	Polyhomeotic-like protein 3
13	**15**	**(G)FDPVSTVLHELTF(Q)**	**20.8**	**41**	**1504.7682**	**-0.0682**	**67765/8.0**	**O00186**	HUMAN	Syntaxin-binding protein 3
13	**11**	**(L)SLRHNHITELER(D)**	**20.8**	**41**	**1504.7979**	**-0.0979**	**59077/8.3**	**O43300**	HUMAN	Leucine-rich repeat transmembrane neuronal protein 2
14	**15**	**(S)ASSSASLRVLGPGMDG(I)**	**20.7**	**44**	**1504.7424**	**-0.0424**	**346903/5.9**	**O75962**	HUMAN	Triple functional domain protein
14	**12**	**(T)HRIHWESASLLR(S)**	**20.7**	**44**	**1504.8132**	**-0.113**	**187150/6.0**	**P01024**	HUMAN	Complement C3
15	**14**	**(K)C(Carbamidomethyl)AFSHQGSIQVDR(N)**	**20.6**	**47**	**1504.6961**	**0.00386**	**59348/9.0**	**Q02928**	HUMAN	Cytochrome P450 4A11
15	**13**	**(N)NHSFSTANGLSVDR(L)**	**20.6**	**47**	**1504.7139**	**-0.0139**	**52353/9.0**	**P55316**	HUMAN	Forkhead box protein G1
15	**10**	**(A)SHRRHRGGDLGTR(R)**	**20.6**	**47**	**1504.7952**	**-0.0952**	**143753/8.7**	**O15399**	HUMAN	Glutamate receptor ionotropic, NMDA 2D
16	**12**	**(L)QPSPMSSNPSITGSD(V)**	**20.5**	**50**	**1504.6584**	**0.0416**	**133282/8.0**	**Q16825**	HUMAN	Tyrosine-protein phosphatase non-receptor type 21
17	**12**	**(D)ASGTTPSSAHSTTSGR(G)**	**20.4**	**53**	**1504.6986**	**0.00136**	**558168/5.3**	**Q9UKN1**	HUMAN	Mucin-12
17	**12**	**(D)ASGTTPSSAHSTTSGR(G)**	**20.4**	**53**	**1504.6986**	**0.00136**	**558168/5.3**	**Q9UKN1**	HUMAN	Mucin-12
17	**12**	**(D)ASGTTPSSAHSTTSGR(G)**	**20.4**	**53**	**1504.6986**	**0.00136**	**558168/5.3**	**Q9UKN1**	HUMAN	Mucin-12
17	**12**	**(D)ASGTTPSSAHSTTSGR(G)**	**20.4**	**53**	**1504.6986**	**0.00136**	**558168/5.3**	**Q9UKN1**	HUMAN	Mucin-12
17	**10**	**(D)GASSDGHLAHIFRH(A)**	**20.4**	**53**	**1504.7404**	**-0.0404**	**51157/6.6**	**Q9UHQ1**	HUMAN	Nuclear prelamin A recognition factor
17	**13**	**(E)RSPM(Oxidation)SARLASPC(Carbamidomethyl)R(A)**	**20.4**	**53**	**1504.7471**	**-0.0471**	**89816/9.4**	**Q8N393**	HUMAN	Zinc finger protein 786
17	**12**	**(Q)LPAPQPLASSAGHSTA(S)**	**20.4**	**53**	**1504.7754**	**-0.0754**	**81084/6.0**	**Q9HC78**	HUMAN	Zinc finger and BTB domain-containing protein 20
18	**12**	**(A)GHGGAGGTDRGAQHRA(E)**	**20.3**	**56**	**1504.7112**	**-0.0112**	**33953/5.8**	**A8MZ36**	HUMAN	Envoplakin-like protein
18	**12**	**(L)IGHEGDEVFAARFG(P)**	**20.3**	**56**	**1504.7179**	**-0.0179**	**36495/9.4**	**P28356**	HUMAN	Homeobox protein Hox-D9
18	**10**	**(V)LSTTGYGHTVPLSDG(G)**	**20.3**	**56**	**1504.7278**	**-0.0278**	**38143/5.9**	**O00180**	HUMAN	Potassium channel subfamily K member 1
18	**15**	**(L)LSDGKASISMPREGG(S)**	**20.3**	**56**	**1504.7424**	**-0.0424**	**35691/6.0**	**Q96F83**	HUMAN	Uncharacterized protein C14orf79

19	**13**	**(V)SSPNLASDSDLSRR(S)**	**20.2**	**60**	**1504.7350**	**-0.0350**	**56466/5.1**	**Q09470**	HUMAN	Potassium voltage-gated channel subfamily A member 1
