# Supplementary material for: MALDI-TOF peptidomic analysis of serum and post-prostatic massage urine specimens to identify prostate cancer biomarkers
Source: Clin Proteomics. 2018 Jul 25;15:23. doi: 10.1186/s12014-018-9199-8 (PMC6060548; doi:10.1186/s12014-018-9199-8)
Supplement: Supplementary file 14 — Additional file 14: MS-Tag search results. MS-MS spectra, peptide lists and MS-Tag search results (including all the configuration parameter) for the fragmentation patters of the 12 MALDI-TOF/MS serum features. [file 12014_2018_9199_MOESM14_ESM.zip › New folder/1605_8.pdf]

MS-Tag Search Results

Search completed. 13 sec elapsed. 0 sec remaining.

[–] **Parameters**

Database searched: **SwissProt.2016.5.30**
Digest Used: **No enzyme**
Max. # Missed Cleavages: **1**
Constant Modification: **Carbamidomethyl (C)**
Ion Types Considered: **a, a-NH3, a-H2O, b, b-NH3, b-H2O, b+H2O, y, y-NH3, y-H2O, I, i, P, S, M-H2O, M-NH3, M-SOCH4**
Search Mode:
Max Modifications: **2**
Peptide Masses are: **monoisotopic**

[–] **Pre Search Results (SwissProt.2016.5.30)**

Number of entries in the database: **551193**
Full Molecular Weight range: **551193** entries.
Full pI range: **551193** entries.
Taxonomy search **HOMO SAPIENS** selects **20202** entries.
Pre searches select **20202** entries.

Results

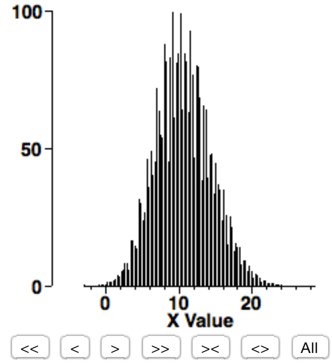

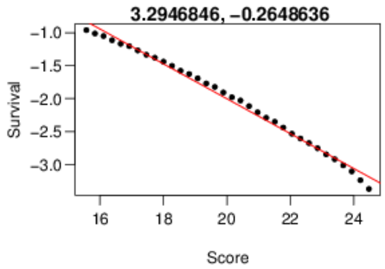

expectation value = 7.44
num peptides considered = 125114
MS-Tag search selects **32** entries (results displayed for top **30** matches).

Parent mass: **1605.8000 (+/- 0.500 Da)**
[–] **Fragment Ions**

43 Ions used in search: **23.0000, 39.0000, 60.1000, 70.1000, 83.6000, 86.1000, 87.1000, 110.1000, 112.1000, 115.1000, 130.1000, 138.1000, 159.1000, 166.1000, 175.1000, 184.0000, 195.1000, 207.1000, 211.1000, 216.1000, 239.1000, 251.2000, 277.1000, 296.1000, 324.1000, 334.2000, 378.2000, 390.3000, 395.2000, 466.3000, 491.3000, 803.5000, 961.6000, 1098.7000, 1136.7000, 1349.0000, 1432.1000, 1450.1000, 1545.2000, 1546.3000, 1562.2000, 1564.3000, 1589.8000 (+/- 1.00 Da)**

Rank	# Unmatched Ions	Sequence	Score	Expect	MH^+ Calculated (Da)	Error (Da)	
1	**16**	**(I)THRIHWESASLLRTHRIHWESASLLR(S)**	**28.4**	**7.4**	**1605.8608**	**-0.0608**	**1**
1	**15**	**(S)EEHLSPQSFAM(Oxidation)GTREEHLSPQSFAM(Oxidation)GTR(M)**	**28.4**	**7.4**	**1605.7326**	**0.0674**	**1**
2	**17**	**(C)DAPPLVKM(Oxidation)SC(Carbamidomethyl)TNTRDAPPLVKM(Oxidation)SC(Carbamidomethyl)TNTR(V)**	**27.9**	**10**	**1605.7723**	**0.0277**	[illegible]
3	**20**	**(L)M(Oxidation)EGGTHM(Oxidation)VC(Carbamidomethyl)TGRTHM(Oxidation)EGGTHM(Oxidation)VC(Carbamidomethyl)TGRTH(T)**	**27.5**	**13**	**1605.6567**	**0.143**	[illegible]
4	**17**	**(G)HLTGKHERHFSISGHLTGKHERHFSISG(C)**	**27.3**	**15**	**1605.8244**	**-0.0244**	[illegible]
5	**18**	**(R)PPARSTASGHDRPTRPPARSTASGHDRPTR(G)**	**27.2**	**15**	**1605.8204**	**-0.0204**	[illegible]
6	**16**	**(A)SSSHAQGTHLRLRRSSSHAQGTHLRLRR(C)**	**26.9**	**19**	**1605.8680**	**-0.0680**	**1**
6	**18**	**(L)GVPHASEQRYDAEFGVPHASEQRYDAEF(F)**	**26.9**	**19**	**1605.7292**	**0.0708**	**2**
7	**19**	**(T)RHTGEAYDPSNTEERHTGEAYDPSNTEE(I)**	**26.7**	**21**	**1605.6776**	**0.122**	**2**
8	**20**	**(S)SGSHTSSASVTSVRSRSGSHTSSASVTSVRSR(T)**	**26.6**	**22**	**1605.7939**	**0.00606**	[illegible]
9	**20**	**(S)KGAHTM(Oxidation)C(Carbamidomethyl)ASSLSSPRKGAHTM(Oxidation)C(Carbamidomethyl)ASSLSSPR(A)**	**26.3**	**27**	**1605.7472**	**0.0528**	[illegible]

10	**18**	**(K)AKHSSNKPHKAASSRAKHSSNKPHKAASSR(I)**	**26.1**	**30**	**1605.8568**	**-0.0568**	**1**
10	**20**	**(M)KEKRVLLSADGPHRKEKRVLLSADGPHR(N)**	**26.1**	**30**	**1605.9183**	**-0.118**	[illegible]
11	**19**	**(L)GVFSALRAVPQKEGFGVFSALRAVPQKEGF(L)**	**26.0**	**32**	**1605.8748**	**-0.0748**	[illegible]
12	**19**	**(D)RTHPSAAVPVC(Carbamidomethyl)PRSARTHPSAAVPVC(Carbamidomethyl)PRSA(S)**	**25.9**	**34**	**1605.8278**	**-0.0278**	**9**
13	**19**	**(P)EQHLDVSSTTPSPAHEQHLDVSSTTPSPAH(K)**	**25.7**	**39**	**1605.7503**	**0.0497**	[illegible]
14	**17**	**(H)AAHRANSASRAPPSGRAAHRANSASRAPPSGR(A)**	**25.5**	**44**	**1605.8317**	**-0.0317**	[illegible]
15	**17**	**(G)IPSAPSSFYSHREKIPSAPSSFYSHREK(P)**	**25.4**	**46**	**1605.8020**	**-0.00198**	**4**
15	**16**	**(I)INIFSVASGHLYERINIFSVASGHLYER(F)**	**25.4**	**46**	**1605.8384**	**-0.0384**	**1**
15	**16**	**(V)LNIFSVASGHLYERLNIFSVASGHLYER(F)**	**25.4**	**46**	**1605.8384**	**-0.0384**	**1**
15	**20**	**(E)EDPAHAFEAFQSTREDPAHAFEAFQSTR(S)**	**25.4**	**46**	**1605.7292**	**0.0708**	**1**
16	**20**	**(P)LAVPPNPGGGSRRATRLAVPPNPGGGSRRATR(G)**	**25.3**	**49**	**1605.8932**	**-0.0932**	**1**
17	**20**	**(K)GHQGLQARLGPEQQSGHQGLQARLGPEQQS(-)**	**25.2**	**52**	**1605.8092**	**-0.00919**	**2**
17	**18**	**(P)RMAAAHGPVAPSSPEQRMAAAHGPVAPSSPEQ(V)**	**25.2**	**52**	**1605.7802**	**0.0198**	[illegible]
18	**19**	**(Q)RHTPQSPFTNHAAAARHTPQSPFTNHAAAA(G)**	**25.1**	**56**	**1605.7881**	**0.0119**	[illegible]
18	**19**	**(S)PADHSALPNQSLTVRPADHSALPNQSLTVR(E)**	**25.1**	**56**	**1605.8343**	**-0.0343**	**3**
19	**20**	**(Q)TGHVPPQGGTHRPPAPTGHVPPQGGTHRPPAP(A)**	**25.0**	**59**	**1605.8244**	**-0.0244**	[illegible]
19	**18**	**(N)SLGHLTGKHERHFSSLGHLTGKHERHFS(I)**	**25.0**	**59**	**1605.8244**	**-0.0244**	[illegible]
19	**19**	**(G)LPGPQGPSGAKGEPGTRLPGPQGPSGAKGEPGTR(G)**	**25.0**	**59**	**1605.8343**	**-0.0343**	[illegible]
19	**20**	**(W)KHGFFSLTAVGATNVGKHGFFSLTAVGATNVG(S)**	**25.0**	**59**	**1605.8384**	**-0.0384**	[illegible]
19	**19**	**(E)DTAAHIASLKASHQRDTAAHIASLKASHQR(E)**	**25.0**	**59**	**1605.8456**	**-0.0456**	**1**
