# Supplementary material for: MALDI-TOF peptidomic analysis of serum and post-prostatic massage urine specimens to identify prostate cancer biomarkers
Source: Clin Proteomics. 2018 Jul 25;15:23. doi: 10.1186/s12014-018-9199-8 (PMC6060548; doi:10.1186/s12014-018-9199-8)
Supplement: Supplementary file 14 — Additional file 14: MS-Tag search results. MS-MS spectra, peptide lists and MS-Tag search results (including all the configuration parameter) for the fragmentation patters of the 12 MALDI-TOF/MS serum features. [file 12014_2018_9199_MOESM14_ESM.zip › New folder/1739_9.pdf]

MS-Tag Search Results

Search completed. 13 sec elapsed. 0 sec remaining.

[–] **Parameters**

Database searched: **SwissProt.2016.5.30**
Digest Used: **No enzyme**
Max. # Missed Cleavages: **1**
Constant Modification: **Carbamidomethyl (C)**
Ion Types Considered: **a, a-NH3, a-H2O, b, b-NH3, b-H2O, b+H2O, y, y-NH3, y-H2O, I, i, P, S, M-H2O, M-NH3, M-SOCH4**
Search Mode:
Max Modifications: **2**
Peptide Masses are: **monoisotopic**

[–] **Pre Search Results (SwissProt.2016.5.30)**

Number of entries in the database: **551193**
Full Molecular Weight range: **551193** entries.
Full pI range: **551193** entries.
Taxonomy search **HOMO SAPIENS** selects **20202** entries.
Pre searches select **20202** entries.

Results

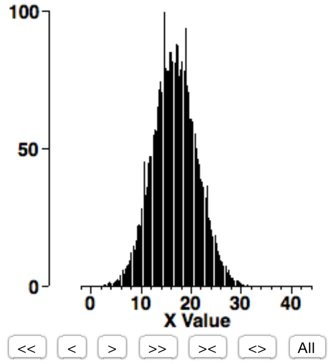

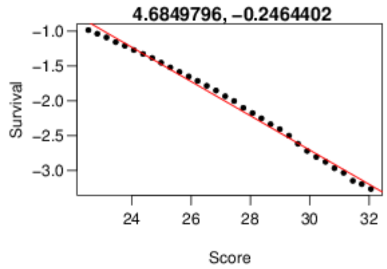

expectation value = 0.0781
num peptides considered = 125868
MS-Tag search selects **39** entries (results displayed for top **30** matches).

Parent mass: **1739.9000 (+/- 0.500 Da)**
[–] **Fragment Ions**

42 Ions used in search: **69.7000, 83.1000, 84.1000, 86.1000, 87.1000, 101.1000, 104.1000, 109.3000, 110.1000, 112.1000, 120.1000, 129.1000, 154.1000, 172.1000, 181.1000, 184.1000, 185.1000, 197.1000, 198.1000, 209.1000, 225.1000, 228.1000, 234.1000, 242.2000, 296.1000, 319.2000, 319.2000, 322.1000, 325.2000, 479.2000, 513.3000, 530.3000, 534.3000, 643.4000, 855.5000, 1069.8000, 1206.9000, 1581.3000, 1582.4000, 1609.5000, 1610.4000, 1626.4000 (+/- 1.00 Da)**

Rank	# Unmatched Ions	Sequence	Score	Expect	MH$^+$ Calculated (Da)	Error (Da)	Protein MW (Da)/pI	Accession #	Species	Prote
1	**7**	**(R)NGFKSHALQLNNRQINGFKSHALQLNNRQI(R)**	**44.2**	**0.078**	**1739.9300**	**-0.0300**	**192787/6.7**	**P0C0L4 P0C0L4**	HUMAN	Comp
1	**7**	**(R)NGFKSHALQLNNRQINGFKSHALQLNNRQI(R)**	**44.2**	**0.078**	**1739.9300**	**-0.0300**	**192753/6.9**	**P0C0L5 P0C0L5**	HUMAN	Comp
2	**13**	**(E)SAERSEPSQHVVLSLTSAERSEPSQHVVLSLT(F)**	**36.5**	**6.2**	**1739.8923**	**0.00775**	**65305/8.6**	**P57772 P57772**	HUMAN	Selen elonga
3	**12**	**(N)LRRHYQTNHSKHYLRRHYQTNHSKHY(D)**	**35.4**	**12**	**1739.8837**	**0.0163**	**107232/5.6**	**Q86UP8 Q86UP8**	HUMAN	Gener repeat protei
3	**12**	**(N)LRRHYQTNHSKHYLRRHYQTNHSKHY(D)**	**35.4**	**12**	**1739.8837**	**0.0163**	**107234/5.6**	**Q6EKJ0 Q6EKJ0**	HUMAN	Gener repeat protei
4	**10**	**(K)IATFFSSGQSADAHSITIATFFSSGQSADAHSIT(T)**	**35.3**	**12**	**1739.8235**	**0.0765**	**247175/6.4**	**A6NMZ7 A6NMZ7**	HUMAN	Collag
5	**12**	**(N)ILPSNSYPQREHSVIILPSNSYPQREHSVI(S)**	**34.8**	**16**	**1739.9075**	**-0.00751**	**460969/5.9**	**Q8TCU4 Q8TCU4**	HUMAN	Alstro
5	**13**	**(P)SPATRM(Oxidation)HSGQHSTQAQSPATRM(Oxidation)HSGQHSTQAQ(S)**	**34.8**	**16**	**1739.7878**	**0.112**	**115595/5.9**	**Q12968 Q12968**	HUMAN	Nucle cells,
6	**12**	**(R)SAAHM(Oxidation)RHLLMSQQTLSAAHM(Oxidation)RHLLMSQQTL(R)**	**34.7**	**17**	**1739.8680**	**0.0320**	**38360/8.0**	**Q8N0V3 Q8N0V3**	HUMAN	Putati factor
										Uncha

7	**13**	**(A)GGASPKLRWDHVDSGGTGGASPKLRWDHVDSGGT(R)**	**34.5**	**19**	**1739.8460**	**0.0540**	**31710/6.0**	**O95873 O95873**	HUMAN	C6orf
8	**12**	**(Q)ELQYHRSHLAMELLELQYHRSHLAMELL(W)**	**34.2**	**23**	**1739.8898**	**0.0102**	**53033/8.2**	**Q4KMZ1 Q4KMZ1**	HUMAN	IQ do C
9	**10**	**(P)LLHTVKAGSPSDM(Oxidation)PRDLLHTVKAGSPSDM(Oxidation)PRD(S)**	**34.1**	**24**	**1739.8745**	**0.0255**	**82411/6.8**	**Q8NFM7 Q8NFM7**	HUMAN	Interl
9	**10**	**(G)TGVNSNSEKHADHRSTTGVNSNSEKHADHRST(L)**	**34.1**	**24**	**1739.8056**	**0.0944**	**330469/5.0**	**Q8NFC6 Q8NFC6**	HUMAN	Biorie in cell
10	**11**	**(V)PGFWANVIANHPQMSAPGFWANVIANHPQMSA(L)**	**33.9**	**27**	**1739.8322**	**0.0678**	**35013/5.3**	**Q01534 Q01534**	HUMAN	Testis protei
10	**11**	**(V)PGFWANVIANHPQMSAPGFWANVIANHPQMSA(L)**	**33.9**	**27**	**1739.8322**	**0.0678**	**35100/5.3**	**A6NKD2 A6NKD2**	HUMAN	Testis protei
10	**11**	**(V)PGFWANVIANHPQMSAPGFWANVIANHPQMSA(L)**	**33.9**	**27**	**1739.8322**	**0.0678**	**35101/5.2**	**P0CV98 P0CV98**	HUMAN	Testis protei
10	**11**	**(V)PGFWANVIANHPQMSAPGFWANVIANHPQMSA(L)**	**33.9**	**27**	**1739.8322**	**0.0678**	**35728/5.1**	**P0CV99 P0CV99**	HUMAN	Testis protei
10	**11**	**(V)PGFWANVIANHPQMSAPGFWANVIANHPQMSA(L)**	**33.9**	**27**	**1739.8322**	**0.0678**	**35147/5.2**	**P0CW00 P0CW00**	HUMAN	Testis protei
10	**11**	**(V)PGFWANVIANHPQMSAPGFWANVIANHPQMSA(L)**	**33.9**	**27**	**1739.8322**	**0.0678**	**35728/5.1**	**P0CW01 P0CW01**	HUMAN	Testis protei
10	**14**	**(H)LNRHFISNKPSKVLSLNRHFISNKPSKVLS(P)**	**33.9**	**27**	**1739.9915**	**-0.0915**	**40128/8.7**	**Q8N5D6 Q8N5D6**	HUMAN	Globo acetyl 1
11	**15**	**(Y)NTTC(Carbamidomethyl)HLKNHTGRSTINTTC(Carbamidomethyl)HLKNHTGRSTI(M)**	**33.5**	**34**	**1739.8606**	**0.0394**	**34323/8.0**	**Q8N614 Q8N614**	HUMAN	Trans
11	**13**	**(A)GTYHGDSHLQLERINGTYHGDSHLQLERIN(V)**	**33.5**	**34**	**1739.8460**	**0.0540**	**49573/4.7**	**A6NNZ2 A6NNZ2**	HUMAN	Tubul protei
11	**13**	**(A)GTYHGDSHLQLERINGTYHGDSHLQLERIN(V)**	**33.5**	**34**	**1739.8460**	**0.0540**	**49776/4.8**	**Q3ZCM7 Q3ZCM7**	HUMAN	Tubul
12	**13**	**(D)SADLVAQLRAAHSEGNTSADLVAQLRAAHSEGNT(T)**	**33.4**	**36**	**1739.8671**	**0.0329**	**57489/6.0**	**P78371 P78371**	HUMAN	T-com beta
12	**10**	**(N)LTFATPLQPQSVQRPGLTFATPLQPQSVQRPG(L)**	**33.4**	**36**	**1739.9439**	**-0.0439**	**60423/6.1**	**Q96BT3 Q96BT3**	HUMAN	Centr
12	**11**	**(E)LVGAHTIPLVPLRKLLLVGAHTIPLVPLRKLL(L)**	**33.4**	**36**	**1740.1258**	**-0.226**	**45776/6.1**	**Q8N2R8 Q8N2R8**	HUMAN	Protei
13	**11**	**(V)GTAEPGGSMKTTFTIEIGTAEPGGSMKTTFTIEI(K)**	**33.3**	**38**	**1739.8520**	**0.0480**	**99059/9.2**	**P53814 P53814**	HUMAN	Smoo
13	**14**	**(N)IEIPITKGFPVLLHYIEIPITKGFPVLLHY(Q)**	**33.3**	**38**	**1740.0095**	**-0.109**	**75474/6.2**	**Q9Y450 Q9Y450**	HUMAN	HBS1
14	**11**	**(L)LKHISPDQVPVEYGGTLKHISPDQVPVEYGGT(M)**	**33.2**	**40**	**1739.8963**	**0.00372**	**46146/8.0**	**O76054 O76054**	HUMAN	SEC1
15	**11**	**(D)IRNFQPQVSLHNASEIRNFQPQVSLHNASE(Y)**	**33.1**	**42**	**1739.8824**	**0.0176**	**94385/8.4**	**Q06730 Q06730**	HUMAN	Zinc f
16	**14**	**(L)PSLHVFGDTDKVIPSQPSLHVFGDTDKVIPSQ(E)**	**33.0**	**45**	**1739.8963**	**0.00372**	**24418/6.4**	**Q8WZ82 Q8WZ82**	HUMAN	Estera
16	**12**	**(D)LKTTDLSIQPHSADLTLKTTDLSIQPHSADLT(V)**	**33.0**	**45**	**1739.9174**	**-0.0174**	**616635/5.2**	**Q8IVF2 Q8IVF2**	HUMAN	Protei
17	**15**	**(K)GGGTHPLLVPYDTLTAKGGGTHPLLVPYDTLTAK(E)**	**32.9**	**48**	**1739.9327**	**-0.0327**	**565181/5.2**	**P21817 P21817**	HUMAN	Ryanc
17	**11**	**(S)ASQLEAHNSGTKHKTMASQLEAHNSGTKHKTM(L)**	**32.9**	**48**	**1739.8493**	**0.0507**	**42296/10.0**	**Q9H6B1 Q9H6B1**	HUMAN	Zinc f
17	**13**	**(N)IKNRLQDSAM(Oxidation)KLVHAIKNRLQDSAM(Oxidation)KLVHA(E)**	**32.9**	**48**	**1739.9585**	**-0.0585**	**90956/8.1**	**Q93034 Q93034**	HUMAN	Cullir
17	**11**	**(P)GTPDSIEGVSQHLSPESGTPDSIEGVSQHLSPES(S)**	**32.9**	**48**	**1739.8082**	**0.0918**	**555487/6.1**	**Q2LD37 Q2LD37**	HUMAN	Uncha KIAA
17	**11**	**(A)LGGPKAHRKLQTHPSLLGGPKAHRKLQTHPSL(A)**	**32.9**	**48**	**1740.0027**	**-0.103**	**25029/4.3**	**Q99750 Q99750**	HUMAN	MyoD
17	**13**	**(G)KIVM(Oxidation)TAATKHLTPVTLKIVM(Oxidation)TAATKHLTPVTL(E)**	**32.9**	**48**	**1740.0088**	**-0.109**	**42635/5.7**	**P48448 P48448**	HUMAN	Aldeh family
