# Supplementary material for: MALDI-TOF peptidomic analysis of serum and post-prostatic massage urine specimens to identify prostate cancer biomarkers
Source: Clin Proteomics. 2018 Jul 25;15:23. doi: 10.1186/s12014-018-9199-8 (PMC6060548; doi:10.1186/s12014-018-9199-8)
Supplement: Supplementary file 14 — Additional file 14: MS-Tag search results. MS-MS spectra, peptide lists and MS-Tag search results (including all the configuration parameter) for the fragmentation patters of the 12 MALDI-TOF/MS serum features. [file 12014_2018_9199_MOESM14_ESM.zip › New folder/1847_0.pdf]

MS-Tag Search Results

Search completed. 13 sec elapsed. 0 sec remaining.

[–] **Parameters**

Database searched: **SwissProt.2016.5.30**
Digest Used: **No enzyme**
Max. # Missed Cleavages: **1**
Constant Modification: **Carbamidomethyl (C)**
Ion Types Considered: **a, a-NH3, a-H2O, b, b-NH3, b-H2O, b+H2O, y, y-NH3, y-H2O, I, i, P, S, M-H2O, M-NH3, M-SOCH4**
Search Mode:
Max Modifications: **2**
Peptide Masses are: **monoisotopic**

[–] **Pre Search Results (SwissProt.2016.5.30)**

Number of entries in the database: **551193**
Full Molecular Weight range: **551193** entries.
Full pI range: **551193** entries.
Taxonomy search **HOMO SAPIENS** selects **20202** entries.
Pre searches select **20202** entries.

Results

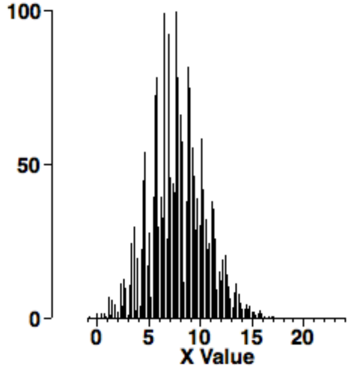

<< < > >> >< <> All

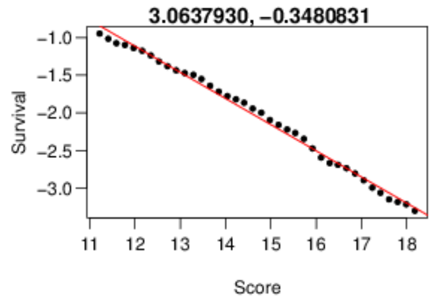

expectation value = 0.610
num peptides considered = 129039
MS-Tag search selects **33** entries (results displayed for top **30** matches).

Parent mass: **1847.0000 (+/- 0.500 Da)**
[–] **Fragment Ions**

22 Ions used in search: **70.1000, 84.1000, 86.1000, 104.1000, 110.1000, 112.1000, 128.1000, 129.1000, 155.1000, 159.1000, 211.1000, 222.1000, 224.4000, 225.2000, 239.1000, 277.1000, 378.2000, 584.3000, 1030.6000, 1201.8000, 1491.7000, 1691.4000 (+/- 1.00 Da)**

Rank	# Unmatched Ions	Sequence	Score	Expect	MH^+ Calculated (Da)	Error (Da)	Protein MW (Da)/pI	Accession #	Species	Protein Name
1	**4**	**(A)TFRLKDGVLAYARLSHTFRLKDGVLAYARLSH(L)**	**24.1**	**0.61**	**1847.0286**	**-0.0286**	**208703/9.0**	**Q14690 Q14690**	HUMAN	Protein RRP5 homolog
2	**5**	**(K)GAFSVVRRC(Carbamidomethyl)VKLC(Carbamidomethyl)TGHGAFSVVRRC(Carbamidomethyl)VKLC(Carbamidomethyl)TGH(E)**	**22.6**	**2.0**	**1846.9527**	**0.0473**	**72678/6.9**	**Q13554 Q13554**	HUMAN	Calcium/calmodulin-dependent protein kinase type II subunit beta
3	**6**	**(R)AGADPPDQKNRLMPLSHAGADPPDQKNRLMPLSH(L)**	**21.9**	**3.6**	**1846.9228**	**0.0772**	**33019/8.8**	**Q8NBL3 Q8NBL3**	HUMAN	Transmembrane protein 178A
4	**7**	**(Q)AGPSGLQRVVKPTSITVHAGPSGLQRVVKPTSITVH(D)**	**21.6**	**4.5**	**1847.0498**	**-0.0498**	**133945/5.9**	**Q6PIJ6 Q6PIJ6**	HUMAN	F-box only protein 38
5	**4**	**(E)AGSVSLRMKQVEELYHAGSVSLRMKQVEELYH(S)**	**21.2**	**6.2**	**1846.9480**	**0.0520**	**284542/5.2**	**Q13813 Q13813**	HUMAN	Spectrin alpha chain, non-erythrocytic 1
6	**4**	**(A)QSTDGARTKIGFSEGRHQSTDGARTKIGFSEGRH(A)**	**21.1**	**6.8**	**1846.9154**	**0.0846**	**30633/8.0**	**P0C2W1 P0C2W1**	HUMAN	F-box/SPRY domain-containing protein 1
7	**6**	**(A)AGFNVEKFYRIKTHHAGFNVEKFYRIKTHH(S)**	**20.7**	**9.3**	**1846.9711**	**0.0289**	**47781/6.6**	**Q8IV20 Q8IV20**	HUMAN	Laccase domain-containing protein 1
8	**7**	**(L)AGEPRVIALELLDVKSHAGEPRVIALELLDVKSH(M)**	**20.5**	**11**	**1847.0385**	**-0.0385**	**363394/6.4**	**Q8N2C7 Q8N2C7**	HUMAN	Protein unc-80 homolog
9	**5**	**(E)ELLAVGKFTRTSGETTHELLAVGKFTRTSGETTH(T)**	**20.4**	**12**	**1846.9658**	**0.0342**	**358697/9.5**	**P46013 P46013**	HUMAN	Antigen KI-67
9	**6**	**(N)TEIRM(Oxidation)AVSKVADKMDHTEIRM(Oxidation)AVSKVADKMDH(L)**	**20.4**	**12**	**1846.9150**	**0.0850**	**133631/5.1**	**Q5T1M5 Q5T1M5**	HUMAN	FK506-binding protein 15
10	**3**	**(R)EAC(Carbamidomethyl)NQDALQEAGTFRHEAC(Carbamidomethyl)NQDALQEAGTFRH(T)**	**20.2**	**14**	**1846.8137**	**0.186**	**591413/6.1**	**Q63HN8 Q63HN8**	HUMAN	E3 ubiquitin-protein ligase RNF213
11	**5**	**(G)IYQKILAGKLYFPRHIYQKILAGKLYFPRH(L)**	**19.9**	**18**	**1847.0690**	**-0.0690**	**31709/6.5**	**O43930 O43930**	HUMAN	Putative serine/threonine-protein kinase PRKY
12	**6**	**(E)REAGEVELM(Oxidation)GVLALSKEREAGEVELM(Oxidation)GVLALSKE(E)**	**19.7**	**21**	**1846.9579**	**0.0421**	**114875/4.4**	**Q0VD83 Q0VD83**	HUMAN	Apolipoprotein B receptor

13	**7**	**(T)GAPLGQSEELQPLSQRHGAPLGQSEELQPLSQRH(P)**	**19.6**	**22**	**1846.9406**	**0.0594**	**68482/7.1**	**O95208**	**O95208**	HUMAN	Epsin-2
14	**5**	**(R)LLKHWVSPLKDAM(Oxidation)RHLLKHWVSPLKDAM(Oxidation)RH(L)**	**19.4**	**26**	**1847.0109**	**-0.0109**	**93908/9.0**	**Q96JF6**	**Q96JF6**	HUMAN	Zinc finger protein 594
14	**7**	**(N)RMLMLDGMPAVRVKTERMLMLDGMPAVRVKTE(L)**	**19.4**	**26**	**1846.9700**	**0.0300**	**44240/9.7**	**Q9Y4X4**	**Q9Y4X4**	HUMAN	Krueppel-like factor 12
14	**8**	**(S)AGGFAQIQFNVVM(Oxidation)RRHAGGFAQIQFNVVM(Oxidation)RRH(P)**	**19.4**	**26**	**1846.9493**	**0.0507**	**50292/5.2**	**O95264**	**O95264**	HUMAN	5-hydroxytryptamine receptor 3B
14	**8**	**(S)FQWM(Oxidation)SRVLQAIDSIHFQWM(Oxidation)SRVLQAIDSIH(Q)**	**19.4**	**26**	**1846.9269**	**0.0731**	**170371/8.4**	**Q14689**	**Q14689**	HUMAN	Disco-interacting protein 2 homolog A
14	**8**	**(S)FQWM(Oxidation)SRVLQAIDSIHFQWM(Oxidation)SRVLQAIDSIH(Q)**	**19.4**	**26**	**1846.9269**	**0.0731**	**171493/8.4**	**Q9P265**	**Q9P265**	HUMAN	Disco-interacting protein 2 homolog B
14	**8**	**(S)FQWM(Oxidation)SRVLQAIDSIHFQWM(Oxidation)SRVLQAIDSIH(Q)**	**19.4**	**26**	**1846.9269**	**0.0731**	**170769/7.1**	**Q9Y2E4**	**Q9Y2E4**	HUMAN	Disco-interacting protein 2 homolog C
14	**7**	**(A)GVAEGQMENILQEATKEGVAEGQMENILQEATKE(E)**	**19.4**	**26**	**1846.8851**	**0.115**	**133620/5.5**	**Q13615**	**Q13615**	HUMAN	Myotubularin-related protein 3
15	**4**	**(P)RSLQGKSTTLFSRHTKRSLQGKSTTLFSRHTK(A)**	**19.2**	**31**	**1847.0246**	**-0.0246**	**120840/6.9**	**P53396**	**P53396**	HUMAN	ATP-citrate synthase
16	**7**	**(C)GADIEVKNKAGHTPLLLAGADIEVKNKAGHTPLLLA(I)**	**19.1**	**34**	**1847.0385**	**-0.0385**	**28550/7.0**	**A7E2S9**	**A7E2S9**	HUMAN	Putative ankyrin repeat domain-containing protein 30B-like
17	**6**	**(D)KPESMKEFRLDGVSSHKPESMKEFRLDGVSSH(A)**	**19.0**	**36**	**1846.9116**	**0.0884**	**111652/4.8**	**Q96JQ2**	**Q96JQ2**	HUMAN	Calmin
17	**6**	**(T)PLQQVTSGQGGVFTQYHPLQQVTSGQGGVFTQYH(K)**	**19.0**	**36**	**1846.9082**	**0.0918**	**56805/7.9**	**B2RXH2**	**B2RXH2**	HUMAN	Lysine-specific demethylase 4E
17	**6**	**(V)GSVGQHTGEPVEELALSHGSVGQHTGEPVEELALSH(C)**	**19.0**	**36**	**1846.8930**	**0.107**	**42070/4.8**	**Q9H6Y2**	**Q9H6Y2**	HUMAN	WD repeat-containing protein 55
18	**4**	**(H)AGKASSSMGAPRTHGPSTFAGKASSSMGAPRTHGPSTF(D)**	**18.8**	**43**	**1846.8865**	**0.114**	**111579/6.8**	**Q9UGR2**	**Q9UGR2**	HUMAN	Zinc finger CCCH domain-containing protein 7B
19	**6**	**(W)FGVRKGFPNLGYIQNHFGVRKGFPNLGYIQNH(L)**	**18.7**	**46**	**1846.9711**	**0.0289**	**286792/7.4**	**Q92508**	**Q92508**	HUMAN	Piezo-type mechanosensitive ion channel component 1
20	**5**	**(S)RSETDGTVFRIHTKAERSETDGTVFRIHTKAE(G)**	**18.6**	**50**	**1846.9406**	**0.0594**	**30544/5.8**	**Q9Y3V2**	**Q9Y3V2**	HUMAN	RWD domain-containing protein 3
21	**5**	**(E)AGMWTVKTSSSGRHSVRAGMWTVKTSSSGRHSVR(I)**	**18.5**	**54**	**1846.9341**	**0.0659**	**613395/6.1**	**Q96RW7**	**Q96RW7**	HUMAN	Hemicentin-1
21	**7**	**(K)KNISHDTFGTTYGRIHKNISHDTFGTTYGRIH(M)**	**18.5**	**54**	**1846.9195**	**0.0805**	**35583/10.0**	**Q9H7B2**	**Q9H7B2**	HUMAN	Ribosome production factor 2 homolog
22	**7**	**(F)GAGLTRLPIEDHKRFHGAGLTRLPIEDHKRFH(P)**	**18.4**	**59**	**1847.0035**	**-0.00348**	**159583/5.7**	**Q13075**	**Q13075**	HUMAN	Baculoviral IAP repeat-containing protein 1
