# Supplementary material for: MALDI-TOF peptidomic analysis of serum and post-prostatic massage urine specimens to identify prostate cancer biomarkers
Source: Clin Proteomics. 2018 Jul 25;15:23. doi: 10.1186/s12014-018-9199-8 (PMC6060548; doi:10.1186/s12014-018-9199-8)
Supplement: Supplementary file 14 — Additional file 14: MS-Tag search results. MS-MS spectra, peptide lists and MS-Tag search results (including all the configuration parameter) for the fragmentation patters of the 12 MALDI-TOF/MS serum features. [file 12014_2018_9199_MOESM14_ESM.zip › New folder/1887_0.pdf]

MS-Tag Search Results

Search completed. 12 sec elapsed. 0 sec remaining.

[–] **Parameters**

Database searched: **SwissProt.2016.5.30**
Digest Used: **No enzyme**
Max. # Missed Cleavages: **1**
Constant Modification: **Carbamidomethyl (C)**
Ion Types Considered: **a, a-NH3, a-H2O, b, b-NH3, b-H2O, b+H2O, y, y-NH3, y-H2O, I, i, P, S, M-H2O, M-NH3, M-SOCH4**
Search Mode:
Max Modifications: **2**
Peptide Masses are: **monoisotopic**

[–] **Pre Search Results (SwissProt.2016.5.30)**

Number of entries in the database: **551193**
Full Molecular Weight range: **551193** entries.
Full pI range: **551193** entries.
Taxonomy search **HOMO SAPIENS** selects **20202** entries.
Pre searches select **20202** entries.

Results

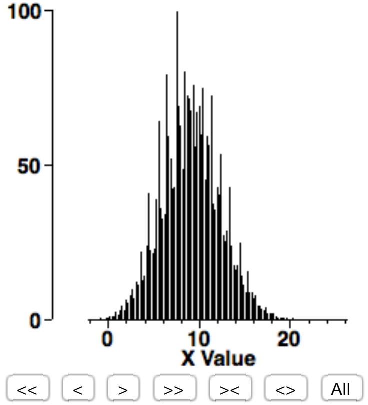

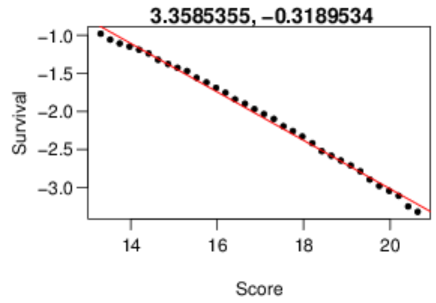

expectation value = 1.41
num peptides considered = 130436
MS-Tag search selects **31** entries (results displayed for top **30** matches).

Parent mass: **1887.0300 (+/- 0.500 Da)**
[–] **Fragment Ions**

35 Ions used in search: **23.0000, 70.1000, 84.1000, 86.1000, 105.7000, 110.1000, 112.1000, 129.1000, 216.1000, 225.1000, 239.1000, 251.1000, 277.2000, 291.2000, 296.2000, 353.1000, 378.1000, 566.4000, 626.3000, 655.6000, 1032.6000, 1055.6000, 1077.6000, 1197.0000, 1218.7000, 1233.8000, 1354.8000, 1370.9000, 1397.9000, 1729.3000, 1757.4000, 1774.4000, 1844.4000, 1858.6000, 1871.2000 (+/- 1.00 Da)**

Rank	# Unmatched Ions	Sequence	Score	Expect	MH$^+$ Calculated (Da)	Error (Da)	Protein MW (Da)/pI	Accession #	Species	Protein Name
1	**15**	**(Y)EVRFHWGRENQRGSEEVRFHWGRENQRGSE(H)**	**26.1**	**1.4**	**1886.9005**	**0.130**	**32973/4.8**	**P35219 P35219**	HUMAN	Carbonic anhydrase-related protein
2	**16**	**(T)LRSGGGPQGQEEASGVSPSSLRSGGGPQGQEEASGVSPSS(L)**	**26.0**	**1.5**	**1886.8839**	**0.146**	**261209/4.4**	**Q8IWN7 Q8IWN7**	HUMAN	Retinitis pigmentosa 1-like 1 protein
3	**17**	**(S)EHSDASPMSINEVILSASEHSDASPMSINEVILSAS(G)**	**25.1**	**2.9**	**1886.8800**	**0.150**	**62890/9.4**	**Q8WY91 Q8WY91**	HUMAN	THAP domain-containing protein 4
4	**15**	**(P)LHQHERYLC(Carbamidomethyl)KMNEELHQHERYLC(Carbamidomethyl)KMNEE(I)**	**24.5**	**4.6**	**1886.8636**	**0.166**	**136449/5.9**	**O60315 O60315**	HUMAN	Zinc finger E-box-binding homeobox 2
5	**14**	**(N)LQAGVPSRFSGSGSGTDYTLQAGVPSRFSGSGSGTDYT(F)**	**23.6**	**8.9**	**1886.8879**	**0.142**	**11902/6.3**	**P01607 P01607**	HUMAN	Ig kappa chain V-I region Rei
5	**15**	**(L)SSNKDHQGRAC(Carbamidomethyl)SSPPC(Carbamidomethyl)VSSNKDHQGRAC(Carbamidomethyl)SSPPC(Carbamidomethyl)V(Y)**	**23.6**	**8.9**	**1886.8232**	**0.207**	**38694/8.8**	**Q8NGC5 Q8NGC5**	HUMAN	Olfactory receptor 6J1
6	**14**	**(S)AVGTPLNALGSPYRVITSAAVGTPLNALGSPYRVITSA(M)**	**23.0**	**14**	**1887.0334**	**-0.00344**	**50872/7.5**	**P48443 P48443**	HUMAN	Retinoic acid receptor RXR-gamma
7	**14**	**(I)SSVHRSRHLVMPEHQSSSVHRSRHLVMPEHQS(R)**	**22.8**	**16**	**1886.9402**	**0.0898**	**72709/7.3**	**Q96NU1 Q96NU1**	HUMAN	Sterile alpha motif domain-containing protein 11
8	**15**	**(G)AGAARASGPGGGAPRGPHGGAASAGAARASGPGGGAPRGPHGGAAS(G)**	**22.5**	**20**	**1886.9328**	**0.0972**	**17993/8.8**	**P78358 P78358**	HUMAN	Cancer/testis antigen 1
9	**17**	**(D)LAPALAEDGPPTVAPGPVQSLAPALAEDGPPTVAPGPVQS(P)**	**22.4**	**21**	**1886.9858**	**0.0442**	**178676/6.4**	**O95785 O95785**	HUMAN	Protein Wiz
9	**17**	**(T)IYLVIKSKQGSRSLAHSIYLVIKSKQGSRSLAHS(F)**	**22.4**	**21**	**1887.0811**	**-0.0511**	**52897/5.7**	**Q8IYU4 Q8IYU4**	HUMAN	Ubiquilin-like protein
9	**15**	**(L)LGMHKKSMGMIRKPGSELGMHKKSMGMIRKPGSE(D)**	**22.4**	**21**	**1886.9761**	**0.0539**	**38432/9.0**	**Q9NZR4 Q9NZR4**	HUMAN	Visual system

											homeobox 1
9	**16**	**(Q)LRIQYGTSKGKNYTEELRIQYGTSKGKNYTEE(E)**	**22.4**	**21**	**1886.9607**	**0.0693**	**122606/8.3**	**P28370**	**P28370**	HUMAN	Probable global transcription activator SNF2L1
9	**14**	**(D)EPPSSVSHGAKASTTSLSGSEPPSSVSHGAKASTTSLSGS(D)**	**22.4**	**21**	**1886.9090**	**0.121**	**95322/6.6**	**Q8N1G2**	**Q8N1G2**	HUMAN	Cap-specific mRNA (nucleoside-2'-O-)-methyltransferase 1
9	**16**	**(D)ISPQGVSDDSSTGSRVHASISPQGVSDDSSTGSRVHAS(R)**	**22.4**	**21**	**1886.8839**	**0.146**	**216043/5.9**	**Q3V6T2**	**Q3V6T2**	HUMAN	Girdin
10	**17**	**(G)IYLSLSLKNPLKKVLASIYLSLSLKNPLKKVLAS(S)**	**22.3**	**23**	**1887.1677**	**-0.138**	**219609/5.7**	**Q6YHU6**	**Q6YHU6**	HUMAN	Thyroid adenoma-associated protein
11	**18**	**(E)LPPTSTATSRSPPESKGSSLPPTSTATSRSPPESKGSS(R)**	**22.0**	**29**	**1886.9454**	**0.0846**	**351973/8.4**	**Q96N23**	**Q96N23**	HUMAN	Cilia- and flagella-associated protein 54
12	**15**	**(G)LARIVDQHYSHKGYLSLARIVDQHYSHKGYLS(E)**	**21.9**	**31**	**1886.9872**	**0.0428**	**65922/5.3**	**P31152**	**P31152**	HUMAN	Mitogen-activated protein kinase 4
12	**14**	**(K)IC(Carbamidomethyl)LEIM(Oxidation)QRTGAHLELSIC(Carbamidomethyl)LEIM(Oxidation)QRTGAHLELS(L)**	**21.9**	**31**	**1886.9463**	**0.0837**	**141457/6.4**	**Q00341**	**Q00341**	HUMAN	Vigilin
12	**16**	**(D)SAPERKSPSHHRQPSDASAPERKSPSHHRQPSDA(S)**	**21.9**	**31**	**1886.9216**	**0.108**	**167706/5.3**	**O15085**	**O15085**	HUMAN	Rho guanine nucleotide exchange factor 11
13	**16**	**(S)LHC(Carbamidomethyl)QERANELMRAMKLHC(Carbamidomethyl)QERANELMRAMK(G)**	**21.7**	**36**	**1886.9146**	**0.115**	**83369/6.4**	**Q33E94**	**Q33E94**	HUMAN	Transcription factor RFX4
13	**17**	**(S)TGHGRGHESSMKKLMAC(Carbamidomethyl)TGHGRGHESSMKKLMAC(Carbamidomethyl)(V)**	**21.7**	**36**	**1886.8782**	**0.152**	**11232/4.6**	**Q9NNZ6**	**Q9NNZ6**	HUMAN	Protamine-3
14	**17**	**(L)LHKHIEVANGPASHFETLHKHIEVANGPASHFET(R)**	**21.5**	**41**	**1886.9508**	**0.0792**	**61653/6.0**	**Q92558**	**Q92558**	HUMAN	Wiskott-Aldrich syndrome protein family member 1
14	**16**	**(P)QYRAHELGMKLAHGFEQYRAHELGMKLAHGFE(I)**	**21.5**	**41**	**1886.9330**	**0.0970**	**72759/4.7**	**O95905**	**O95905**	HUMAN	Protein ecdysoneless homolog
15	**14**	**(T)IHTGVVYPILSRSLRQM(Oxidation)IHTGVVYPILSRSLRQM(Oxidation)(A)**	**21.4**	**45**	**1887.0269**	**0.00309**	**43904/5.6**	**Q9NZN9**	**Q9NZN9**	HUMAN	Aryl-hydrocarbon-interacting protein-like 1
16	**15**	**(S)LELPMAM(Oxidation)RFRHLKKTLELPMAM(Oxidation)RFRHLKKT(S)**	**21.3**	**48**	**1887.0455**	**-0.0155**	**293517/8.6**	**Q9UMN6**	**Q9UMN6**	HUMAN	Histone-lysine N-methyltransferase 2B
16	**15**	**(E)LERVLQAPPPDVGNGEVPLERVLQAPPPDVGNGEVP(K)**	**21.3**	**48**	**1886.9971**	**0.0329**	**163822/4.9**	**Q96T23**	**Q96T23**	HUMAN	Remodeling and spacing factor 1
16	**17**	**(V)PPTATEGLSTSVQPTAGEGSPPTATEGLSTSVQPTAGEGS(S)**	**21.3**	**48**	**1886.8978**	**0.132**	**103255/5.2**	**Q9HCI5**	**Q9HCI5**	HUMAN	Melanoma-associated antigen E1
17	**14**	**(L)LFHGTLQLGQALNGVYRLFHGTLQLGQALNGVYR(T)**	**21.2**	**52**	**1887.0235**	**0.00646**	**22106/7.1**	**Q6UXH0**	**Q6UXH0**	HUMAN	Angiopoietin-like protein 8
17	**16**	**(K)LKKPVAFSDYIHPVC(Carbamidomethyl)LLKKPVAFSDYIHPVC(Carbamidomethyl)L(P)**	**21.2**	**52**	**1887.0197**	**0.0103**	**70038/5.6**	**P00734**	**P00734**	HUMAN	Prothrombin
