# Supplementary material for: MALDI-TOF peptidomic analysis of serum and post-prostatic massage urine specimens to identify prostate cancer biomarkers
Source: Clin Proteomics. 2018 Jul 25;15:23. doi: 10.1186/s12014-018-9199-8 (PMC6060548; doi:10.1186/s12014-018-9199-8)
Supplement: Supplementary file 14 — Additional file 14: MS-Tag search results. MS-MS spectra, peptide lists and MS-Tag search results (including all the configuration parameter) for the fragmentation patters of the 12 MALDI-TOF/MS serum features. [file 12014_2018_9199_MOESM14_ESM.zip › New folder/1896_01.pdf]

MS-Tag Search Results

Search completed. 13 sec elapsed. 0 sec remaining.

[–] **Parameters**

Database searched: **SwissProt.2016.5.30**
Digest Used: **No enzyme**
Max. # Missed Cleavages: **1**
Constant Modification: **Carbamidomethyl (C)**
Ion Types Considered: **a, a-NH3, a-H2O, b, b-NH3, b-H2O, b+H2O, y, y-NH3, y-H2O, I, i, P, S, M-H2O, M-NH3, M-SOCH4**
Search Mode:
Max Modifications: **2**
Peptide Masses are: **monoisotopic**

[–] **Pre Search Results (SwissProt.2016.5.30)**

Number of entries in the database: **551193**
Full Molecular Weight range: **551193** entries.
Full pI range: **551193** entries.
Taxonomy search **HOMO SAPIENS** selects **20202** entries.
Pre searches select **20202** entries.

Results

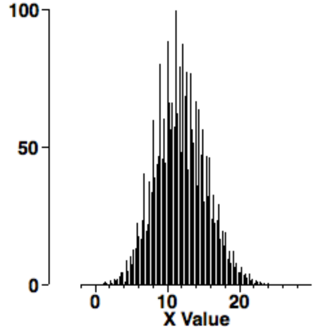

<< < > >> >< <> All

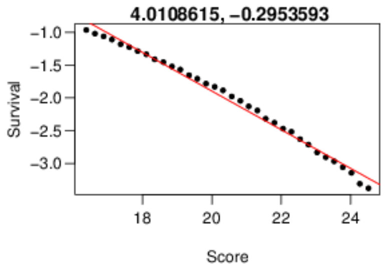

expectation value = 2.06
num peptides considered = 127226
MS-Tag search selects **33** entries (results displayed for top **30** matches).

Parent mass: **1896.0100 (+/- 0.500 Da)**
[–] **Fragment Ions**

33 Ions used in search: **70.1000, 74.1000, 84.1000, 86.1000, 101.1000, 110.1000, 112.1000, 120.1000, 129.1000, 136.1000, 172.1000, 175.1000, 181.1000, 184.1000, 185.1000, 209.1000, 225.1000, 242.1000, 296.1000, 319.1000, 572.3000, 669.3000, 686.4000, 783.4000, 1327.7000, 1481.1000, 1694.5000, 1723.5000, 1740.5000, 1765.6000, 1782.6000, 1836.5000, 1854.6000 (+/- 1.00 Da)**

Rank	# Unmatched Ions	Sequence	Score	Expect	MH^+ Calculated (Da)	Error (Da)	Protein MW (Da)/pI	Accession #	
1	8	(R)NGFKSHALQLNNRQIRNGFKSHALQLNNRQIR(G)	29.8	2.1	1896.0311	-0.0211	192787/6.7	P0C0L4 P0C0L4	[illegible]
1	8	(R)NGFKSHALQLNNRQIRNGFKSHALQLNNRQIR(G)	29.8	2.1	1896.0311	-0.0211	192753/6.9	P0C0L5 P0C0L5	[illegible]
2	8	(Y)LSLM(Oxidation)HAIVRTTPYLQHLSLM(Oxidation)HAIVRTTPYLQH(R)	27.1	13	1896.0160	-0.00601	78961/5.9	Q9NZQ3 Q9NZQ3	[illegible]
3	11	(N)NLTEDNPNLSMAQRRHNLTEDNPNLSMAQRRH(K)	26.8	16	1895.9141	0.0959	119659/6.2	Q9BQF6 Q9BQF6	[illegible]
4	10	(H)IVTLKNGRVVMLVIFGHIVTLKNGRVVMLVIFGH(C)	26.5	19	1896.1252	-0.115	158539/7.2	O75882 O75882	[illegible]
5	8	(V)LGFGALTPTSPQSSHPDSPLGFGALTPTSPQSSHPDSP(E)	26.2	24	1895.9134	0.0966	74855/9.5	Q96BD5 Q96BD5	[illegible]
6	11	(L)LATIQEHGYPTINLGIVGLATIQEHGYPTINLGIVG(D)	26.1	25	1896.0225	-0.0125	79749/5.2	Q9NQX3 Q9NQX3	[illegible]
6	12	(N)IGFPGPKGPTGDPGKNGDKGIGFPGPKGPTGDPGKNGDKG(H)	26.1	25	1895.9610	0.0490	129315/9.1	P08123 P08123	[illegible]
7	10	(D)INIC(Carbamidomethyl)PQDC(Carbamidomethyl)LRGSIVGGHINIC(Carbamidomethyl)PQDC(Carbamidomethyl)LRGSIVGGH(E)	26.0	27	1895.9215	0.0885	124320/6.2	P07949 P07949	[illegible]
8	10	(V)LKRKRTPQSDLKGKIKLKRKRTPQSDLKGKIK(G)	25.9	29	1896.1865	-0.177	140743/8.1	A6NM62 A6NM62	[illegible]
9	10	(L)LAERDLERAEVAKATSHLAERDLERAEVAKATSH(V)	25.8	31	1895.9934	0.0166	162248/5.3	P30622 P30622	[illegible]
9	10	(L)LAERDLERAEVAKATSHLAERDLERAEVAKATSH(I)	25.8	31	1895.9934	0.0166	115838/6.3	Q9UDT6 Q9UDT6	[illegible]
9	11	(A)LAC(Carbamidomethyl)ASPFPEEAPGPGGAGGPGLAC(Carbamidomethyl)ASPFPEEAPGPGGAGGPG(G)	25.8	31	1895.8592	0.151	143753/8.7	O15399 O15399	[illegible]
10	12	(S)NASSSILPTQDPNLKPTINASSSILPTQDPNLKPTI(D)	25.7	33	1896.0073	0.00272	241965/8.2	A0AVI2 A0AVI2	[illegible]
10	11	(Y)ILGNSSDAQLLRDTFVFILGNSSDAQLLRDTFVF(K)	25.7	33	1895.9862	0.0238	116012/9.0	Q8NEM8 Q8NEM8	[illegible]
10	12	(P)RTRVGAADGLVLDVLGQRRTRVGAADGLVLDVLGQR(R)	25.7	33	1896.0774	-0.0674	59373/7.6	Q8TC41 Q8TC41	[illegible]
11	12	(P)IDM(Oxidation)SPNIETPLRPKLRIDM(Oxidation)SPNIETPLRPKLR(K)	25.6	36	1896.0371	-0.0271	119532/9.5	Q9C0D4 Q9C0D4	[illegible]
11	8	(V)PQEALDKLLSFTHKLRPQEALDKLLSFTHKLR(E)	25.6	36	1896.0702	-0.0602	214826/7.0	A3KMH1 A3KMH1	[illegible]
11	12	(G)GDNEPDSEKQSHRRKLGDNEPDSEKQSHRRKL(H)	25.6	36	1895.9318	0.0782	868491/5.7	Q5VST9 Q5VST9	[illegible]
12	9	(G)IDLRSGAMTPDHWIKRIDLRSGAMTPDHWIKR(G)	25.5	38	1895.9909	0.0191	507703/6.0	Q9P225 Q9P225	[illegible]

13	**10**	**(S)IDSGVELTTSPKNVPTHTIDSGVELTTSPKNVPTHT(N)**	**25.3**	**44**	**1895.9709**	**0.0391**	**57394/7.9**	**O15403 O15403**	[illegible]
14	**12**	**(P)LAPSDLGLSRPM(Oxidation)PEPKATLAPSDLGLSRPM(Oxidation)PEPKAT(G)**	**25.1**	**50**	**1895.9895**	**0.0205**	**106058/6.4**	**Q9UHL9 Q9UHL9**	[illegible]
15	**12**	**(G)LALTTM(Oxidation)PTVLGVLWLDHLALTTM(Oxidation)PTVLGVLWLDH(R)**	**25.0**	**54**	**1896.0299**	**-0.0199**	**35241/8.6**	**Q9H339 Q9H339**	[illegible]
15	**12**	**(S)LLTELVLRRISASC(Carbamidomethyl)QHLLTELVLRRISASC(Carbamidomethyl)QH(P)**	**25.0**	**54**	**1896.0484**	**-0.0384**	**52429/7.0**	**Q9ULA0 Q9ULA0**	[illegible]
15	**10**	**(G)LGAHFTVPLKQVSC(Carbamidomethyl)MAHLGAHFTVPLKQVSC(Carbamidomethyl)MAH(R)**	**25.0**	**54**	**1895.9619**	**0.0481**	**22049/11.0**	**A0PJW6 A0PJW6**	[illegible]
15	**12**	**(R)NSTIEAANLAGLKILRVINSTIEAANLAGLKILRVI(N)**	**25.0**	**54**	**1896.1277**	**-0.118**	**51928/5.5**	**P48723 P48723**	[illegible]
15	**12**	**(G)SHALC(Carbamidomethyl)TC(Carbamidomethyl)C(Carbamidomethyl)FQPM(Oxidation)PDRSHALC(Carbamidomethyl)TC(Carbamidomethyl)C(Carbamidomethyl)FQPM(Oxidation)PDR(R)**	**25.0**	**54**	**1895.7656**	**0.244**	**73387/5.7**	**Q96EP1 Q96EP1**	[illegible]
16	**11**	**(E)NPDEKSIITYVVAFYHNPDEKSIITYVVAFYH(Y)**	**24.9**	**58**	**1895.9538**	**0.0562**	**246470/5.1**	**P11277 P11277**	[illegible]
17	**12**	**(L)LALGEAREEQNIIFRHLALGEAREEQNIIFRH(N)**	**24.8**	**62**	**1896.0086**	**0.00138**	**144035/5.4**	**Q9UQP3 Q9UQP3**	[illegible]
17	**10**	**(A)GVQGGSELPLGSQLRVPTTGVQGGSELPLGSQLRVPTT(P)**	**24.8**	**62**	**1896.0185**	**-0.00852**	**19206/10.5**	**Q569G3 Q569G3**	[illegible]
17	**11**	**(S)LTPSIGPVSRGMKESISHLTPSIGPVSRGMKESISH(K)**	**24.8**	**62**	**1896.0008**	**0.00924**	**533649/6.2**	**Q96M86 Q96M86**	[illegible]
17	**12**	**(T)TALEGPELTIQNTTAHSLTALEGPELTIQNTTAHSL(L)**	**24.8**	**62**	**1895.9709**	**0.0391**	**295222/5.9**	**Q02388 Q02388**	[illegible]
