# Supplementary material for: MALDI-TOF peptidomic analysis of serum and post-prostatic massage urine specimens to identify prostate cancer biomarkers
Source: Clin Proteomics. 2018 Jul 25;15:23. doi: 10.1186/s12014-018-9199-8 (PMC6060548; doi:10.1186/s12014-018-9199-8)
Supplement: Supplementary file 14 — Additional file 14: MS-Tag search results. MS-MS spectra, peptide lists and MS-Tag search results (including all the configuration parameter) for the fragmentation patters of the 12 MALDI-TOF/MS serum features. [file 12014_2018_9199_MOESM14_ESM.zip › New folder/1977_97.pdf]

MS-Tag Search Results

Search completed. 13 sec elapsed. 0 sec remaining.

[–] **Parameters**

Database searched: **SwissProt.2016.5.30**
Digest Used: **No enzyme**
Max. # Missed Cleavages: **1**
Constant Modification: **Carbamidomethyl (C)**
Ion Types Considered: **a, a-NH3, a-H2O, b, b-NH3, b-H2O, b+H2O, y, y-NH3, y-H2O, I, i, P, S, M-H2O, M-NH3, M-SOCH4**
Search Mode:
Max Modifications: **2**
Peptide Masses are: **monoisotopic**

[–] **Pre Search Results (SwissProt.2016.5.30)**

Number of entries in the database: **551193**
Full Molecular Weight range: **551193** entries.
Full pI range: **551193** entries.
Taxonomy search **HOMO SAPIENS** selects **20202** entries.
Pre searches select **20202** entries.

Results

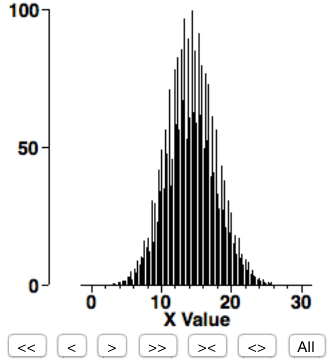

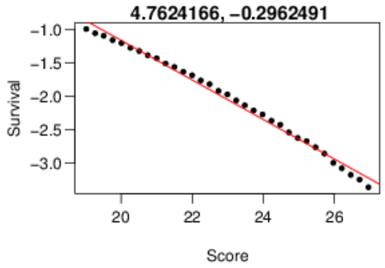

expectation value = 3.99
num peptides considered = 129203
MS-Tag search selects **32** entries (results displayed for top **30** matches).

Parent mass: **1978.1000 (+/- 0.500 Da)**
[–] **Fragment Ions**

37 Ions used in search: **69.7000, 83.6000, 84.1000, 85.6000, 86.1000, 98.5000, 100.5000, 104.1000, 109.4000, 110.1000, 119.3000, 120.1000, 128.2000, 129.1000, 148.2000, 162.7000, 167.6000, 180.2000, 194.6000, 205.1000, 215.1000, 216.0000, 221.0000, 263.0000, 275.1000, 327.3000, 330.6000, 331.7000, 336.0000, 345.7000, 399.7000, 404.6000, 428.2000, 439.1000, 665.8000, 752.2000, 1777.9000 (+/- 1.00 Da)**

Rank	# Unmatched Ions	Sequence	Score	Expect	MH^+ Calculated (Da)	Error (Da)	Protein MW (Da)/pI	Accession #	Species	Pro
1	**12**	**(Q)AGSQHGQSESIVPERHGTTAGSQHGQSESIVPERHGTT(H)**	**31.3**	**4.0**	**1977.9373**	**0.163**	**248074/8.5**	**Q5D862 Q5D862**	HUMAN	Fila
1	**9**	**(R)TNSM(Oxidation)SKEAFTPEQLHLETNSM(Oxidation)SKEAFTPEQLHLE(K)**	**31.3**	**4.0**	**1977.9222**	**0.178**	**8554/6.4**	**B1AMM8 B1AMM8**	HUMAN	Puta unc prot by I
2	**13**	**(P)LTLALFVGAGHAQRDPVGRLTLALFVGAGHAQRDPVGR(Y)**	**30.6**	**6.4**	**1978.0981**	**0.00190**	**195054/5.1**	**Q14767 Q14767**	HUMAN	Late tran grov beta prot
3	**9**	**(N)HPTQNPPAGLMSMPNALTTHPTQNPPAGLMSMPNALTT(Q)**	**30.3**	**7.9**	**1977.9521**	**0.148**	**44102/5.5**	**Q9GZV5 Q9GZV5**	HUMAN	WW con tran regu
4	**12**	**(I)AQITGPPDRC(Carbamidomethyl)QHAAEIITAQITGPPDRC(Carbamidomethyl)QHAAEIIT(D)**	**29.7**	**12**	**1977.9811**	**0.119**	**67561/7.2**	**Q96AE4 Q96AE4**	HUMAN	Far eler prot
5	**11**	**(S)KKASGSGGGSAALGPSGFGPSGGSKKASGSGGGSAALGPSGFGPSGGS(G)**	**29.4**	**15**	**1977.9625**	**0.138**	**88062/9.1**	**P25440 P25440**	HUMAN	Bro con 2
6	**13**	**(P)LTIEDPKHVC(Carbamidomethyl)DPPSGPDTLTIEDPKHVC(Carbamidomethyl)DPPSGPDT(T)**	**29.3**	**16**	**1977.9222**	**0.178**	**97337/5.3**	**P32927 P32927**	HUMAN	Cyt con beta
7	**9**	**(K)GFLHTWAGMSKPSSKNTEGFLHTWAGMSKPSSKNTE(K)**	**29.1**	**18**	**1977.9487**	**0.151**	**69770/9.3**	**Q9H967 Q9H967**	HUMAN	WD con 76
8	**11**	**(V)LERHESPDIEDFSFKELERHESPDIEDFSFKE(P)**	**28.9**	**21**	**1977.9189**	**0.181**	**94113/9.4**	**Q9HCG1 Q9HCG1**	HUMAN	Zin prot
9	**11**	**(A)KSEILETHGTMNFLGAETKSEILETHGTMNFLGAET(K)**	**28.8**	**22**	**1977.9586**	**0.141**	**59090/8.4**	**P13682 P13682**	HUMAN	Zin prot
9	**12**	**(R)SKHPSGSNVSFSRDTEGGESKHPSGSNVSFSRDTEGGE(E)**	**28.8**	**22**	**1977.8897**	**0.210**	**151152/6.0**	**Q12767 Q12767**	HUMAN	Tra prot
10	**12**	**(E)GASQSNNFRYTC(Carbamidomethyl)DIC(Carbamidomethyl)GKGASQSNNFRYTC(Carbamidomethyl)DIC(Carbamidomethyl)GK(K)**	**28.7**	**24**	**1977.8542**	**0.246**	**104956/6.7**	**Q5T7W0 Q5T7W0**	HUMAN	Zin prot
11	**8**	**(H)PIVQRFAAHLIAGAPSEGSGPIVQRFAAHLIAGAPSEGSG(A)**	**28.6**	**25**	**1978.0505**	**0.0495**	**92549/9.2**	**Q8WTT2 Q8WTT2**	HUMAN	Nuc con hon
12	**11**	**(Q)KMANNPGTPFEGVLM(Oxidation)LRSKMANNPGTPFEGVLM(Oxidation)LRS(S)**	**28.5**	**27**	**1977.9885**	**0.112**	**81524/9.0**	**Q8TDW5 Q8TDW5**	HUMAN	Syn like
13	**11**	**(E)HPPLMLQNRTVVGAAATTTHPPLMLQNRTVVGAAATTT(T)**	**28.4**	**29**	**1978.0539**	**0.0461**	**130844/8.5**	**Q86Z02 Q86Z02**	HUMAN	Hor inte kina
13	**12**	**(E)AKMPRFGHGTFLLC(Carbamidomethyl)LETAKMPRFGHGTFLLC(Carbamidomethyl)LET(I)**	**28.4**	**29**	**1978.0037**	**0.0963**	**46322/8.4**	**Q9BXW7 Q9BXW7**	HUMAN	Cat criti prot
14	**14**	**(N)AGSLTM(Oxidation)SEKREFPTVPLVAGSLTM(Oxidation)SEKREFPTVPLV(K)**	**28.2**	**33**	**1978.0314**	**0.0686**	**56941/8.2**	**Q16851 Q16851**	HUMAN	UTI pho urid
14	**13**	**(T)TNPHMNQSSNYLKQSKTTNPHMNQSSNYLKQSKT(L)**	**28.2**	**33**	**1977.9447**	**0.155**	**84569/9.7**	**Q5T3J3 Q5T3J3**	HUMAN	Liga nuc inte 1

15	12	(Q)M(Oxidation)NNPVGLPQHFSPQSLPVM(Oxidation)NNPVGLPQHFSPQSLPV(Q)	28.1	35	1977.9851	0.115	541374/6.1	Q8NEZ4	Q8NEZ4	HUMAN	His met 2C
15	14	(G)SQDAGIWYLFHKEPTGESQDAGIWYLFHKEPTGE(S)	28.1	35	1977.9341	0.166	154375/8.4	Q9UHN6	Q9UHN6	HUMAN	Tra prot
15	9	(D)SGAIC(Carbamidomethyl)TC(Carbamidomethyl)THGKLSC(Carbamidomethyl)IGGQASGAIC(Carbamidomethyl)TC(Carbamidomethyl)THGKLSC(Carbamidomethyl)IGGQA(P)	28.1	35	1977.8939	0.206	585575/6.7	P98088	P98088	HUMAN	Mu
16	12	(G)QPGRSSSLTGVSRLAGGSC(Carbamidomethyl)TQPGRSSSLTGVSRLAGGSC(Carbamidomethyl)T(K)	28.0	38	1977.9771	0.123	132609/8.3	Q9P2F6	Q9P2F6	HUMAN	Rho acti 20
16	11	(R)QESSTTFVSDGSLEKHPEQESSTTFVSDGSLEKHPE(V)	28.0	38	1977.9036	0.196	372823/4.4	P13611	P13611	HUMAN	Ver prot
17	15	(P)GASDRPQPTAMNSIVM(Oxidation)ETGGASDRPQPTAMNSIVM(Oxidation)ETG(N)	27.9	41	1977.9005	0.200	57194/9.2	O43660	O43660	HUMAN	Plei regu
17	12	(W)DVSHQPETADTAHGVEREDVSHQPETADTAHGVERE(T)	27.9	41	1977.8897	0.210	242969/8.5	Q5JSZ5	Q5JSZ5	HUMAN	Pro
18	12	(P)SLQRRAGSRSDVTHHAVTSLQRRAGSRSDVTHHAVT(S)	27.8	43	1978.0325	0.0675	289387/5.2	Q9ULT8	Q9ULT8	HUMAN	E3 prot HE
19	14	(S)SQFTRNALPALLIYKGGESQFTRNALPALLIYKGGE(L)	27.7	47	1978.0756	0.0244	34282/4.7	Q13371	Q13371	HUMAN	Pho prot
19	11	(P)SQTVSRNFTSVDHGISKDSQTVSRNFTSVDHGISKD(K)	27.7	47	1977.9625	0.138	114193/5.8	Q15431	Q15431	HUMAN	Syn com
19	13	(T)TTKLSMLELHGNPFEC(Carbamidomethyl)TTTKLSMLELHGNPFEC(Carbamidomethyl)T(C)	27.7	47	1977.9409	0.159	119829/6.2	Q9NR97	Q9NR97	HUMAN	Toll 8
19	12	(D)SLDAFMSEM(Oxidation)KSGSTLDGVSSLDAFMSEM(Oxidation)KSGSTLDGVS(R)	27.7	47	1977.8780	0.222	88815/5.1	Q9BWU0	Q9BWU0	HUMAN	Kar
