# Supplementary material for: MALDI-TOF peptidomic analysis of serum and post-prostatic massage urine specimens to identify prostate cancer biomarkers
Source: Clin Proteomics. 2018 Jul 25;15:23. doi: 10.1186/s12014-018-9199-8 (PMC6060548; doi:10.1186/s12014-018-9199-8)
Supplement: Supplementary file 14 — Additional file 14: MS-Tag search results. MS-MS spectra, peptide lists and MS-Tag search results (including all the configuration parameter) for the fragmentation patters of the 12 MALDI-TOF/MS serum features. [file 12014_2018_9199_MOESM14_ESM.zip › New folder/3156.3.pdf]

MS-Tag Search Results

Search completed. 14 sec elapsed. 0 sec remaining.

[–] **Parameters**

Database searched: **SwissProt.2016.5.30**
Digest Used: **No enzyme**
Max. # Missed Cleavages: **1**
Constant Modification: **Carbamidomethyl (C)**
Ion Types Considered: **a, a-NH3, a-H2O, b, b-NH3, b-H2O, b+H2O, y, y-NH3, y-H2O, I, i, P, S, M-H2O, M-NH3, M-SOCH4**
Search Mode:
Max Modifications: **2**
Peptide Masses are: **monoisotopic**

[–] **Pre Search Results (SwissProt.2016.5.30)**

Number of entries in the database: **551193**
Full Molecular Weight range: **551193** entries.
Full pI range: **551193** entries.
Taxonomy search **HOMO SAPIENS** selects **20202** entries.
Pre searches select **20202** entries.

Results

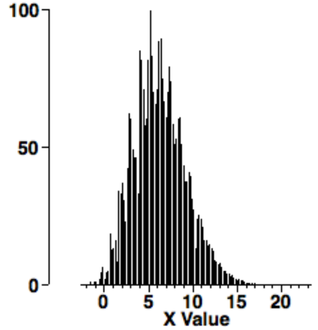

<< < > >> >< <> All

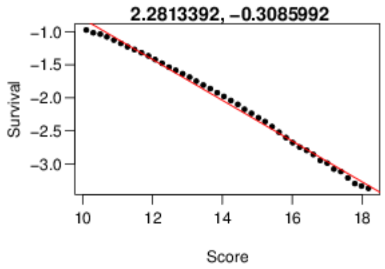

expectation value = 1.67
num peptides considered = 145235
MS-Tag search selects **32** entries (results displayed for top **30** matches).

Parent mass: **3156.3200 (+/- 0.500 Da)**
[–] **Fragment Ions**

37 Ions used in search: **25.0000, 83.7000, 85.8000, 110.1000, 129.1000, 135.2000, 272.1000, 333.0000, 351.0000, 664.0000, 951.3000, 1098.2000, 1115.2000, 1202.8000, 1230.5000, 1327.3000, 1385.6000, 1427.6000, 1475.5000, 1486.3000, 1604.1000, 1613.5000, 1641.3000, 1678.7000, 1714.3000, 1890.7000, 2020.5000, 2057.1000, 2184.9000, 2259.4000, 2463.5000, 2495.3000, 2784.3000, 2899.3000, 3004.3000, 3095.5000, 3114.2000 (+/- 1.00 Da)**

Rank	# Unmatched Ions	Sequence
1	**21**	**(T)VASSKPSSARKTPESFLGPNAALVNLDSLVTVASSKPSSARKTPESFLGPNAALVNLDSLVT(R)**
2	**19**	**(T)YDTTLVEKEGIHVLDWPFDDGAPPSNQIYDTTLVEKEGIHVLDWPFDDGAPPSNQI(V)**
3	**19**	**(I)STIAATVTAPHSESSLSTGPAPAAMATTSSKPESTIAATVTAPHSESSLSTGPAPAAMATTSSKPE(G)**
4	**19**	**(A)DPHFFTQVFQDLKNEGEAAASGGGPPSGGAPADPHFFTQVFQDLKNEGEAAASGGGPPSGGAPA(A)**
5	**20**	**(A)QEWKC(Carbamidomethyl)AFM(Oxidation)ETSAKM(Oxidation)NYNVKELFQELQEWKC(Carbamidomethyl)AFM(Oxidation)ETSAKM(Oxidation)NYNVKELFQEL(L)**
5	**19**	**(V)AGPTTRGAAVSAEAM(Oxidation)EPELADTSALKAAPYGHAGPTTRGAAVSAEAM(Oxidation)EPELADTSALKAAPYGH(S)**
5	**20**	**(H)PAAPSAAQANAILAPVKSNPVVSQTLSPGFVGPPAAPSAAQANAILAPVKSNPVVSQTLSPGFVGP(N)**
6	**21**	**(A)KTQTYRPLSKSHSDNSPNAFKDASSPVPPKTQTYRPLSKSHSDNSPNAFKDASSPVPP(P)**
6	**23**	**(A)FLLYTIRRGPAGIGRTGRGM(Oxidation)GGLFSVGETTFLLYTIRRGPAGIGRTGRGM(Oxidation)GGLFSVGETT(A)**
7	**19**	**(F)EEPPEAASSALALM(Oxidation)NSVKTQLHM(Oxidation)ALERNSEEPPEAASSALALM(Oxidation)NSVKTQLHM(Oxidation)ALERNS(W)**
8	**19**	**(E)EKNGPPVASALPPGGAKGPVEEEWPEPSSGEGEKNGPPVASALPPGGAKGPVEEEWPEPSSGEG(Q)**
9	**19**	**(Q)KAAITHEGRTALRPHATDDFNHGVVLSSRKAAITHEGRTALRPHATDDFNHGVVLSSR(A)**
10	**22**	**(S)VTGAAPYSSFM(Oxidation)QAPEQEM(Oxidation)VQVFIPAQAVGAVTGAAPYSSFM(Oxidation)QAPEQEM(Oxidation)VQVFIPAQAVGA(I)**
10	**21**	**(L)YILNSC(Carbamidomethyl)SLSVEHIQSLNTNMHTQPLKEYILNSC(Carbamidomethyl)SLSVEHIQSLNTNMHTQPLKE(A)**
11	**21**	**(K)PSFLGVNESPSSQVSSSSSSSSPAKSHNSPHGPSFLGVNESPSSQVSSSSSSSSPAKSHNSPHG(C)**
12	**21**	**(S)PC(Carbamidomethyl)QDVKQLTSGVAMAQVLHQIDAAWFNEPC(Carbamidomethyl)QDVKQLTSGVAMAQVLHQIDAAWFNE(S)**
12	**21**	**(P)IRSPALIGC(Carbamidomethyl)FIVDRQYFQEIGLLDEGM(Oxidation)IRSPALIGC(Carbamidomethyl)FIVDRQYFQEIGLLDEGM(Oxidation)(E)**
12	**20**	**(D)SPRPSAPDM(Oxidation)EDFGLVKLPHPAAPVTVKRKSPRPSAPDM(Oxidation)EDFGLVKLPHPAAPVTVKRK(R)**
12	**22**	**(V)PVPGPKSHPSASSGKLLLPQFGMVIILISVSPVPGPKSHPSASSGKLLLPQFGMVIILISVS(A)**
13	**22**	**(K)TGVKKNAGTDANVFITLFGTQDDTGMTLLKTGVKKNAGTDANVFITLFGTQDDTGMTLLK(S)**
14	**21**	**(Q)EEVFRNLDAQYEM(Oxidation)ARSQTHTQRGM(Oxidation)GLGEEVFRNLDAQYEM(Oxidation)ARSQTHTQRGM(Oxidation)GLG(F)**
14	**22**	**(K)HM(Oxidation)NSAGVLATLRNYDYYASRVPESIKNAHM(Oxidation)NSAGVLATLRNYDYYASRVPESIKNA(L)**
14	**22**	**(R)KRKLLAEVTLKTYWVSKGSAFSTSISKQKRKLLAEVTLKTYWVSKGSAFSTSISKQ(E)**
15	**22**	**(I)M(Oxidation)C(Carbamidomethyl)PVC(Carbamidomethyl)DKYC(Carbamidomethyl)PFM(Oxidation)RLSDSC(Carbamidomethyl)VYAKVTHM(Oxidation)C(Carbamidomethyl)PVC(Carbamidomethyl)DKYC(Carbamidomethyl)PFM(Oxidation)RLSDSC(Carbam**
15	**21**	**(P)LLQGTNSQWESPHGFSGQSKPDLTAGPMWLLQGTNSQWESPHGFSGQSKPDLTAGPMW(P)**
15	**24**	**(K)KEQDTSAHLERMKKNMEQTVKDLQLRKEQDTSAHLERMKKNMEQTVKDLQLR(L)**
15	**24**	**(K)KEQDTSAHLERMKKNMEQTVKDLQLRKEQDTSAHLERMKKNMEQTVKDLQLR(L)**
15	**20**	**(L)RYAKFGGVHLALLPPGNYSARVRATSLAGNRYAKFGGVHLALLPPGNYSARVRATSLAGN(G)**
16	**22**	**(V)KQLAEEQKRREREQHIAEC(Carbamidomethyl)M(Oxidation)AKM(Oxidation)PQKQLAEEQKRREREQHIAEC(Carbamidomethyl)M(Oxidation)AKM(Oxidation)PQ(M)**
17	**22**	**(L)M(Oxidation)KGGSKEYWFVLTAESLSWYKDEEEKM(Oxidation)KGGSKEYWFVLTAESLSWYKDEEEK(E)**
17	**22**	**(M)GTVVLGKLESGSIFKGQQLVM(Oxidation)MPNKHNVEGTVVLGKLESGSIFKGQQLVM(Oxidation)MPNKHNVE(V)**
